# Motifs, Control, and Stability

**DOI:** 10.1371/journal.pbio.0030392

**Published:** 2005-11-15

**Authors:** John Doyle, Marie Csete

## Abstract

The interactions of networks of transcription factors and signaling molecules can be understood, in part, through concepts from control theory and engineering.

Many of the detailed mechanisms by which bacteria express genes in response to various environmental signals are well-known. The molecular players underlying these responses are part of a bacterial transcriptional regulatory network (BTN). To explore the properties and evolution of such networks and to extract general principles, biologists have looked for common themes or motifs, and their interconnections, such as reciprocal links or feedback loops. A BTN motif can be thought of as a directed graph with regulatory interactions connecting transcription factors to their operon targets (the set of related bacterial genes that are transcribed together). For example, Figure 1A shows a BTN motif that describes a part of the transcriptional response to heat (and other) stressors.

**Figure 1:**
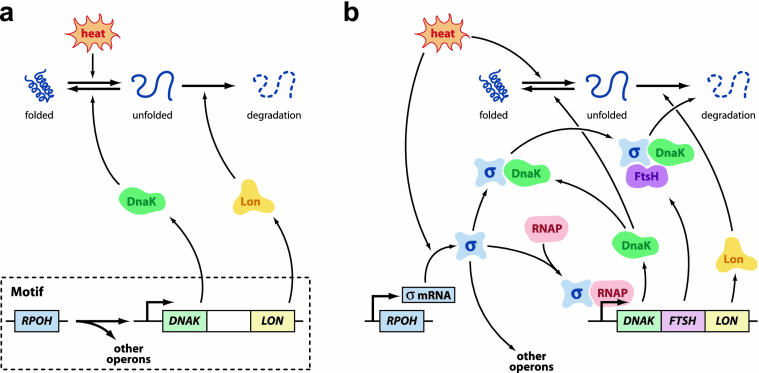
Cartoons of the Escherichia coli HS Control System (A) The transcriptional motif showing its basic function, the manufacture of chaperones to refold and proteases to degrade denatured proteins. For simplicity, only one operon is shown in detail. (B) The same network including control elements. See text for explanation.

But biological networks are not just static physical constructs, and it is, in fact, their dynamical properties that determine their function. In this issue of *PLoS Biology*, Prill et al. [1] show that the relative abundance of small motifs in biological networks, including the BTN, may be explained by the stability of their dynamics across a wide range of cellular conditions. In a dynamical system, control engineers define “stability” as preservation of a specific behavior over time under some set of perturbations. The definitions of stability vary somewhat depending on the types of system, behavior, and perturbation specified [2]. For the BTN example, Prill et al. [1] study stability of gene expression levels, as modeled by a set of linear differential equations. Given interactions from a BTN motif, “structural stability” is robustness of stability to arbitrary signs and magnitudes of interactions. This is such a stringent notion of stability that it would be satisfied by few systems, yet Prill et al. [1] show that all BTN motifs are stable for all signs and magnitudes of interactions. For several other biological networks, they show a level of correlation between abundance and structural stability that is highly unlikely to occur at random. The significance of these results as well as those in recent related papers (see references in [1], particularly those of Alon and colleagues) can be better appreciated within the larger context of well-known concepts from biology and engineering, particularly control theory [3]. For additional mathematical details underlying the qualitative arguments presented here, see the online supplement (Text S1 and S2).

## Motifs, Networks, and Dynamics

Prill et al. [1] point out that their motifs are small parts of networks in at least two distinct senses, and the heat shock (HS) response will be used to illustrate these points. A motif such as that shown in Figure 1A is just one of many motifs that make up the BTN, but is also part of an even larger network involving protein–protein interactions (PPI) illustrated in the more detailed Figure 1B [4]. The HS response ultimately works on proteins—repairing or degrading misfolded proteins before they damage the cell. The small motif cartoon of Figure 1A consists of the *rpoH* gene, which encodes a transcription factor called the alternative sigma factor σ^32^, which recognizes the HS gene promoters to induce HS-specific gene expression. For simplicity, only one operon is shown.

HS genes encode molecular “chaperones” (such as DnaK)—proteins that help refold denatured proteins—and “proteases” (such as Lon)—enzymes that degrade unfolded, dysfunctional proteins. Regulatory aspects of this motif are shown schematically in Figure 1B. Briefly, in addition to binding unfolded proteins, chaperones can also bind to σ^32^ (denoted in Figure 1 as σ), sequestering and preventing it from binding with RNA polymerase (RNAP), thus providing a negative feedback loop to modulate σ^32^ activity. The protease FtsH degrades bound σ^32^, a negative feedback that further fine-tunes the HS response. A feedforward response is implemented in the heat sensitivity of σ^32^ translation, which is enhanced at high temperatures. These additional layers of control beyond the BTN motif alone yield a system that by engineering standards is efficient, robust, and evolvable [4]. (Note: biologists use the term “regulatory” to describe networks such as that displayed in Figure 1A, but engineers typically reserve “regulatory” for actual controlling elements as in Figure 1B.)

The motif in Figure 1A is a simple tree and is perfectly structurally stable, with its stability completely independent of the specific concentrations and kinetics of the individual molecules that compose the network [1]. In contrast, by almost any reasonable definition (including appropriately generalizing the methods of Prill et al.), Figure 1B has no structural stability, as only a small subset of possible parameter values could confer a stable network. The small motif in Figure 1A is, thus, inherently stable, but Figure 1B requires a high level of fine-tuning for stability—which, in fact, has evolved for this network. An essentially parallel story holds for other motifs, as all motifs in the BTN are structurally stable. Indeed, the entire BTN from which the motifs were extracted (without the PPI elements) is perfectly structurally stable, since it has no nontrivial feedback loops (i.e., other than self-loops, where a protein regulates its own synthesis). And to the extent that analogous PPI dynamics are known for other motifs, they too require exquisite fine-tuning for stability.

**Figure 2:**
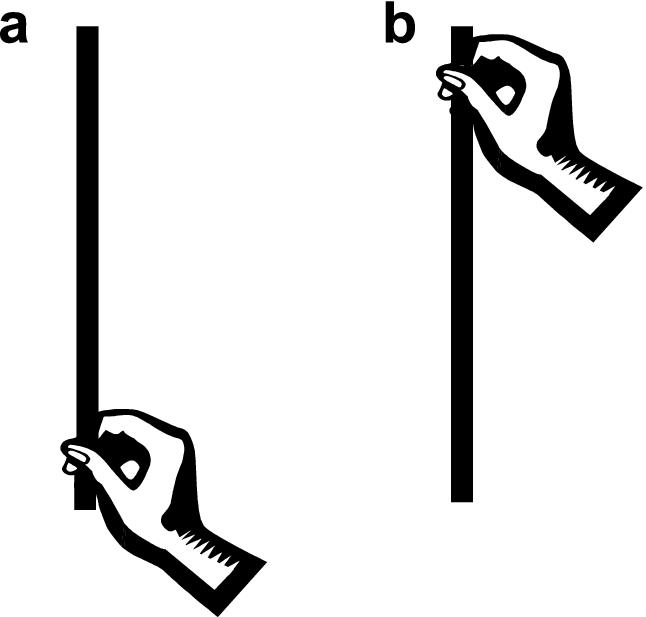
Pendulum in Up and Down Positions (A) Pendulum held in up (unstable) position (B) Pendulum held in down (stable) position

The fact that the bacterial “transcriptional networks” have such strong structural stability and that this stability is completely lost with the inclusion of protein–protein and other regulatory interactions has many possible and quite different interpretations. Structural stability is clearly not an intrinsic feature of the biology itself, but depends in a rather extreme way on the level of detail in the models chosen. Thus, based on the biology alone and the many caveats that define the way these motifs were extracted, the results based on structural stability in Prill et al. might appear to be at best speculative, and at worst misleading. We will argue, however, that their results reveal highly significant organizational principles.

## Plants, Controllers, and Disturbances

Control theory uses an abstraction that is useful in interpreting biological models like those in Figure 1 ([2]; Text S1 and S2). A system like Figure 1B is often decomposed into a “plant” (as in manufacturing plant), from which the basic function of the system can be inferred, and a “controller,” which implements feedback and feedforward manipulations to improve stability and robustness of this function. In this sense, robustness means that a specific plant function (such as low levels of unfolded proteins) is maintained in the face of certain disturbances (such as temperature or chemical insults). Robustness is usually used in a broader sense than stability, with the latter usually restricted to infinite time horizons and the former including additionally transient behavior and wider ranges of perturbations. Thus, “robust stability” is typically used to describe stability that is robust to some large set of perturbations. For example, in Figure 1A, heat can be viewed as the external disturbance on a plant consisting of folded and unfolded proteins plus chaperones and proteases. The controller in Figure 1B adds feedback and feedforward mechanisms to enhance robustness and efficiency in the control of unfolded protein levels, particularly in transient response to temperature change [4].

Many organizational features of this system have been experimentally studied. Removing σ^32^ (by creating bacteria lacking the *rpoH* gene) causes death of bacterial cells at high temperatures [5]. But this lethal knockout can be rescued by constitutive overexpression of the HS operons, essentially implementing the motif in Figure 1A as the complete system [6]. Thus, in principle, a network with the topology in Figure 1A is viable, provided it is implemented appropriately. The controller in Figure 1B enhances robustness and efficiency but is not required for basic function, and other less complex control schemes could be used, but with degraded robustness and performance compared to wild type (nonmutants) [4]. Again, to the extent that details are known, this is a common story for BTN motifs in general [7], in which a transcriptional motif provides a core plant that performs some basic function. The actual biological network, however, often has a controller involving PPI or other mechanisms, typically of much greater complexity than the motif itself, and which provides additional robustness, efficiency, and flexibility. In engineering design, controller or plant decompositions can be nested, with one plant plus controller collectively functioning as the controller for another plant, and so on. Far more complex layered control strategies are commonly used in designing technological systems, and are presumably ubiquitous in biology. For example, the hierarchical organization of bacterial regulation includes such elements as stimulons, modulons, regulons, operons, PPI control elements, etc [7].

Such decompositions are not unique, and plant and controller can, in principle, be arbitrarily chosen components, but the choices are typically used to highlight particular organizational features of the complete system. For example, Figure 1B can, instead, be decomposed into a plant consisting of just the folded or unfolded protein levels of the cell, with heat disturbance, and a controller consisting of the entire bottom part of Figure 1B. This decomposition is more natural from an engineering perspective, but highlights the BTN motif less than when Figure 1A is viewed as the plant plus disturbance. Note that because the plant motifs in the BTN involve only transcription, they typically must have much slower dynamics than the controller. For example, the slowest dynamic in Figure 1B is the synthesis of the HS operon from the plant in Figure 1A. Indeed, the rest of the controller is implemented entirely in the relatively faster PPI and σ^32^ translation, while transcription of *rpoH* is not regulated and so does not contribute to the dynamics of the controlled system [4].

## The Implications of Structural Stability

The lack of structural stability in full systems of plant and controller may lead to the speculation that they are not robust. In fact, all complex, controlled systems lack structural stability when viewed at the full system level with controller dynamics included. A complete answer to this apparent paradox is a large subject in its own right, but some aspects are easily explained. One basic point is that the signs—whether interactions are activating (positive) or inhibiting (negative)—in most biological and technological networks must be fine-tuned, but once the signs of constants are appropriately fixed, their absolute values can often vary substantially with little effect. The number of different sign combinations in *n* constants grows exponentially as 2^*n*^; thus, one (fine-tuned) choice of signs becomes a vanishingly small fraction of the total number of possibilities in any sufficiently large network. In other words, if signs are important, and they are in control systems, the resulting network cannot be structurally stable. It is also true that in both technology and biology it is much easier to manufacture components with robustly fixed signs than with precise absolute values.

Thus, while structural stability as defined in Prill et al. may be too strong of a notion to be helpful in distinguishing between different control systems, this very strength further underscores the significance of the authors' results. All the plant motifs in the BTN, indeed, the entire BTN plant itself, are not merely stable, but have extremely strong structural stability. But the necessarily fine-tuned controller can stabilize these plants, and, furthermore, unstable plants are common in biology and technology, so the absence of unstable plants here still needs further explanation.

## The Costs of Plant Instability

Perhaps the most relevant concept from control theory is that unstable plants are intrinsically more difficult to control than stable ones, and are generally avoided unless the instability confers some great functional advantage, which it often does [2,3]. A classic illustration of instability and control, the simple inverted pendulum experiment, can be easily tried at home, and illustrates the essential point without the mathematical details. Here the pendulum is the plant, and the human is the controller. The experiment can be done with sticks of different lengths or with an extendable pointer, holding the proximal tip between thumb and forefinger so that it is free to rotate but not otherwise slip. With the controlling hand fixed, this system has two equilibria, down and up, which are stable and unstable, respectively. By watching the distal tip and controlling hand motion, the up case can be stabilized if the stick is long enough. For an external disturbance, imagine that there is a virtual object making small motions in the vicinity of the distal tip, and your goal is to move the hand in such a way as to track this motion.

You will soon find that it is much easier to control the distal tip down than up, even though the components in both cases are the same. Because the up configuration is unstable, certain hand motions are not allowed because they produce large, unstable tip movements. This presents an obstacle in the space of dynamic hand movements that must be avoided, making control more difficult. If you make the stick shorter, it gets more unstable in the up case, evident in the short time it takes the uncontrolled stick to fall over. Shorter pendulums get harder and ultimately impossible to control in the up case, while length has little such effect on the down case. Also, the up stick cannot be stabilized for any length if only the proximal tip is watched, so the specific sensor location is crucial as well. This exercise is a classical demonstration of the principle that the more unstable a system the harder it is to control robustly, and control theory has formally quantified this effect in several ways (Text S1).

With this general context, a plausible conjecture is that the stability of the slow dynamics in the BTN plant is there, in part, to make control easier. It is the controller in Figure 1B that must be robust to the plant and disturbance in Figure 1A, not the other way around. Yet as the pendulum example illustrates, the stability of the plant can have a large impact on the achievable robustness of the controller. The BTN plant stability could additionally be a consequence of evolutionary constraints, in that the slow dynamics may have existed first (such as Figure 1A), and control was later layered (as in Figure 1B). If the slow dynamics are simply vestiges of an original, uncontrolled, and structurally stable network, their preservation, even by accident, still facilitates the full system level control. Perhaps such preservation could be the result of selective pressure on this system for robustness.

## A Place for the Unstable Plant

While unstable plants are difficult to control, they are used when function requires it. Modern rockets are unstable in a manner similar to the up pendulum, and must be stabilized by active control systems. Toy rockets or fireworks without active control create stable plants by using large fins or tails that passively move the centers of pressure and gravity to make the dynamics more like the down pendulum, but at the expense of greater drag. Technology has abundant examples where similar efficiency and performance trade-offs lead to unstable plants with actively stabilizing controllers. Even bacteria have systems with unstable components that are nevertheless combined into feedback systems that are stable and robust.

**Figure 3:**
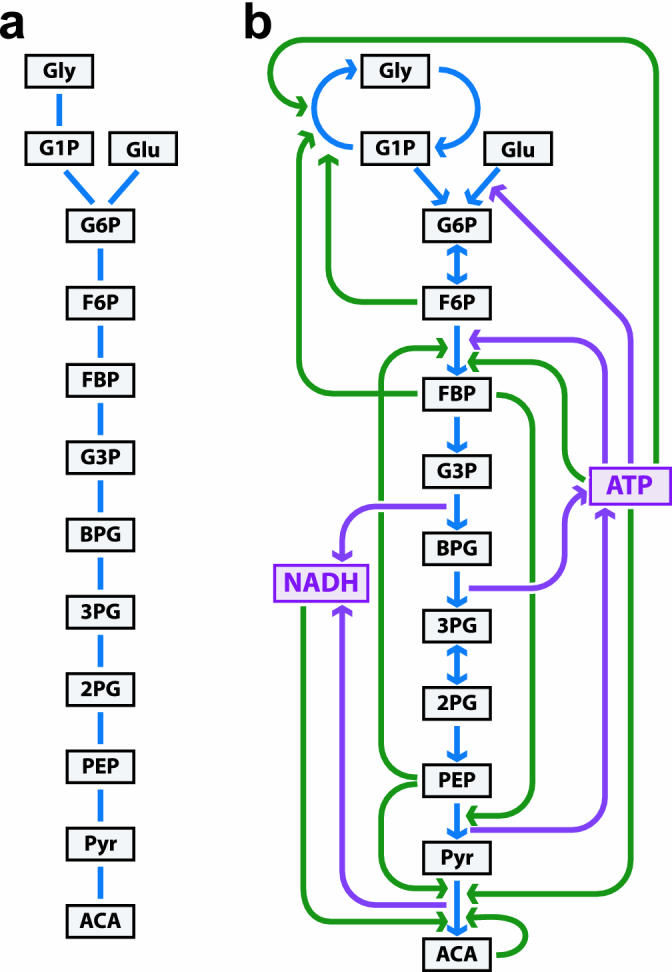
Cartoons of a Generic Glycolysis Network in Bacteria (A) A simple graph showing the main metabolites of glycolysis and their relationships. (B) The same metabolites but also including reactions (solid blue lines), autocatalytic feedbacks (solid purple lines), and regulatory feedbacks (dotted black lines).

Chemotaxis, cell movement toward a chemical attractant, is an example whereby an uncontrolled plant consisting of only cell and flagella would move essentially randomly, and, thus, would not be stable in any conventional sense. Yet with the full signal transduction system in place, the controlled runs and tumbles are biased to create effective chemotaxis, apparently using strategies common in engineering [8]. Glycolysis is often drawn as a “molecular motif” as in Figure 3A, without loops and therefore structurally stable, and the relationship between Figure 3A and the larger controlled Figure 3B is even more subtle than between Figure 1A and 1B. Figure 3B shows both positive autocatalytic feedback of ATP needed to fuel glycolysis and negative regulatory feedback, a combination that when sufficiently perturbed can lead to well-known instabilities, even with the control system intact [9]. These complex features of HS, chemotaxis, and glycolysis may not be accidental, but may be necessary consequences of unavoidable trade-offs, and as briefly sketched here, control theory supports this notion. Perhaps more persuasive is that these are three of the most thoroughly studied small networks in biology, and apparently no one has found alternatives, even theoretically, that convincingly improve on the efficiency and robustness of the wild type networks.

BTN motifs exhibit an extremely strong version of structural stability. Yet because of the organization involving both plant and controller, this apparently severe restriction on the BTN plants does not necessarily create a correspondingly severe constraint on function. For example, basic function of the plant motif in Figure 1A of manufacturing HS proteins is simple enough that only a minimal network is needed. Most of the network complexity is in the controller in Figure 1B, and to maximize the speed of the HS response, it is important to minimize the effects of any additional transcriptional events, which implies the plant must be kept simple. The result is that the BTN network as a whole is very flat with few long paths, which we conjecture by analogy to HS, allows the controlled system to have rapid response [10]. Perhaps a constructive next step would be to systematically contrast the strongly stable BTN plants with the less stable plants in chemotaxis and glycolysis. From an engineering perspective, all of these well-studied bacterial networks appear highly efficient and robust, tolerating trade-offs to achieve this well-engineered state [11]. And now, Prill et al. put at least one feature of bacterial transcriptional network motifs, their structural stability, into a much broader context.

## Supporting Information

Text S1 Supplementary Notes: Elementary Feedback Concepts (154 KB PDF).

Text S2 Feedback Control Theory (4.2 MB PDF).

## Abbreviations

BTN: bacterial transcriptional regulatory network
HS: heat shock
PPI: protein–protein interactions
