# Supplementary material for: Motifs, Control, and Stability
Source: PLoS Biol. 2005 Nov 15;3(11):e392. doi: 10.1371/journal.pbio.0030392 (PMC1283396; doi:10.1371/journal.pbio.0030392)
Supplement: Text S1 — (154 KB PDF). [file pbio.0030392.sd001.pdf]

Supplementary Notes: Elementary Feedback Concepts

John C. Doyle, Marie E. Csete

Abstract: Convergent evolution in both biology and technology produces modular architectures with elaborate hierarchies of protocols and layers of feedback regulation, driven by demand for robustness to uncertain environments, while using often-imprecise components. This tutorial reviews the most elementary and well-known results from control theory of relevance to understanding feedback in biological networks.

A key starting point in developing a conceptual and theoretical bridge between engineering and biology is robustness, the preservation of particular characteristics despite uncertainty in components or the environment (*1*,*2*). Biologists and biophysicists new to studying complex networks often express surprise at a biological network's apparent robustness (*3*). They find "perfect adaptation" and homeostatic regulation are robust properties of networks (*4*,*5*), despite "exploratory mechanisms" that can seem gratuitously uncertain (*6*,*7*,*8*). Some even conclude that these mechanisms and their resulting features seem absent in engineering (*8*,*9*). However, ironically, it is in the nature of robustness and complexity where biology and advanced engineering are *most* alike (*10*). Good design in both cases (e.g. cells and bodies, cars and planes) means users are largely unaware of hidden complexities, except through system failures. Furthermore, the robustness and fragility features of complex systems are shared *and* necessary. Although the need for universal principles of complexity and corresponding mathematical tools is widely recognized (*11*), sharp differences arise as to what is fundamental about complexity and what mathematics is needed (*12*). This tutorial is based on (*2*) and presents the most elementary aspects of well-known results in control theory.

Protocols are the most important aspect of modularity, and the most complex and critical protocols are for feedback control and the sensing, computing, communication, and actuation that implement it. Feedback control is both a powerful and dangerous strategy for creating robustness to external disturbances and internal component variations. Properly balanced it delivers such a huge benefit that both engineers and evolution capitalize extensively on feedback to build and support complex systems. Detailed elaboration of the nature of regulatory feedback is well beyond the scope of this tutorial, but an elementary "toy" model illustrates the *necessity* both of feedback to the function of complex systems and its "conservation of fragility" law. This is arguably the most critical and rigorously established robustness tradeoff in complex systems.

In most technologies as well as biochemistry it is relatively easy to build either uncertain, high gain components, or precise, low gain ones, but the precise, high gain systems essential to both biology and technology are impossible or prohibitively expensive to make, except using feedback strategies such as in Fig. 1. The simplest case to analyze is steady state gain, where after some transient, r and d are held constant, and y too approaches a constant $y=Rr+Sd$ (*13*). Solving $y=d+ACy+Ar$ gives

$$y = ASr + Sd = \frac{1}{C}(S-1)r + Sd$$

$$S \triangleq \frac{1}{1-F} \qquad F \triangleq AC$$

Ideally, perfect control would have $|S| = 0$, since that gives $y = -r/C$ ($R = -1/C$) completely independent of arbitrary variations in A and d. This can be achieved asymptotically if $A \to \infty$ and $-1/C >> 1$ then $F \to -\infty$, $|S| \to 0$, and $y \to r/C$. Then R amplifies r and is perfectly robust to external disturbance d and to variations in A (*14*). Choosing C small and precise, with A sufficiently large and even sloppy, is one effective, efficient, and robust way to make y a high gain function of r. $|S|$ measures the deviation from perfect control, and feedback can attenuate or greatly amplify the effects of uncertainties. Defining *fragility* as $\log|S|$, note that $F < 0$ iff $|S| < 1$ iff $\log|S| < 0$. $F > 0$ makes $\log|S| > 0$, amplifying d and uncertainty in A, and $F \to 1$ makes $\log|S| \to \infty$ (*15*).

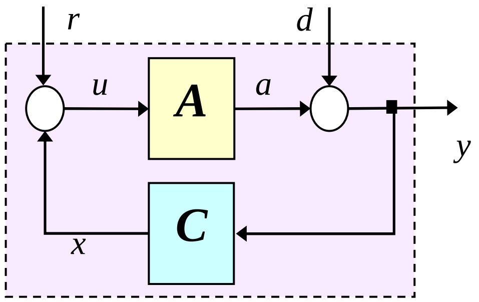

Figure 1. Minimal feedback system with actuator *A* and controller/sensor *C*. Goal is for response *y* to amplify reference *r*, independent of external disturbance *d*, and variations in *A.*

Dynamics

This story is incomplete and even misleading without dynamics. The simplest possibility is for *A* and *C* to be 1st-order differential equations

$$\begin{aligned} C: x' &= -k_1 y - k_2 x \quad & y = d + a \\ A: a' &= gu \quad & u = r + x \end{aligned} \qquad (1)$$

C is a low pass filter with internal state *x* and parameters k_1 and k_2. *A* is a pure integrator with state *a* and gain *g* (*16*), and we'll assume that $g > 0$. This system can be written in "state space" form as

$$\begin{bmatrix} x' \\ a' \end{bmatrix} = \begin{bmatrix} -k_2 & -k_1 \\ g & 0 \end{bmatrix} \begin{bmatrix} x \\ a \end{bmatrix} + \begin{bmatrix} -k_1 d \\ gr \end{bmatrix} \qquad (2)$$

Stability depends on the eigenvalues λ_j of the matrix

$$\begin{bmatrix} -k_2 & -k_1 \\ g & 0 \end{bmatrix}$$

which are the zeros of the characteristic polynomial

$$s^2 + k_2 s + g k_1$$

having negative real part ($\mathrm{Re}(\lambda_j) < 0$). It is easily checked that this holds and the system is stable iff $gk_1>0$ and $k_2 > 0$, or equivalently $k_1 > 0$ and $k_2 > 0$. Stability here means the solution in the states *x* and *a* converges to the origin for all initial conditions, and any bounded input in *r* and/or *d* gives bounded state. For this system these two notions are equivalent, but in general there are a variety of notions of stability.

Thus given $g > 0$, stability depends only on the signs of k_1 and k_2, and holds if and only if both are positive. Since there are only two constants, this means that ¼ of the space of values is stable, but then it is stable for all positive values. Thus stability is not robust to sign changes, but with fixed signs is very robust to magnitude changes. This is a typical situation and has apparently been a large source of confusion regarding the robustness of biological networks. For *n* constant, the number of different sign combinations grows exponentially as 2^n and thus one (fine-tuned) choice of signs becomes a vanishingly small fraction of the total number of possibilities in any sufficiently large network. For example, a characteristic polynomial such as

$$s^n + k_n s^{n-1} + \cdots + k_2 s + k_1$$

has roots with negative real part only if all k_j are positive, although additional (polynomial) constraints are needed to give if and only if conditions. In other words, if signs are important, and they are in control systems, the resulting network cannot be structurally stable. It is also true that in both technology and biology it is much easier to manufacture components with robustly fixed signs than with precise absolute values, so this is not necessarily a stringent constraint on control systems. Much more important is that this type of stability is not as important as the more severe constraint of robust transient response.

This type of control is called "integral feedback." The parameters *g*, k_1, and k_2 might typically be functions of underlying physical quantities such as temperature, binding affinities, concentrations etc. and thus might vary widely. The response $y(t)$ to steps in *r* and *d* are shown in Figure 2 over two orders of magnitude in *g* and $k_1 > 0$.

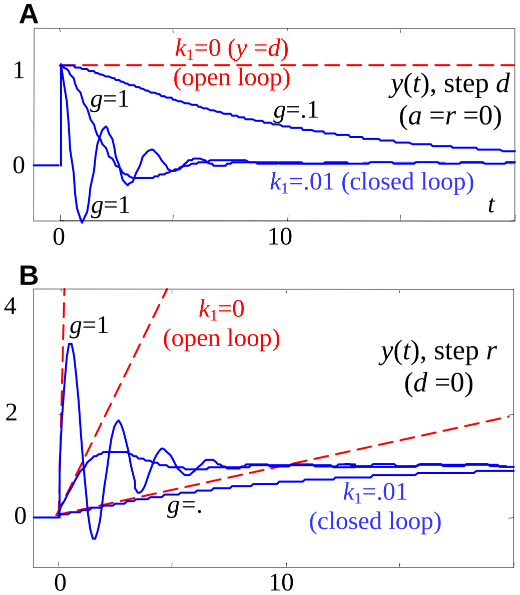

Figure 2. Closed (k_1=0, blue) vs. open (k_1=.01, red) loop response $y(t)$ to step changes at t=0 in **(A)** $d(t)$ (r=0) and **(B)** $r(t)$ (d=0) for g=.1, 1, 10, k_1=.01, k_2=10 k_1. Note the extreme divergence (k_1=0) vs. convergence (k_1=.01) as $t\to\infty$.

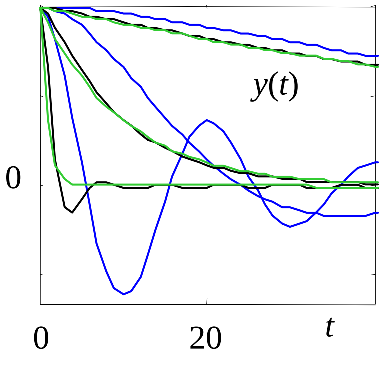

Figure 3. Zoom of Figure 2A with k_1=.01 (blue), .1 (black), 1 (green), and k_2=10k_1 added for each value of g.

This simple protocol of *integral feedback* produces extremely robust external behavior even from wildly varying components (the blue solid versus red dashed lines in Figure 2b) and converges to the steady state $y=(k_2/k_1)r$ independently of *arbitrarily* large variations in gain g and disturbance d (*17*). If $k_2>>k_1$, $y=(k_2/k_1)r$ is a high gain amplifier as well (*18*). The individual values of g, k_1 and k_2 influence the rate of convergence to steady state, but only (k_2/k_1) determines its value. Thus robust high steady state gain can be achieved with uncertain and small parameters with the right feedback protocol. Figure 3 shows that variations in both g and k_2 of orders of magnitude have modest impact, and only on early transient behavior.

The *protocol* here is the structure of the equations, including the integral feedback and the signs of the parameters. Modules are the implementations of the actuator and controller. As with the Lego example in (*2*), this protocol must be "fine-tuned" (since rewiring components or flipping signs typically creates exponentially growing instabilities), but this allows the modules to vary widely with minimal effect (*19*). Integral feedback is used ubiquitously in engineering (*20*) and is likely to be ubiquitous in biology as well, to achieve everything from homeostatic regulation to "perfect adaptation," and preliminary investigations confirm this impression (*21*,*22*,*23*). One reason is that integral feedback is both sufficient *and necessary* for perfect and robust steady state tracking. Intuitively, necessity follows from the fact that in steady state, $a=y-d$ must perfectly cancel *any* constant (step in) d, while the input u to A cannot depend on this d, since y does not. Thus, A (or C) must contain an *internal model* of the *dynamics* of d, which for step changes is a pure integrator (*24*), which produces unbounded outputs to constant inputs. Thus, open-loop hypersensitivity is necessary for closed loop robustness, and the behavior in Figures 2 and 3 is not an accident.

Conservation laws

Fragility also enters in the transient response. When g is increased, the response is faster but oscillatory (Figure 2). Figure 4 plots fragility $\log|S(\omega)|$ versus ω where $Y(\omega)$ and $D(\omega)$ are Fourier

$$\log|S(\omega)| = \log\left|\frac{Y(\omega)}{D(\omega)}\right| \qquad (3)$$

transforms of y and d. For increasing g, low frequency robustness ($\log|S(\omega)|<0$) is improved but at the expense of increased fragility ($\log|S(\omega)|>0$) at higher frequencies (*25*). In fact, it can be proven that for all g

$$\int_0^\infty \log|S(\omega)|\, d\omega = 0 \qquad (4)$$

so net fragility is, in this sense, a conserved quantity.

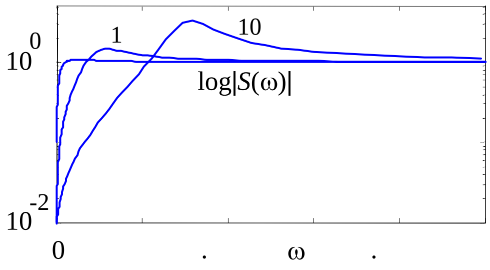

Figure 4. log**|**$S(\omega)$**| vs.** ω for responses in 2A). The peaks in log**|**$S(\omega)$**|** correspond to the oscillations in 2A) and 2B). Note the equal areas under the curves for log**|**$S(\omega)$**|.**

Robustness ($\log|S(\omega)|<0$) is paid for by an equal fragility ($\log|S(\omega)|>0$) which amplifies d and uncertainty in A (*26*). This quite general result also holds for arbitrary parameters, control systems, and disturbances (*27*). Thus there are always nonconstant (e.g. sinusoidal) $d(t)$ that would be *amplified* in $y(t)$. Such d could be perfectly rejected too, but only by adding internal models as *complex as the external environment* that generates d. While such modeling is only possible for simple idealized laboratory environments, even approximate attempts can drive an extreme complexity spiral in real systems, and *any* controller is still subject to the constraint in eq. (4). The key to good control design then is insuring that this fragility is tolerable, and occurs where uncertainties are relatively small.

Even these simple toy examples show the robust yet fragile features of complex regulatory networks. Their outward signatures are, ironically, extremely constant regulated variables *yet* occasional cryptic fluctuations. They have extraordinary robustness to component variations *yet* rare but catastrophic cascading failures. These apparently paradoxical combinations can easily be a source of confusion to experimentalists, clinicians, and theoreticians alike (*28*), but are intrinsic features of highly optimized feedback regulation. Since net robustness and fragility are constrained quantities, they must be manipulated and controlled with and within complex networks, even more than energy and materials. Figure 2b shows how extreme open vs. closed loop behavior can be, and thus how dangerous loss of control is to a system relying on it. The tradeoff in equation (4) shows that even when working perfectly, net fragility is constrained, and thus some transient amplification is unavoidable.

The *necessity* of integral feedback and the fragility constraint in equation (4) thus describe *laws*, not protocols, perhaps the two simplest such laws from control theory. Controllers that are more complex, with additional dynamics and multiple sensors and actuators, offer more refinement in performing robustness-fragility tradeoffs. Adding to regulatory complexity is also evolutionarily relatively easy. Faster components allow for faster closed-loop responses. All are used in both biology and engineering but all are still ultimately subject to equation (4). Control engineers must contend with this tradeoff, and its generalizations to more complex structures dominate control system design. It may be that such tradeoffs dominate and constrain evolution and biology as well.

The cost of instability

The simplest change that introduces plant instability is for A to be changed to

$$C: x' = -k_1 y - k_2 x \quad y = d + a$$

$$A: a' = \sigma a + gu \quad u = x$$

where for simplicity r has been eliminated and C is unchanged, and we'll continue to assume that $g > 0$ and also that $\sigma \geq 0$. With $k_1 = k_2 = 0$ there is no feedback, and the system is "open loop" with an unstable pole at σ. The state space form is

$$\begin{bmatrix} x' \\ a' \end{bmatrix} = \begin{bmatrix} -k_2 & -k_1 \\ g & \sigma \end{bmatrix} \begin{bmatrix} x \\ a \end{bmatrix} + \begin{bmatrix} -k_1 d \\ 0 \end{bmatrix}$$

Stability again depends on the eigenvalues of the matrix

$$\begin{bmatrix} -k_2 & -k_1 \\ g & \sigma \end{bmatrix}$$

or the zeros of the characteristic polynomial

$$s^2 + (k_2 - \sigma)s + (gk_1 - k_2\sigma)$$

so this system is asymptotically stable iff

$$k_2 > \sigma \qquad g\frac{k_1}{k_2} > \sigma$$

Thus the region of stability shrinks as σ gets larger. The more serious limitation is that equation (4) now becomes

$$\int_0^\infty \log|S(\omega)|\,d\omega \geq \pi\sigma \qquad (5)$$

so that the tradeoff in equation (4) is aggravated. While unstable plants are common in engineering and biology, they are typically used only when efficiency or performance demands it, and otherwise are avoided since unstable plants are intrinsically harder to control.

Implications for biology and engineering

Success of systems biology will certainly require modeling and simulation tools from engineering (*29,30*), where experience shows that brute force computational approaches are hopeless for complex systems involving protocols and feedback. Highly fragile features require highly sophisticated modeling, whereas robust features often have adequate models that are greatly simplified. For example, if Fig. 1 was for a module in a larger system, the steady-state gain $y=(k_2/k_1)r$ depends only on (k_2/k_1) and no other parameters, potentially simplifying experiments and modeling. If transient dynamics or component failure were of interest, more details would be needed, determined more by the rest of the system than by the internal components.

Many challenges of post-genomic biology are converging to those facing engineers building complex networks and "systems of systems." Engineering theory and practice are now undergoing a revolution as radical as biology's. The simple ideas here only hint at the possibilities. For example, more complex control protocols than Figure 1, used in both engineering and biology, can ameliorate though not eliminate the constraint in (4), but sophisticated theory is needed to elucidate the issues. Realistic models of biological networks will not be simple, with multiple feedback signals, nonlinear component dynamics, numerous uncertain parameters, stochastic noise models (*31*), parasitic dynamics, and other uncertainty models. Scaling to deal with large networks will be a major challenge. Fortunately, researchers in robust control theory, dynamical systems, and related areas have been vigorously pursuing mathematics and software tools to address exactly these issues and apply them to complex engineering systems (*32,33*). Biological applications are new, but progress so far is encouraging.

Experiments, modeling and simulation, and theory all have fragilities, but they are complementary, and through the right protocols, have the potential to create a robust "closed-loop" systems biology. Biologist's frustrating experience with theory has been primarily in an open-loop mode, where simple and attractive ideas can be wrong but receive enormous attention. Biology is the only science where feedback control and protocols play a dominant role, so it should not be surprising that there would be popular theories, coming from within science, which did not emphasize these issues. Biologists and engineers now have enough examples of complex systems that they can close the loop and eliminate specious theories (33). For example, Internet technology is rich in protocols and feedback, and a deep, rigorous, and practically relevant theory has recently been developing. Even though it is poorly understood by nonexperts and has become a focus of many specious theories, details and enormous data sets are available, and it makes an

attractive example to compare with biological networks(see additional references).

Notes

1. Complex systems may be nonlinear, heterogeneous, large-scale, hierarchical, adaptive etc. but these are subsidiary issues here.
2. Csete M, Doyle J, Reverse Engineering of Biological Complexity, *Science* 295 (5560): 1664-1669 Mar 1 2002
3. Fragility here has the specific meaning of large and deleterious changes in particular system properties due to possibly small but specific variations in the environment or in components. Robustness is often used more broadly, but here is roughly the inverse of fragility. Their subtle interplay is the point of this paper.
4. Alon, U., Surette, M.G., Barkai, N., Leibler, S. *Nature* 397, 168-171 (1999).
5. von Dassow, et al. Nature 406, 188-192 (2000).
6. Gerhart, J. and Kirschner, M., *Proc. Natl. Acad. Sci*. 95, 8420-27 (1998).
7. E.g. chemotaxis, immune response, microtubule formation, angiogenesis, neurogenesis, and evolution itself. See (*6*)
8. Kirschner, M., Gerhart, J., Mitchison, T. *Cell* 100, 79-88 (2000).
9. Chicurel, M. *Nature* 408, 900-901 (2000).
10. This has a long history, see for example Wiener, N. *Cybernetics*, (New York, Wiley, 1948)
11. "Complex Systems" Special Issue, *Science*, V 284, No. 5411 (1999)
12. Carlson, J.M., Doyle, J. *Proc. Natl. Acad. Sci*. 99, (2002)
13. Steady state here means simply that all variables in Figure 2 (r, d, y, A, C, etc) approach constants, which can be solved for algebraically.
14. ">>" means "very much greater than."
15. An important use of positive feedback is to *deliberately* destabilize equilibria and amplify small differences to create switches and break symmetries and homogeneities. This can create patterns then maintained using negative feedback. Positive feedback is also critical to autocatalysis in growth and metabolism.
16. $a'=gu$ means a (the output of A) is a time integral of gu, where u is the input to A.
17. Stability is easily shown using standard methods of linear systems. Steady state values can be found (in a stable system) by setting all time derivatives to 0, yielding $gk_1y=gk_2r$ or $y=(k_2/k_1)r$.
18. Mechanisms often exist that allow controller parameters (e.g. k_1 and k_2) to be much less uncertain than g and d. It is often even easier to make *ratios* such as (k_2/k_1) largely invariant to variations in underlying physical quantities affecting the individual k_1 and k_2.
19. Except (k_2/k_1) if precise gain is desired.
20. The national power grid has integral control at the >3,000 power plants to regulate frequency and voltages of delivered power, oil refineries have >10,000 such control loops, and Internet congestion control involves a form implemented as part of TCP (Transport control protocol). See additional references for more details, proofs, and examples.
21. Yi, T.M., Huang, Y., Simon, M.I., Doyle, J. . *Proc. Natl. Acad. Sci*. 99, 4649-4653 (2000).
22. Levchenko, A., Iglesias, P. A. *Biophys. J.* 82, 50-63 (2002).
23. El-Samad, H, Goff JP, Khammash, *J. Theo. Bio*. pp. 17-29, Vol. 214, No. 1, (2002)
24. This argument can be made rigorous and is a standard elementary result in control theory. It is a special case of the internal model principle.
25. For sufficiently large g the frequency domain peak and time domain transients become unacceptably large, though still stable.
26. One interpretation is that negative feedback is always balanced by an equal and opposite positive feedback. Strictly speaking, with dynamics this is not well defined, and $\log|S(\omega)|$ gives the correct generalization.
27. Bode, H.W., *Network Analysis and Feedback Amplifier Design* (1945). Relatively rare circumstances can involve an inequality (≥). This is worse, but means (4) is an inequality constraint rather than a pure "conservation" law.
28. The robust yet fragile nature of highly optimized complex regulatory networks can be mistakenly attributed to various kinds of bifurcations and "order-disorder" transitions (e.g. phase transitions, critical phenomena, "edge-of-chaos," pattern formation, scale-free, etc.).
29. E.g. the Boeing 777 alone required a global software and computing infrastructure with roughly ten thousand workstations, terabytes of data, and a billion dollar price tag.
30. www.sbml.org
31. Gillespie, D.T. *J. Chem. Phys*. 113, 297-306 (2000).
32. Zhou, K., Doyle, J., Glover, K. *Robust and Optimal Control* (Prentice-Hall, Upper Saddle River, NJ, 1996).
33. Doyle et al, (2005), The "Robust Yet Fragile" Nature of the Internet, *P Natl Acad Sci* USA. vol. 102 no. 41, October 11, 2005

Additional references

1. Doyle JC, Carlson JM, Power laws, highly optimized tolerance, and generalized source coding, *Phys Rev Lett* 84 (24): 5656-5659 JUN 12 2000
2. Yi T-M, Huang Y, Simon MI, Doyle J. Robust perfect adaptation in bacterial chemotaxis through integral feedback control, *Proc. Natl. Acad. Sci.* USA 97, 4649-4653, 2000.
3. Finney AM, Hucka M, Sauro HM, Doyle J, Kitano H, Bolouri H, The Systems Biology Markup Language *Molecular Biology of the Cell* 12: 708 Suppl. S NOV 2001
4. Zhu X, Yu J, Doyle J, Heavy Tails, Generalized Coding, and Optimal Web Layout, *Infocomm*, 2001
5. Zhou T, Carlson JM, Doyle J, Mutation, specialization, and hypersensitivity in highly optimized tolerance, *Proc. Natl. Acad. Sci.* USA 99 (4): 2049-2054 Feb 19 2002
6. Morohashi M, Winn AE, Borisuk MT, Bolouri H, Doyle J, Kitano H, Robustness as a measure of plausibility in models of biochemical networks, *Journal Of Theoretical Biology* 216 (1): 19-30 MAY 7 2002
7. Carlson JM, Doyle J, Complexity and robustness, *P Natl Acad Sci* USA 99: 2538-2545 Suppl. 1 FEB 19 2002
8. Low SH, Paganini F, Doyle JC, Internet congestion control, *IEEE Contr Syst Mag* 22 (1): 28-43 FEB 2002
9. Hucka M, Finney A, Sauro HM, Bolouri H, Doyle JC, The systems biology markup language (SBML): a medium for representation and exchange of biochemical network models, *BIOINFORMATICS* 19 (4): 524-531 MAR 1 2003
10. Papachristodoulou, A; Li, L; Doyle, JC, 2004. Methodological frameworks for large-scale network analysis and design, *COMPUTER COMMUNICATION REVIEW*, JUL 2004
11. Li, L., D. Alderson, J. Doyle, and W. Willinger. 2004. A First-Principles Approach to Understanding the Internet's Router-level Topology. *Proc. ACM SIGCOMM, COMPUTER COMMUNICATION REVIEW*, OCT. 2004.
12. Csete M.E. and J.C. Doyle, 2004, Bow ties, metabolism, and disease, *Trends in Biotechnology*, Vol 22, Issue 9, pg. 446-450

13. J. Stelling, U. Sauer, Z. Szallasi, F. J. Doyle III, and J. Doyle, 2004, Robustness of cellular functions, *Cell*, October, 2004.
14. Willinger, W, D. Alderson, J.C. Doyle, and L.Li., 2004. More ``Normal" Than Normal: Scaling Distributions and Complex Systems. *Proceedings of the 2004 Winter Simulation Conference.*
15. Shapiro, BE; Hucka, M; Finney, A; Doyle, J. 2004. MathSBML: a package for manipulating SBML-based biological models, *BIOINFORMATICS*, NOV 1, 2004
16. Doyle, J; Csete, M, 2004. Imitation of life: How biology is inspiring computing, *NATURE*. OCT 21, 2004
17. Kitano, H; Oda, K; Kimura, T; Matsuoka, Y; Csete, M; Doyle, J; Muramatsu, M, 2004 .Metabolic syndrome and robustness tradeoffs, *DIABETES* 53: S6-S15 Suppl. 3, DEC 2004
18. H. El-Samad, H. Kurata , J.C. Doyle , C.A. Gross, and M. Khammash, 2005, Surviving Heat Shock: Control Strategies for Robustness and Performance, *P Natl Acad Sci* USA (8): 2736-2741 FEB 22, 2005
19. Jin C, Wei D, Low SH, Bunn J, Choe HD, Doyle JC,et al, FAST TCP: From theory to experiments *IEEE NETWORK* 19 (1): 4-11 JAN-FEB 2005
20. Paganini F, Wang ZK, Doyle JC, et al. Congestion control for high performance, stability, and fairness in general networks , *IEEE-ACM TRANSACTIONS ON NETWORKING* 13 (1): 43-56 FEB 2005
21. W. Willinger, J Doyle, Robustness and the Internet: Design and evolution, in *Robust Design: A Repertoire of Biological, Ecological, and Engineering Case Studies* (Santa Fe Institute Studies on the Sciences of Complexity), Erica Jen, Editor
22. L. Chen; Low, S.H.; Doyle, J.C.(2005) ; Joint congestion control and media access control design for ad hoc wireless networks, *INFOCOM* 2005. Volume 3, 13-17 March 2005
23. M. Manning, J.M. Carlson, and J. Doyle (2005), Highly Optimized Tolerance And Power Laws In Dense And Sparse Resource Regimes, *Phys. Rev. E*. E 72, 016108
24. J. Wang; L. Li; Low, S.H.; Doyle, J.C. (2005); Cross-layer optimization in TCP/IP networks, *IEEE/ACM Transactions on Networking*, Volume 13, Issue 3, June 2005
25. Martins, NC; Dahleh, MA; Doyle, JC (2005), Fundamental Limitations of Performance in the Presence of a Remote Early Warning System, to appear, *2005 Conference on Decision and Control*.
26. R. Tanaka, T-M Yi, and J. Doyle, Some protein interaction data do not exhibit power law statistics, FEBS letters, to appear
27. Doyle et al, (2005), The “Robust Yet Fragile” Nature of the Internet, P Natl Acad Sci USA. vol. 102 no. 41, October 11, 2005
28. L Li, D Alderson, JC Doyle, W Willinger (2005) Towards a Theory of Scale-Free Graphs: Definition, Properties, and Implications, *Internet Math*, to appear
