# Supplementary material for: Motifs, Control, and Stability
Source: PLoS Biol. 2005 Nov 15;3(11):e392. doi: 10.1371/journal.pbio.0030392 (PMC1283396; doi:10.1371/journal.pbio.0030392)
Supplement: Text S2 — (4.2 MB PDF). [file pbio.0030392.sd002.pdf]

Selected pages from

# Feedback Control Theory

John Doyle, Bruce Francis, Allen Tannenbaum

©Macmillan Publishing Co., 1990

Copyright held by authors

# Contents

**Preface** **iii**

**1 Introduction** **1**
1.1 Issues in Control System Design . . . 1
1.2 What Is in This Book . . . 7

**2 Norms for Signals and Systems** **11**
2.1 Norms for Signals . . . 11
2.2 Norms for Systems . . . 13
2.3 Input-Output Relationships . . . 15
2.4 Power Analysis (Optional) . . . 16
2.5 Proofs for Tables 2.1 and 2.2 (Optional) . . . 18
2.6 Computing by State-Space Methods (Optional) . . . 21

**3 Basic Concepts** **27**
3.1 Basic Feedback Loop . . . 27
3.2 Internal Stability . . . 30
3.3 Asymptotic Tracking . . . 33
3.4 Performance . . . 35

**4 Uncertainty and Robustness** **39**
4.1 Plant Uncertainty . . . 39
4.2 Robust Stability . . . 43
4.3 Robust Performance . . . 47
4.4 Robust Performance More Generally . . . 51
4.5 Conclusion . . . 52

**5 Stabilization** **57**
5.1 Controller Parametrization: Stable Plant . . . 57
5.2 Coprime Factorization . . . 59
5.3 Coprime Factorization by State-Space Methods (Optional) . . . 63
5.4 Controller Parametrization: General Plant . . . 64
5.5 Asymptotic Properties . . . 66
5.6 Strong and Simultaneous Stabilization . . . 68
5.7 Cart-Pendulum Example . . . 73

**6 Design Constraints** **79**
6.1 Algebraic Constraints . . . 79
6.2 Analytic Constraints . . . 80

**7 Loopshaping** **93**
7.1 The Basic Technique of Loopshaping . . . 93
7.2 The Phase Formula (Optional) . . . 96
7.3 Examples . . . 100

**8 Advanced Loopshaping** **107**
8.1 Optimal Controllers . . . 107
8.2 Loopshaping with $C$ . . . 108
8.3 Plants with RHP Poles and Zeros . . . 113
8.4 Shaping $S$, $T$, or $Q$ . . . 125
8.5 Further Notions of Optimality . . . 128

**9 Model Matching** **139**
9.1 The Model-Matching Problem . . . 139
9.2 The Nevanlinna-Pick Problem . . . 140
9.3 Nevanlinna's Algorithm . . . 143
9.4 Solution of the Model-Matching Problem . . . 147
9.5 State-Space Solution (Optional) . . . 149

**10 Design for Performance** **153**
10.1 $P^{-1}$ Stable . . . 153
10.2 $P^{-1}$ Unstable . . . 158
10.3 Design Example: Flexible Beam . . . 159
10.4 2-Norm Minimization . . . 164

**11 Stability Margin Optimization** **169**
11.1 Optimal Robust Stability . . . 169
11.2 Conformal Mapping . . . 173
11.3 Gain Margin Optimization . . . 174
11.4 Phase Margin Optimization . . . 179

**12 Design for Robust Performance** **183**
12.1 The Modified Problem . . . 183
12.2 Spectral Factorization . . . 184
12.3 Solution of the Modified Problem . . . 185
12.4 Design Example: Flexible Beam Continued . . . 191

**References** **197**

# Preface

Striking developments have taken place since 1980 in feedback control theory. The subject has become both more rigorous and more applicable. The rigor is not for its own sake, but rather that even in an engineering discipline rigor can lead to clarity and to methodical solutions to problems. The applicability is a consequence both of new problem formulations and new mathematical solutions to these problems. Moreover, computers and software have changed the way engineering design is done. These developments suggest a fresh presentation of the subject, one that exploits these new developments while emphasizing their connection with classical control.

Control systems are designed so that certain designated signals, such as tracking errors and actuator inputs, do not exceed pre-specified levels. Hindering the achievement of this goal are uncertainty about the plant to be controlled (the mathematical models that we use in representing real physical systems are idealizations) and errors in measuring signals (sensors can measure signals only to a certain accuracy). Despite the seemingly obvious requirement of bringing plant uncertainty explicitly into control problems, it was only in the early 1980s that control researchers re-established the link to the classical work of Bode and others by formulating a tractable mathematical notion of uncertainty in an input-output framework and developing rigorous mathematical techniques to cope with it. This book formulates a precise problem, called the *robust performance problem*, with the goal of achieving specified signal levels in the face of plant uncertainty.

The book is addressed to students in engineering who have had an undergraduate course in signals and systems, including an introduction to frequency-domain methods of analyzing feedback control systems, namely, Bode plots and the Nyquist criterion. A prior course on state-space theory would be advantageous for some optional sections, but is not necessary. To keep the development elementary, the systems are single-input/single-output and linear, operating in continuous time.

Chapters 1 to 7 are intended as the core for a one-semester senior course; they would need supplementing with additional examples. These chapters constitute a basic treatment of feedback design, containing a detailed formulation of the control design problem, the fundamental issue of performance/stability robustness tradeoff, and the graphical design technique of loopshaping, suitable for benign plants (stable, minimum phase). Chapters 8 to 12 are more advanced and are intended for a first graduate course. Chapter 8 is a bridge to the latter half of the book, extending the loopshaping technique and connecting it with notions of optimality. Chapters 9 to 12 treat controller design via optimization. The approach in these latter chapters is mathematical rather than graphical, using elementary tools involving interpolation by analytic functions. This mathematical approach is most useful for multivariable systems, where graphical techniques usually break down. Nevertheless, we believe the setting of single-input/single-output systems is where this new approach should be learned.

There are many people to whom we are grateful for their help in this book: Dale Enns for sharing his expertise in loopshaping; Raymond Kwong and Boyd Pearson for class testing the book; and Munther Dahleh, Ciprian Foias, and Karen Rudie for reading earlier drafts. Numerous

Caltech students also struggled with various versions of this material: Gary Balas, Carolyn Beck, Bobby Bodenheimer, and Roy Smith had particularly helpful suggestions. Finally, we would like to thank the AFOSR, ARO, NSERC, NSF, and ONR for partial financial support during the writing of this book.

# Chapter 1

# Introduction

Without control systems there could be no manufacturing, no vehicles, no computers, no regulated environment—in short, no technology. Control systems are what make machines, in the broadest sense of the term, function as intended. Control systems are most often based on the principle of feedback, whereby the signal to be controlled is compared to a desired reference signal and the discrepancy used to compute corrective control action. The goal of this book is to present a theory of feedback control system design that captures the essential issues, can be applied to a wide range of practical problems, and is as simple as possible.

## 1.1 Issues in Control System Design

The process of designing a control system generally involves many steps. A typical scenario is as follows:

1. Study the system to be controlled and decide what types of sensors and actuators will be used and where they will be placed.
2. Model the resulting system to be controlled.
3. Simplify the model if necessary so that it is tractable.
4. Analyze the resulting model; determine its properties.
5. Decide on performance specifications.
6. Decide on the type of controller to be used.
7. Design a controller to meet the specs, if possible; if not, modify the specs or generalize the type of controller sought.
8. Simulate the resulting controlled system, either on a computer or in a pilot plant.
9. Repeat from step 1 if necessary.
10. Choose hardware and software and implement the controller.
11. Tune the controller on-line if necessary.

It must be kept in mind that a control engineer's role is not merely one of designing control systems for fixed plants, of simply "wrapping a little feedback" around an already fixed physical system. It also involves assisting in the choice and configuration of hardware by taking a system-wide view of performance. For this reason it is important that a theory of feedback not only lead to good designs when these are possible, but also indicate directly and unambiguously when the performance objectives cannot be met.

It is also important to realize at the outset that practical problems have uncertain, non-minimum-phase plants (*non-minimum-phase* means the existence of right half-plane zeros, so the inverse is unstable); that there are inevitably unmodeled dynamics that produce substantial uncertainty, usually at high frequency; and that sensor noise and input signal level constraints limit the achievable benefits of feedback. A theory that excludes some of these practical issues can still be useful in limited application domains. For example, many process control problems are so dominated by plant uncertainty and right half-plane zeros that sensor noise and input signal level constraints can be neglected. Some spacecraft problems, on the other hand, are so dominated by tradeoffs between sensor noise, disturbance rejection, and input signal level (e.g., fuel consumption) that plant uncertainty and non-minimum-phase effects are negligible. Nevertheless, any general theory should be able to treat all these issues explicitly and give quantitative and qualitative results about their impact on system performance.

In the present section we look at two issues involved in the design process: deciding on performance specifications and modeling. We begin with an example to illustrate these two issues.

**Example** A very interesting engineering system is the Keck astronomical telescope, currently under construction on Mauna Kea in Hawaii. When completed it will be the world's largest. The basic objective of the telescope is to collect and focus starlight using a large concave mirror. The shape of the mirror determines the quality of the observed image. The larger the mirror, the more light that can be collected, and hence the dimmer the star that can be observed. The diameter of the mirror on the Keck telescope will be 10 m. To make such a large, high-precision mirror out of a single piece of glass would be very difficult and costly. Instead, the mirror on the Keck telescope will be a mosaic of 36 hexagonal small mirrors. These 36 segments must then be aligned so that the composite mirror has the desired shape.

The control system to do this is illustrated in Figure 1.1. As shown, the mirror segments are subject to two types of forces: disturbance forces (described below) and forces from actuators. Behind each segment are three piston-type actuators, applying forces at three points on the segment to effect its orientation. In controlling the mirror's shape, it suffices to control the misalignment between adjacent mirror segments. In the gap between every two adjacent segments are (capacitor-type) sensors measuring local displacements between the two segments. These local displacements are stacked into the vector labeled $y$; this is what is to be controlled. For the mirror to have the ideal shape, these displacements should have certain ideal values that can be pre-computed; these are the components of the vector $r$. The controller must be designed so that in the closed-loop system $y$ is held close to $r$ despite the disturbance forces. Notice that the signals are vector valued. Such a system is *multivariable*.

Our uncertainty about the plant arises from disturbance sources:

- As the telescope turns to track a star, the direction of the force of gravity on the mirror changes.
- During the night, when astronomical observations are made, the ambient temperature changes.
- The telescope is susceptible to wind gusts.

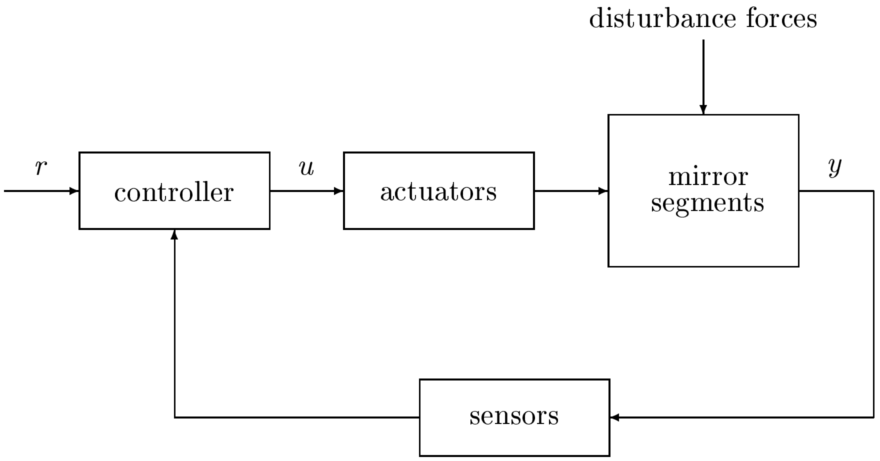


Figure 1.1: Block diagram of Keck telescope control system.

and from uncertain plant dynamics:

- The dynamic behavior of the components—mirror segments, actuators, sensors—cannot be modeled with infinite precision.

Now we continue with a discussion of the issues in general.

### Control Objectives

Generally speaking, the objective in a control system is to make some output, say $y$, behave in a desired way by manipulating some input, say $u$. The simplest objective might be to keep $y$ small (or close to some equilibrium point)—a *regulator problem*—or to keep $y - r$ small for $r$, a reference or command signal, in some set—a *servomechanism* or *servo problem*. Examples:

- On a commercial airplane the vertical acceleration should be less than a certain value for passenger comfort.
- In an audio amplifier the power of noise signals at the output must be sufficiently small for high fidelity.
- In papermaking the moisture content must be kept between prescribed values.

There might be the side constraint of keeping $u$ itself small as well, because it might be constrained (e.g., the flow rate from a valve has a maximum value, determined when the valve is fully open) or it might be too expensive to use a large input. But what is small for a signal? It is natural to introduce norms for signals; then "$y$ small" means "$\|y\|$ small." Which norm is appropriate depends on the particular application.

In summary, performance objectives of a control system naturally lead to the introduction of norms; then the specs are given as norm bounds on certain key signals of interest.

### Models

Before discussing the issue of modeling a physical system it is important to distinguish among four different objects:

1. *Real physical system*: the one "out there."
2. *Ideal physical model*: obtained by schematically decomposing the real physical system into ideal building blocks; composed of resistors, masses, beams, kilns, isotropic media, Newtonian fluids, electrons, and so on.
3. *Ideal mathematical model*: obtained by applying natural laws to the ideal physical model; composed of nonlinear partial differential equations, and so on.
4. *Reduced mathematical model*: obtained from the ideal mathematical model by linearization, lumping, and so on; usually a rational transfer function.

Sometimes language makes a fuzzy distinction between the real physical system and the ideal physical model. For example, the word *resistor* applies to both the actual piece of ceramic and metal and the ideal object satisfying Ohm's law. Of course, the adjectives *real* and *ideal* could be used to disambiguate.

No mathematical system can precisely model a real physical system; there is always uncertainty. Uncertainty means that we cannot predict exactly what the output of a real physical system will be even if we know the input, so *we* are uncertain about the system. Uncertainty arises from two sources: unknown or unpredictable inputs (disturbance, noise, etc.) and unpredictable dynamics.

What should a model provide? It should predict the input-output response in such a way that we can use it to design a control system, and then be confident that the resulting design will work on the real physical system. Of course, this is not possible. A "leap of faith" will always be required on the part of the engineer. This cannot be eliminated, but it can be made more manageable with the use of effective modeling, analysis, and design techniques.

### Mathematical Models in This Book

The models in this book are finite-dimensional, linear, and time-invariant. The main reason for this is that they are the simplest models for treating the fundamental issues in control system design. The resulting design techniques work remarkably well for a large class of engineering problems, partly because most systems are built to be as close to linear time-invariant as possible so that they are more easily controlled. Also, a good controller will keep the system in its linear regime. The uncertainty description is as simple as possible as well.

The basic form of the plant model in this book is

$$y = (P + \Delta)u + n.$$

Here $y$ is the output, $u$ the input, and $P$ the nominal plant transfer function. The model uncertainty comes in two forms:

$n$: unknown noise or disturbance
$\Delta$: unknown plant perturbation

Both $n$ and $\Delta$ will be assumed to belong to sets, that is, some *a priori* information is assumed about $n$ and $\Delta$. Then every input $u$ is capable of producing a *set* of outputs, namely, the set of all outputs $(P + \Delta)u + n$ as $n$ and $\Delta$ range over their sets. Models capable of producing sets of outputs for a single input are said to be *nondeterministic*. There are two main ways of obtaining models, as described next.

### Models from Science

The usual way of getting a model is by applying the laws of physics, chemistry, and so on. Consider the Keck telescope example. One can write down differential equations based on physical principles (e.g., Newton's laws) and making idealizing assumptions (e.g., the mirror segments are rigid). The coefficients in the differential equations will depend on physical constants, such as masses and physical dimensions. These can be measured. This method of applying physical laws and taking measurements is most successful in electromechanical systems, such as aerospace vehicles and robots. Some systems are difficult to model in this way, either because they are too complex or because their governing laws are unknown.

### Models from Experimental Data

The second way of getting a model is by doing experiments on the physical system. Let's start with a simple thought experiment, one that captures many essential aspects of the relationships between physical systems and their models and the issues in obtaining models from experimental data. Consider a real physical system—the plant to be controlled—with one input, $u$, and one output, $y$. To design a control system for this plant, we must understand how $u$ affects $y$.

The experiment runs like this. Suppose that the real physical system is in a rest state before an input $u$ is applied (i.e., $u = y = 0$). Now apply some input signal $u$, resulting in some output signal $y$. Observe the pair $(u, y)$. Repeat this experiment several times. Pretend that these data pairs are all we know about the real physical system. (This is the *black box* scenario. Usually, we know something about the internal workings of the system.)

After doing this experiment we will notice several things. First, the same input signal at different times produces different output signals. Second, if we hold $u = 0$, $y$ will fluctuate in an unpredictable manner. Thus the real physical system produces just one output for any given input, so it itself is deterministic. However, we observers are uncertain because we cannot predict what that output will be.

Ideally, the model should *cover* the data in the sense that it should be capable of producing every experimentally observed input-output pair. (Of course, it would be better to cover not just the data observed in a finite number of experiments, but anything that can be produced by the real physical system. Obviously, this is impossible.) If nondeterminism that reasonably covers the range of expected data is not built into the model, we will not trust that designs based on such models will work on the real system.

In summary, for a useful theory of control design, plant models must be nondeterministic, having uncertainty built in explicitly.

### Synthesis Problem

A synthesis problem is a theoretical problem, precise and unambiguous. Its purpose is primarily pedagogical: It gives us something clear to focus on for the purpose of study. The hope is that the principles learned from studying a formal synthesis problem will be useful when it comes to designing a real control system.

The most general block diagram of a control system is shown in Figure 1.2. The generalized plant consists of everything that is fixed at the start of the control design exercise: the plant, actuators that generate inputs to the plant, sensors measuring certain signals, analog-to-digital and digital-to-analog converters, and so on. The controller consists of the designable part: it may be an electric circuit, a programmable logic controller, a general-purpose computer, or some other

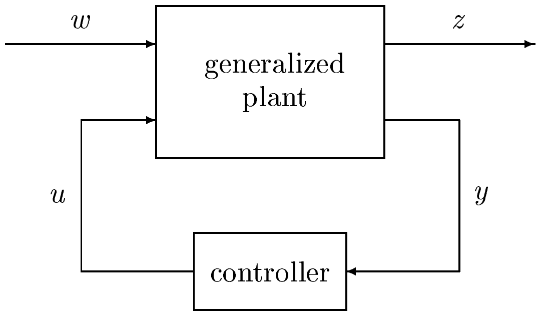


Figure 1.2: Most general control system.

such device. The signals $w$, $z$, $y$, and $u$ are, in general, vector-valued functions of time. The components of $w$ are all the exogenous inputs: references, disturbances, sensor noises, and so on. The components of $z$ are all the signals we wish to control: tracking errors between reference signals and plant outputs, actuator signals whose values must be kept between certain limits, and so on. The vector $y$ contains the outputs of all sensors. Finally, $u$ contains all controlled inputs to the generalized plant. (Even open-loop control fits in; the generalized plant would be so defined that $y$ is always constant.)

Very rarely is the exogenous input $w$ a fixed, known signal. One of these rare instances is where a robot manipulator is required to trace out a definite path, as in welding. Usually, $w$ is not fixed but belongs to a set that can be characterized to some degree. Some examples:

- In a thermostat-controlled temperature regulator for a house, the reference signal is always piecewise constant: at certain times during the day the thermostat is set to a new value. The temperature of the outside air is not piecewise constant but varies slowly within bounds.
- In a vehicle such as an airplane or ship the pilot's commands on the steering wheel, throttle, pedals, and so on come from a predictable set, and the gusts and wave motions have amplitudes and frequencies that can be bounded with some degree of confidence.
- The load power drawn on an electric power system has predictable characteristics.

Sometimes the designer does not attempt to model the exogenous inputs. Instead, she or he designs for a suitable response to a test input, such as a step, a sinusoid, or white noise. The designer may know from past experience how this correlates with actual performance in the field. Desired properties of $z$ generally relate to how large it is according to various measures, as discussed above.

Finally, the output of the design exercise is a mathematical model of a controller. This must be implementable in hardware. If the controller you design is governed by a nonlinear partial differential equation, how are you going to implement it? A linear ordinary differential equation with constant coefficients, representing a finite-dimensional, time-invariant, linear system, can be simulated via an analog circuit or approximated by a digital computer, so this is the most common type of control law.

The synthesis problem can now be stated as follows: Given a set of generalized plants, a set of exogenous inputs, and an upper bound on the size of $z$, design an implementable controller to

achieve this bound. How the size of $z$ is to be measured (e.g., power or maximum amplitude) depends on the context. This book focuses on an elementary version of this problem.

## 1.2 What Is in This Book

Since this book is for a first course on this subject, attention is restricted to systems whose models are single-input/single-output, finite-dimensional, linear, and time-invariant. Thus they have transfer functions that are rational in the Laplace variable $s$. The general layout of the book is that Chapters 2 to 4 and 6 are devoted to analysis of control systems, that is, the controller is already specified, and Chapters 5 and 7 to 12 to design.

Performance of a control system is specified in terms of the size of certain signals of interest. For example, the performance of a tracking system could be measured by the size of the error signal. Chapter 2, *Norms for Signals and Systems*, looks at several ways of defining norms for a signal $u(t)$; in particular, the 2-norm (associated with energy),

$$\left(\int_{-\infty}^{\infty} u(t)^2 dt\right)^{1/2},$$

the $\infty$-norm (maximum absolute value),

$$\max_t |u(t)|,$$

and the square root of the average power (actually, not quite a norm),

$$\left(\lim_{T\to\infty} \frac{1}{2T}\int_{-T}^{T} u(t)^2 dt\right)^{1/2}.$$

Also introduced are two norms for a system's transfer function $G(s)$: the 2-norm,

$$\|G\|_2 := \left(\frac{1}{2\pi}\int_{-\infty}^{\infty} |G(j\omega)|^2 d\omega\right)^{1/2},$$

and the $\infty$-norm,

$$\|G\|_\infty := \max_\omega |G(j\omega)|.$$

Notice that $\|G\|_\infty$ equals the peak amplitude on the Bode magnitude plot of $G$. Then two very useful tables are presented summarizing input-output norm relationships. For example, one table gives a bound on the 2-norm of the output knowing the 2-norm of the input and the $\infty$-norm of the transfer function. Such results are very useful in predicting, for example, the effect a disturbance will have on the output of a feedback system.

Chapters 3 and 4 are the most fundamental in the book. The system under consideration is shown in Figure 1.3, where $P$ and $C$ are the plant and controller transfer functions. The signals are as follows:

| | |
|---|---|
| $r$ | reference or command input |
| $e$ | tracking error |
| $u$ | control signal, controller output |
| $d$ | plant disturbance |
| $y$ | plant output |
| $n$ | sensor noise |

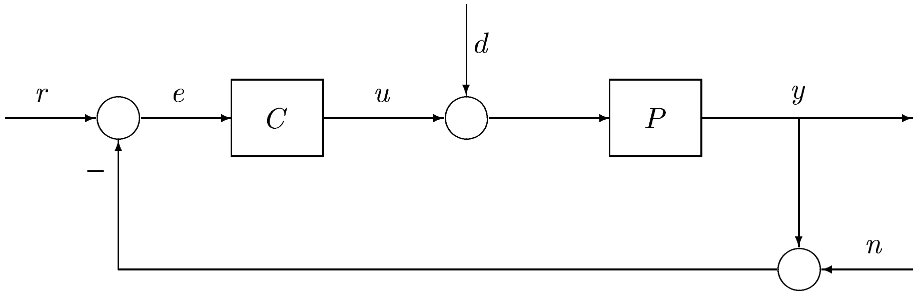


Figure 1.3: Single-loop feedback system.

In Chapter 3, *Basic Concepts*, internal stability is defined and characterized. Then the system is analyzed for its ability to track a *single* reference signal $r$—a step or a ramp—asymptotically as time increases. Finally, we look at tracking a *set* of reference signals. The transfer function from reference input $r$ to tracking error $e$ is denoted $S$, the *sensitivity function.* It is argued that a useful tracking performance criterion is $\|W_1 S\|_\infty < 1$, where $W_1$ is a transfer function which can be tuned by the control system designer.

Since no mathematical system can exactly model a physical system, we must be aware of how modeling errors might adversely affect the performance of a control system. Chapter 4, *Uncertainty and Robustness*, begins with a treatment of various models of plant uncertainty. The basic technique is to model the plant as belonging to a set $\mathcal{P}$. Such a set can be either *structured*—for example, there are a finite number of uncertain parameters—or *unstructured*—the frequency response lies in a set in the complex plane for every frequency. For us, unstructured is more important because it leads to a simple and useful design theory. In particular, multiplicative perturbation is chosen for detailed study, it being typical. In this uncertainty model there is a nominal plant $P$ and the family $\mathcal{P}$ consists of all perturbed plants $\tilde{P}$ such that at each frequency $\omega$ the ratio $\tilde{P}(j\omega)/P(j\omega)$ lies in a disk in the complex plane with center 1. This notion of disk-like uncertainty is key; because of it the mathematical problems are tractable.

Generally speaking, the notion of robustness means that some characteristic of the feedback system holds for every plant in the set $\mathcal{P}$. A controller $C$ provides *robust stability* if it provides internal stability for every plant in $\mathcal{P}$. Chapter 4 develops a test for robust stability for the multiplicative perturbation model, a test involving $C$ and $\mathcal{P}$. The test is $\|W_2 T\|_\infty < 1$. Here $T$ is the *complementary sensitivity function*, equal to $1 - S$ (or the transfer function from $r$ to $y$), and $W_2$ is a transfer function whose magnitude at frequency $\omega$ equals the radius of the uncertainty disk at that frequency.

The final topic in Chapter 4 is robust performance, guaranteed tracking in the face of plant uncertainty. The main result is that the tracking performance spec $\|W_1 S\|_\infty < 1$ is satisfied for all plants in the multiplicative perturbation set if and only if the magnitude of $|W_1 S| + |W_2 T|$ is less than 1 for all frequencies, that is,

$$\| |W_1 S| + |W_2 T| \|_\infty < 1. \tag{1.1}$$

This is an analysis result: It tells exactly when some candidate controller provides robust performance.

Chapter 5, *Stabilization*, is the first on design. Most synthesis problems can be formulated like this: Given $P$, design $C$ so that the feedback system (1) is internally stable, and (2) acquires some

additional desired property or properties, for example, the output $y$ asymptotically tracks a step input $r$. The method of solution presented here is to parametrize all $C$s for which (1) is true and then to find a parameter for which (2) holds. In this chapter such a parametrization is derived; it has the form

$$C = \frac{X + MQ}{Y - NQ},$$

where $N$, $M$, $X$, and $Y$ are fixed stable proper transfer functions and $Q$ is the parameter, an arbitrary stable proper transfer function. The usefulness of this parametrization derives from the fact that all closed-loop transfer functions are very simple functions of $Q$; for instance, the sensitivity function $S$, while a nonlinear function of $C$, equals simply $MY - MNQ$. This parametrization is then applied to three problems: achieving asymptotic performance specs, such as tracking a step; internal stabilization by a stable controller; and simultaneous stabilization of two plants by a common controller.

Before we see how to design control systems for the robust performance specification, it is important to understand the basic limitations on achievable performance: Why can't we achieve both arbitrarily good performance and stability robustness at the same time? In Chapter 6, *Design Constraints*, we study design constraints arising from two sources: from algebraic relationships that must hold among various transfer functions and from the fact that closed-loop transfer functions must be stable, that is, analytic in the right half-plane. The main conclusion is that feedback control design always involves a tradeoff between performance and stability robustness.

Chapter 7, *Loopshaping*, presents a graphical technique for designing a controller to achieve robust performance. This method is the most common in engineering practice. It is especially suitable for today's CAD packages in view of their graphics capabilities. The loop transfer function is $L := PC$. The idea is to shape the Bode magnitude plot of $L$ so that (1.1) is achieved, at least approximately, and then to back-solve for $C$ via $C = L/P$. When $P$ or $P^{-1}$ is not stable, $L$ must contain $P$s unstable poles and zeros (for internal stability of the feedback loop), an awkward constraint. For this reason, it is assumed in Chapter 7 that $P$ and $P^{-1}$ are both stable.

Thus Chapters 2 to 7 constitute a basic treatment of feedback design, containing a detailed formulation of the control design problem, the fundamental issue of performance/stability robustness tradeoff, and a graphical design technique suitable for benign plants (stable, minimum-phase). Chapters 8 to 12 are more advanced.

Chapter 8, *Advanced Loopshaping*, is a bridge between the two halves of the book; it extends the loopshaping technique and connects it with the notion of optimal designs. Loopshaping in Chapter 7 focuses on $L$, but other quantities, such as $C$, $S$, $T$, or the $Q$ parameter in the stabilization results of Chapter 5, may also be "shaped" to achieve the same end. For many problems these alternatives are more convenient. Chapter 8 also offers some suggestions on how to extend loopshaping to handle right half-plane poles and zeros.

Optimal controllers are introduced in a formal way in Chapter 8. Several different notions of optimality are considered with an aim toward understanding in what way loopshaping controllers can be said to be optimal. It is shown that loopshaping controllers satisfy a very strong type of optimality, called *self-optimality*. The implication of this result is that when loopshaping is successful at finding an adequate controller, it cannot be improved upon uniformly.

Chapters 9 to 12 present a recently developed approach to the robust performance design problem. The approach is mathematical rather than graphical, using elementary tools involving interpolation by analytic functions. This mathematical approach is most useful for multivariable systems, where graphical techniques usually break down. Nevertheless, the setting of single-input/single-output systems is where this new approach should be learned. Besides, present-day software for

control design (e.g., MATLAB and Program CC) incorporate this approach.

Chapter 9, *Model Matching*, studies a hypothetical control problem called the model-matching problem: Given stable proper transfer functions $T_1$ and $T_2$, find a stable transfer function $Q$ to minimize $\|T_1 - T_2Q\|_\infty$. The interpretation is this: $T_1$ is a model, $T_2$ is a plant, and $Q$ is a cascade controller to be designed so that $T_2Q$ approximates $T_1$. Thus $T_1 - T_2Q$ is the error transfer function. This problem is turned into a special interpolation problem: Given points $\{a_i\}$ in the right half-plane and values $\{b_i\}$, also complex numbers, find a stable transfer function $G$ so that $\|G\|_\infty < 1$ and $G(a_i) = b_i$, that is, $G$ interpolates the value $b_i$ at the point $a_i$. When such a $G$ exists and how to find one utilizes some beautiful mathematics due to Nevanlinna and Pick.

Chapter 10, *Design for Performance*, treats the problem of designing a controller to achieve the performance criterion $\|W_1S\|_\infty < 1$ alone, that is, with no plant uncertainty. When does such a controller exist, and how can it be computed? These questions are easy when the inverse of the plant transfer function is stable. When the inverse is unstable (i.e., $P$ is non-minimum-phase), the questions are more interesting. The solutions presented in this chapter use model-matching theory. The procedure is applied to designing a controller for a flexible beam. The desired performance is given in terms of step response specs: overshoot and settling time. It is shown how to choose the weight $W_1$ to accommodate these time domain specs. Also treated in Chapter 10 is minimization of the 2-norm of some closed-loop transfer function, e.g., $\|W_1S\|_2$.

Next, in Chapter 11, *Stability Margin Optimization*, is considered the problem of designing a controller whose sole purpose is to maximize the stability margin, that is, performance is ignored. The maximum obtainable stability margin is a measure of how difficult the plant is to control. Three measures of stability margin are treated: the $\infty$-norm of a multiplicative perturbation, gain margin, and phase margin. It is shown that the problem of optimizing these stability margins can also be reduced to a model-matching problem.

Chapter 12, *Design for Robust Performance*, returns to the robust performance problem of designing a controller to achieve (1.1). Chapter 7 proposed loopshaping as a graphical method when $P$ and $P^{-1}$ are stable. Without these assumptions loopshaping can be awkward and the methodical procedure in this chapter can be used. Actually, (1.1) is too hard for mathematical analysis, so a compromise criterion is posed, namely,

$$\||W_1S|^2 + |W_2T|^2\|_\infty < 1/2. \tag{1.2}$$

Using a technique called spectral factorization, we can reduce this problem to a model-matching problem. As an illustration, the flexible beam example is reconsidered; besides step response specs on the tip deflection, a hard limit is placed on the plant input to prevent saturation of an amplifier.

Finally, some words about frequency-domain versus time-domain methods of design. Horowitz (1963) has long maintained that "frequency response methods have been found to be especially useful and transparent, enabling the designer to see the tradeoff between conflicting design factors." This point of view has gained much greater acceptance within the control community at large in recent years, although perhaps it would be better to stress the importance of input-output or operator-theoretic versus state-space methods, instead of frequency domain versus time domain. This book focuses almost exclusively on input-output methods, not because they are ultimately more fundamental than state-space methods, but simply for pedagogical reasons.

## Notes and References

There are many books on feedback control systems. Particularly good ones are Bower and Schultheiss (1961) and Franklin et al. (1986). Regarding the Keck telescope, see Aubrun et al. (1987, 1988).

# Chapter 2

# Norms for Signals and Systems

One way to describe the performance of a control system is in terms of the size of certain signals of interest. For example, the performance of a tracking system could be measured by the size of the error signal. This chapter looks at several ways of defining a signal's size (i.e., at several norms for signals). Which norm is appropriate depends on the situation at hand. Also introduced are norms for a system's transfer function. Then two very useful tables are developed summarizing input-output norm relationships.

## 2.1 Norms for Signals

We consider signals mapping $(-\infty, \infty)$ to $\mathbb{R}$. They are assumed to be piecewise continuous. Of course, a signal may be zero for $t < 0$ (i.e., it may start at time $t = 0$).

We are going to introduce several different norms for such signals. First, recall that a norm must have the following four properties:

(i) $\|u\| \geq 0$

(ii) $\|u\| = 0 \Leftrightarrow u(t) = 0, \qquad \forall t$

(iii) $\|au\| = |a|\|u\|, \qquad \forall a \in \mathbb{R}$

(iv) $\|u + v\| \leq \|u\| + \|v\|$

The last property is the familiar triangle inequality.

**1-Norm** The 1-norm of a signal $u(t)$ is the integral of its absolute value:

$$\|u\|_1 := \int_{-\infty}^{\infty} |u(t)| dt.$$

**2-Norm** The 2-norm of $u(t)$ is

$$\|u\|_2 := \left( \int_{-\infty}^{\infty} u(t)^2 dt \right)^{1/2}.$$

For example, suppose that $u$ is the current through a 1 $\Omega$ resistor. Then the instantaneous power equals $u(t)^2$ and the total energy equals the integral of this, namely, $\|u\|_2^2$. We shall generalize this

interpretation: The *instantaneous power* of a signal $u(t)$ is defined to be $u(t)^2$ and its *energy* is defined to be the square of its 2-norm.

**$\infty$-Norm** The $\infty$-norm of a signal is the least upper bound of its absolute value:

$$\|u\|_\infty := \sup_t |u(t)|.$$

For example, the $\infty$-norm of

$$(1 - \mathrm{e}^{-t})1(t)$$

equals 1. Here $1(t)$ denotes the unit step function.

**Power Signals** The *average power* of $u$ is the average over time of its instantaneous power:

$$\lim_{T\to\infty} \frac{1}{2T}\int_{-T}^{T} u(t)^2 dt.$$

The signal $u$ will be called a *power signal* if this limit exists, and then the squareroot of the average power will be denoted $pow(u)$:

$$pow(u) := \left(\lim_{T\to\infty} \frac{1}{2T}\int_{-T}^{T} u(t)^2 dt\right)^{1/2}.$$

Note that a nonzero signal can have zero average power, so *pow* is not a norm. It does, however, have properties (i), (iii), and (iv).

Now we ask the question: Does finiteness of one norm imply finiteness of any others? There are some easy answers:

1. If $\|u\|_2 < \infty$, then $u$ is a power signal with $pow(u) = 0$.

   **Proof** Assuming that $u$ has finite 2-norm, we get

   $$\frac{1}{2T}\int_{-T}^{T} u(t)^2 dt \le \frac{1}{2T}\|u\|_2^2.$$

   But the right-hand side tends to zero as $T \to \infty$. ■

2. If $u$ is a power signal and $\|u\|_\infty < \infty$, then $pow(u) \le \|u\|_\infty$.

   **Proof** We have

   $$\frac{1}{2T}\int_{-T}^{T} u(t)^2 dt \le \|u\|_\infty^2 \frac{1}{2T}\int_{-T}^{T} dt = \|u\|_\infty^2.$$

   Let $T$ tend to $\infty$. ■

3. If $\|u\|_1 < \infty$ and $\|u\|_\infty < \infty$, then $\|u\|_2 \le (\|u\|_\infty \|u\|_1)^{1/2}$, and hence $\|u\|_2 < \infty$.

   **Proof**

   $$\int_{-\infty}^{\infty} u(t)^2 dt = \int_{-\infty}^{\infty} |u(t)||u(t)| dt \le \|u\|_\infty \|u\|_1 \quad ■$$

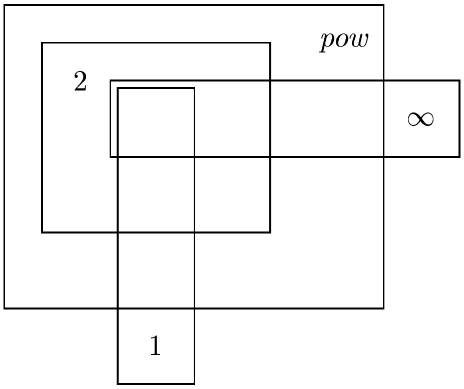


Figure 2.1: Set inclusions.

A Venn diagram summarizing the set inclusions is shown in Figure 2.1. Note that the set labeled "*pow*" contains all power signals for which *pow* is finite; the set labeled "1" contains all signals of finite 1-norm; and so on. It is instructive to get examples of functions in all the components of this diagram (Exercise 2). For example, consider

$$u_1(t) = \begin{cases} 0, & \text{if } t \le 0 \\ 1/\sqrt{t}, & \text{if } 0 < t \le 1 \\ 0, & \text{if } t > 1. \end{cases}$$

This has finite 1-norm:

$$\|u_1\|_1 = \int_0^1 \frac{1}{\sqrt{t}} dt = 2.$$

Its 2-norm is infinite because the integral of $1/t$ is divergent over the interval $[0,1]$. For the same reason, $u_1$ is not a power signal. Finally, $u_1$ is not bounded, so $\|u_1\|_\infty$ is infinite. Therefore, $u_1$ lives in the bottom component in the diagram.

## 2.2 Norms for Systems

We consider systems that are linear, time-invariant, causal, and (usually) finite-dimensional. In the time domain an input-output model for such a system has the form of a convolution equation,

$$y = G * u,$$

that is,

$$y(t) = \int_{-\infty}^{\infty} G(t-\tau)u(\tau)d\tau.$$

Causality means that $G(t) = 0$ for $t < 0$. Let $\hat{G}(s)$ denote the transfer function, the Laplace transform of $G$. Then $\hat{G}$ is rational (by finite-dimensionality) with real coefficients. We say that $\hat{G}$ is *stable* if it is analytic in the closed right half-plane (Re $s \ge 0$), *proper* if $\hat{G}(j\infty)$ is finite (degree of denominator $\ge$ degree of numerator), *strictly proper* if $\hat{G}(j\infty) = 0$ (degree of denominator $>$ degree of numerator), and *biproper* if $\hat{G}$ and $\hat{G}^{-1}$ are both proper (degree of denominator = degree of numerator).

We introduce two norms for the transfer function $\hat{G}$.

**2-Norm**

$$\|\hat{G}\|_2 := \left(\frac{1}{2\pi}\int_{-\infty}^{\infty}|\hat{G}(j\omega)|^2 d\omega\right)^{1/2}$$

**$\infty$-Norm**

$$\|\hat{G}\|_\infty := \sup_\omega |\hat{G}(j\omega)|$$

Note that if $\hat{G}$ is stable, then by Parseval's theorem

$$\|\hat{G}\|_2 = \left(\frac{1}{2\pi}\int_{-\infty}^{\infty}|\hat{G}(j\omega)|^2 d\omega\right)^{1/2} = \left(\int_{-\infty}^{\infty}|G(t)|^2 dt\right)^{1/2}.$$

The $\infty$-norm of $\hat{G}$ equals the distance in the complex plane from the origin to the farthest point on the Nyquist plot of $\hat{G}$. It also appears as the peak value on the Bode magnitude plot of $\hat{G}$. An important property of the $\infty$-norm is that it is submultiplicative:

$$\|\hat{G}\hat{H}\|_\infty \le \|\hat{G}\|_\infty \|\hat{H}\|_\infty.$$

It is easy to tell when these two norms are finite.

**Lemma 1** *The 2-norm of $\hat{G}$ is finite iff $\hat{G}$ is strictly proper and has no poles on the imaginary axis; the $\infty$-norm is finite iff $\hat{G}$ is proper and has no poles on the imaginary axis.*

**Proof** Assume that $\hat{G}$ is strictly proper, with no poles on the imaginary axis. Then the Bode magnitude plot rolls off at high frequency. It is not hard to see that the plot of $c/(\tau s+1)$ dominates that of $\hat{G}$ for sufficiently large positive $c$ and sufficiently small positive $\tau$, that is,

$$|c/(\tau j\omega + 1)| \ge |\hat{G}(j\omega)|, \qquad \forall \omega.$$

But $c/(\tau s + 1)$ has finite 2-norm; its 2-norm equals $c/\sqrt{2\tau}$ (how to do this computation is shown below). Hence $\hat{G}$ has finite 2-norm.

The rest of the proof follows similar lines. ■

**How to Compute the 2-Norm**

Suppose that $\hat{G}$ is strictly proper and has no poles on the imaginary axis (so its 2-norm is finite). We have

$$\begin{aligned}\|\hat{G}\|_2^2 &= \frac{1}{2\pi}\int_{-\infty}^{\infty}|\hat{G}(j\omega)|^2 d\omega \\ &= \frac{1}{2\pi j}\int_{-j\infty}^{j\infty}\hat{G}(-s)\hat{G}(s)ds \\ &= \frac{1}{2\pi j}\oint \hat{G}(-s)\hat{G}(s)ds.\end{aligned}$$

The last integral is a contour integral up the imaginary axis, then around an infinite semicircle in the left half-plane; the contribution to the integral from this semicircle equals zero because $\hat{G}$ is

strictly proper. By the residue theorem, $\|\hat{G}\|_2^2$ equals the sum of the residues of $\hat{G}(-s)\hat{G}(s)$ at its poles in the left half-plane.

**Example 1** Take $\hat{G}(s) = 1/(\tau s+1)$, $\tau > 0$. The left half-plane pole of $\hat{G}(-s)\hat{G}(s)$ is at $s = -1/\tau$. The residue at this pole equals

$$\lim_{s \to -1/\tau} \left(s + \frac{1}{\tau}\right) \frac{1}{-\tau s + 1} \frac{1}{\tau s + 1} = \frac{1}{2\tau}.$$

Hence $\|\hat{G}\|_2 = 1/\sqrt{2\tau}$.

**How to Compute the $\infty$-Norm**

This requires a search. Set up a fine grid of frequency points,

$$\{\omega_1, \ldots, \omega_N\}.$$

Then an estimate for $\|\hat{G}\|_\infty$ is

$$\max_{1 \le k \le N} |\hat{G}(j\omega_k)|.$$

Alternatively, one could find where $|\hat{G}(j\omega)|$ is maximum by solving the equation

$$\frac{d|\hat{G}|^2}{d\omega}(j\omega) = 0.$$

This derivative can be computed in closed form because $\hat{G}$ is rational. It then remains to compute the roots of a polynomial.

**Example 2** Consider

$$\hat{G}(s) = \frac{as+1}{bs+1}$$

with $a, b > 0$. Look at the Bode magnitude plot: For $a \ge b$ it is increasing (high-pass); else, it is decreasing (low-pass). Thus

$$\|\hat{G}\|_\infty = \begin{cases} a/b, & a \ge b \\ 1, & a < b. \end{cases}$$

## 2.3 Input-Output Relationships

The question of interest in this section is: If we know how big the input is, how big is the output going to be? Consider a linear system with input $u$, output $y$, and transfer function $\hat{G}$, assumed stable and strictly proper. The results are summarized in two tables below. Suppose that $u$ is the unit impulse, $\delta$. Then the 2-norm of $y$ equals the 2-norm of $G$, which by Parseval's theorem equals the 2-norm of $\hat{G}$; this gives entry (1,1) in Table 2.1. The rest of the first column is for the $\infty$-norm and *pow*, and the second column is for a sinusoidal input. The $\infty$ in the (1,2) entry is true as long as $\hat{G}(j\omega) \neq 0$.

| | $u(t) = \delta(t)$ | $u(t) = \sin(\omega t)$ |
|---|---|---|
| $\|y\|_2$ | $\|\hat{G}\|_2$ | $\infty$ |
| $\|y\|_\infty$ | $\|G\|_\infty$ | $\|\hat{G}(j\omega)\|$ |
| $pow(y)$ | 0 | $\frac{1}{\sqrt{2}}\|\hat{G}(j\omega)\|$ |

Table 2.1: Output norms and *pow* for two inputs

Now suppose that $u$ is not a fixed signal but that it can be any signal of 2-norm $\leq 1$. It turns out that the least upper bound on the 2-norm of the output, that is,

$$\sup\{\|y\|_2 : \|u\|_2 \leq 1\},$$

which we can call the 2-norm/2-norm *system gain*, equals the $\infty$-norm of $\hat{G}$; this provides entry (1,1) in Table 2.2. The other entries are the other system gains. The $\infty$ in the various entries is true as long as $\hat{G} \not\equiv 0$, that is, as long as there is some $\omega$ for which $\hat{G}(j\omega) \neq 0$.

| | $\|u\|_2$ | $\|u\|_\infty$ | $pow(u)$ |
|---|---|---|---|
| $\|y\|_2$ | $\|\hat{G}\|_\infty$ | $\infty$ | $\infty$ |
| $\|y\|_\infty$ | $\|\hat{G}\|_2$ | $\|G\|_1$ | $\infty$ |
| $pow(y)$ | 0 | $\leq \|\hat{G}\|_\infty$ | $\|\hat{G}\|_\infty$ |

Table 2.2: System Gains

A typical application of these tables is as follows. Suppose that our control analysis or design problem involves, among other things, a requirement of disturbance attenuation: The controlled system has a disturbance input, say $u$, whose effect on the plant output, say $y$, should be small. Let $G$ denote the impulse response from $u$ to $y$. The controlled system will be required to be stable, so the transfer function $\hat{G}$ will be stable. Typically, it will be strictly proper, too (or at least proper). The tables tell us how much $u$ affects $y$ according to various measures. For example, if $u$ is known to be a sinusoid of fixed frequency (maybe $u$ comes from a power source at 60 Hz), then the second column of Table 2.1 gives the relative size of $y$ according to the three measures. More commonly, the disturbance signal will not be known *a priori*, so Table 2.2 will be more relevant.

Notice that the $\infty$-norm of the transfer function appears in several entries in the tables. This norm is therefore an important measure for system performance.

**Example** A system with transfer function $1/(10s+1)$ has a disturbance input $d(t)$ known to have the energy bound $\|d\|_2 \leq 0.4$. Suppose that we want to find the best estimate of the $\infty$-norm of the output $y(t)$. Table 2.2 says that the 2-norm/$\infty$-norm gain equals the 2-norm of the transfer function, which equals $1/\sqrt{20}$. Thus

$$\|y\|_\infty \leq \frac{0.4}{\sqrt{20}}.$$

The next two sections concern the proofs of the tables and are therefore optional.

## 2.4 Power Analysis (Optional)

For a power signal $u$ define the *autocorrelation function*

$$R_u(\tau) := \lim_{T\to\infty} \frac{1}{2T} \int_{-T}^{T} u(t)u(t+\tau)dt,$$

that is, $R_u(\tau)$ is the average value of the product $u(t)u(t+\tau)$. Observe that

$$R_u(0) = pow(u)^2 \geq 0.$$

We must restrict our definition of a power signal to those signals for which the above limit exists for all values of $\tau$, not just $\tau = 0$. For such signals we have the additional property that

$$|R_u(\tau)| \leq R_u(0).$$

**Proof** The Cauchy-Schwarz inequality implies that

$$\left|\int_{-T}^{T} u(t)v(t)dt\right| \leq \left(\int_{-T}^{T} u(t)^2 dt\right)^{1/2} \left(\int_{-T}^{T} v(t)^2 dt\right)^{1/2}.$$

Set $v(t) = u(t+\tau)$ and multiply by $1/(2T)$ to get

$$\left|\frac{1}{2T}\int_{-T}^{T} u(t)u(t+\tau)dt\right| \leq \left(\frac{1}{2T}\int_{-T}^{T} u(t)^2 dt\right)^{1/2} \left(\frac{1}{2T}\int_{-T}^{T} u(t+\tau)^2 dt\right)^{1/2}.$$

Now let $T \to \infty$ to get the desired result. ■

Let $S_u$ denote the Fourier transform of $R_u$. Thus

$$\begin{aligned} S_u(j\omega) &= \int_{-\infty}^{\infty} R_u(\tau)\mathrm{e}^{-j\omega\tau} d\tau, \\ R_u(\tau) &= \frac{1}{2\pi}\int_{-\infty}^{\infty} S_u(j\omega)\mathrm{e}^{j\omega\tau} d\omega, \\ pow(u)^2 &= R_u(0) = \frac{1}{2\pi}\int_{-\infty}^{\infty} S_u(j\omega) d\omega. \end{aligned}$$

From the last equation we interpret $S_u(j\omega)/2\pi$ as power density. The function $S_u$ is called the *power spectral density* of the signal $u$.

Now consider two power signals, $u$ and $v$. Their *cross-correlation function* is

$$R_{uv}(\tau) := \lim_{T\to\infty} \frac{1}{2T}\int_{-T}^{T} u(t)v(t+\tau)dt$$

and $S_{uv}$, the Fourier transform, is called their *cross-power spectral density function.*

We now derive some useful facts concerning a linear system with transfer function $\hat{G}$, assumed stable and proper, and its input $u$ and output $y$.

1. $R_{uy} = G * R_u$

**Proof** Since

$$y(t) = \int_{-\infty}^{\infty} G(\alpha)u(t-\alpha)d\alpha \tag{2.1}$$

we have

$$u(t)y(t+\tau) = \int_{-\infty}^{\infty} G(\alpha)u(t)u(t+\tau-\alpha)d\alpha.$$

Thus the average value of $u(t)y(t+\tau)$ equals

$$\int_{-\infty}^{\infty} G(\alpha)R_u(\tau-\alpha)d\alpha. \blacksquare$$

2. $R_y = G * G_{\text{rev}} * R_u$ where $G_{\text{rev}}(t) := G(-t)$

**Proof** Using (2.1) we get

$$y(t)y(t+\tau) = \int_{-\infty}^{\infty} G(\alpha)y(t)u(t+\tau-\alpha)d\alpha,$$

so the average value of $y(t)y(t+\tau)$ equals

$$\int_{-\infty}^{\infty} G(\alpha)R_{yu}(\tau-\alpha)d\alpha$$

(i.e., $R_y = G * R_{yu}$). Similarly, you can check that $R_{yu} = G_{\text{rev}} * R_u$. ■

3. $S_y(j\omega) = |\hat{G}(j\omega)|^2 S_u(j\omega)$

**Proof** From the previous fact we have

$$S_y(j\omega) = \hat{G}(j\omega)\hat{G}_{\text{rev}}(j\omega)S_u(j\omega),$$

so it remains to show that the Fourier transform of $G_{\text{rev}}$ equals the complex-conjugate of $\hat{G}(j\omega)$. This is easy. ■

## 2.5 Proofs for Tables 2.1 and 2.2 (Optional)

**Table 2.1**

**Entry (1,1)** If $u = \delta$, then $y = G$, so $\|y\|_2 = \|G\|_2$. But by Parseval's theorem, $\|G\|_2 = \|\hat{G}\|_2$.

**Entry (2,1)** Again, since $y = G$.

**Entry (3,1)**

$$\begin{aligned} pow(y)^2 &= \lim \frac{1}{2T}\int_0^T G(t)^2 dt \\ &\le \lim \frac{1}{2T}\int_0^\infty G(t)^2 dt \\ &= \lim \frac{1}{2T}\|G\|_2^2 \\ &= 0 \end{aligned}$$

**Entry (1,2)** With the input $u(t) = \sin(\omega t)$, the output is

$$y(t) = |\hat{G}(j\omega)| \sin[\omega t + \arg \hat{G}(j\omega)]. \tag{2.2}$$

The 2-norm of this signal is infinite as long as $\hat{G}(j\omega) \neq 0$, that is, the system's transfer function does not have a zero at the frequency of excitation.

**Entry (2,2)** The amplitude of the sinusoid (2.2) equals $|\hat{G}(j\omega)|$.

**Entry (3,2)** Let $\phi := \arg \hat{G}(j\omega)$. Then

$$
\begin{aligned}
pow(y)^2 &= \lim \frac{1}{2T} \int_{-T}^{T} |\hat{G}(j\omega)|^2 \sin^2(\omega t + \phi) dt \\
&= |\hat{G}(j\omega)|^2 \lim \frac{1}{2T} \int_{-T}^{T} \sin^2(\omega t + \phi) dt \\
&= |\hat{G}(j\omega)|^2 \lim \frac{1}{2\omega T} \int_{-\omega T + \phi}^{\omega T + \phi} \sin^2(\theta) d\theta \\
&= |\hat{G}(j\omega)|^2 \frac{1}{\pi} \int_{0}^{\pi} \sin^2(\theta) d\theta \\
&= \frac{1}{2} |\hat{G}(j\omega)|^2.
\end{aligned}
$$

**Table 2.2**

**Entry (1,1)** First we see that $\|\hat{G}\|_\infty$ is an upper bound on the 2-norm/2-norm system gain:

$$
\begin{aligned}
\|y\|_2^2 &= \|\hat{y}\|_2^2 \\
&= \frac{1}{2\pi} \int_{-\infty}^{\infty} |\hat{G}(j\omega)|^2 |\hat{u}(j\omega)|^2 d\omega \\
&\leq \|\hat{G}\|_\infty^2 \frac{1}{2\pi} \int_{-\infty}^{\infty} |\hat{u}(j\omega)|^2 d\omega \\
&= \|\hat{G}\|_\infty^2 \|\hat{u}\|_2^2 \\
&= \|\hat{G}\|_\infty^2 \|u\|_2^2.
\end{aligned}
$$

To show that $\|\hat{G}\|_\infty$ is the least upper bound, first choose a frequency $\omega_o$ where $|\hat{G}(j\omega)|$ is maximum, that is,

$$|\hat{G}(j\omega_o)| = \|\hat{G}\|_\infty.$$

Now choose the input $u$ so that

$$
|\hat{u}(j\omega)| = \begin{cases} c, & \text{if } |\omega - \omega_o| < \epsilon \text{ or } |\omega + \omega_o| < \epsilon \\ 0, & \text{otherwise,} \end{cases}
$$

where $\epsilon$ is a small positive number and $c$ is chosen so that $u$ has unit 2-norm (i.e., $c = \sqrt{\pi/2\epsilon}$). Then

$$
\begin{aligned}
\|\hat{y}\|_2^2 &\approx \frac{1}{2\pi} \left[ |\hat{G}(-j\omega_o)|^2 \pi + |\hat{G}(j\omega_o)|^2 \pi \right] \\
&= |\hat{G}(j\omega_o)|^2 \\
&= \|\hat{G}\|_\infty^2.
\end{aligned}
$$

**Entry (2,1)** This is an application of the Cauchy-Schwarz inequality:

$$\begin{aligned}
|y(t)| &= \left|\int_{-\infty}^{\infty} G(t-\tau)u(\tau)d\tau\right| \\
&\leq \left(\int_{-\infty}^{\infty} G(t-\tau)^2 d\tau\right)^{1/2} \left(\int_{-\infty}^{\infty} u(\tau)^2 d\tau\right)^{1/2} \\
&= \|G\|_2\|u\|_2 \\
&= \|\hat{G}\|_2\|u\|_2.
\end{aligned}$$

Hence

$$\|y\|_\infty \leq \|\hat{G}\|_2\|u\|_2.$$

To show that $\|\hat{G}\|_2$ is the least upper bound, apply the input

$$u(t) = G(-t)/\|G\|_2.$$

Then $\|u\|_2 = 1$ and $|y(0)| = \|G\|_2$, so $\|y\|_\infty \geq \|G\|_2$.

**Entry (3,1)** If $\|u\|_2 \leq 1$, then the 2-norm of $y$ is finite [as in entry (1,1)], so $pow(y) = 0$.

**Entry (1,2)** Apply a sinusoidal input of unit amplitude and frequency $\omega$ such that $j\omega$ is not a zero of $\hat{G}$. Then $\|u\|_\infty = 1$, but $\|y\|_2 = \infty$.

**Entry (2,2)** First, $\|G\|_1$ is an upper bound on the $\infty$-norm/$\infty$-norm system gain:

$$\begin{aligned}
|y(t)| &= \left|\int_{-\infty}^{\infty} G(\tau)u(t-\tau)d\tau\right| \\
&\leq \int_{-\infty}^{\infty} |G(\tau)u(t-\tau)|\, d\tau \\
&\leq \int_{-\infty}^{\infty} |G(\tau)|\, d\tau \|u\|_\infty \\
&= \|G\|_1\|u\|_\infty.
\end{aligned}$$

That $\|G\|_1$ is the least upper bound can be seen as follows. Fix $t$ and set

$$u(t-\tau) := \operatorname{sgn}(G(\tau)), \qquad \forall\tau.$$

Then $\|u\|_\infty = 1$ and

$$\begin{aligned}
y(t) &= \int_{-\infty}^{\infty} G(\tau)u(t-\tau)d\tau \\
&= \int_{-\infty}^{\infty} |G(\tau)|d\tau \\
&= \|G\|_1.
\end{aligned}$$

So $\|y\|_\infty \geq \|G\|_1$.

**Entry (3,2)** If $u$ is a power signal and $\|u\|_\infty \leq 1$, then $pow(u) \leq 1$, so

$$\sup\{pow(y) : \|u\|_\infty \leq 1\} \leq \sup\{pow(y) : pow(u) \leq 1\}.$$

We will see in entry (3,3) that the latter supremum equals $\|\hat{G}\|_\infty$.

**Entry (1,3)** If $u$ is a power signal, then from the preceding section,

$$S_y(j\omega) = |\hat{G}(j\omega)|^2 S_u(j\omega),$$

so

$$pow(y)^2 = \frac{1}{2\pi}\int_{-\infty}^{\infty} |\hat{G}(j\omega)|^2 S_u(j\omega)d\omega. \tag{2.3}$$

Unless $|\hat{G}(j\omega)|^2 S_u(j\omega)$ equals zero for all $\omega$, $pow(y)$ is positive, in which case its 2-norm is infinite.

**Entry (2,3)** This case is not so important, so a complete proof is omitted. The main idea is this: If $pow(u) \leq 1$, then $pow(y)$ is finite but $\|y\|_\infty$ is not necessarily (see $u_8$ in Exercise 2). So for a proof of this entry, one should construct an input with $pow(u) \leq 1$, but such that $\|y\|_\infty = \infty$.

**Entry (3,3)** From (2.3) we get immediately that

$$pow(y) \leq \|\hat{G}\|_\infty pow(u).$$

To achieve equality, suppose that

$$|\hat{G}(j\omega_o)| = \|\hat{G}\|_\infty$$

and let the input be

$$u(t) = \sqrt{2}\sin(\omega_o t).$$

Then $R_u(\tau) = \cos(\omega_o \tau)$, so

$$pow(u) = R_u(0) = 1.$$

Also,

$$S_u(j\omega) = \pi\left[\delta(\omega - \omega_o) + \delta(\omega + \omega_o)\right],$$

so from (2.3)

$$\begin{aligned} pow(y)^2 &= \frac{1}{2}|\hat{G}(j\omega_o)|^2 + \frac{1}{2}|\hat{G}(-j\omega_o)|^2 \\ &= |\hat{G}(j\omega_o)|^2 \\ &= \|\hat{G}\|_\infty^2. \end{aligned}$$

## 2.6 Computing by State-Space Methods (Optional)

This book is on classical control, which is set in the frequency domain. Current widespread practice, however, is to do computations using state-space methods. The purpose of this optional section is to illustrate how this is done for the problem of computing the 2-norm and $\infty$-norm of a transfer function. The derivation of the procedures is brief.

Consider a state-space model of the form

$$\begin{aligned} \dot{x}(t) &= Ax(t) + Bu(t), \\ y(t) &= Cx(t). \end{aligned}$$

Here $u(t)$ is the input signal and $y(t)$ the output signal, both scalar-valued. In contrast, $x(t)$ is a vector-valued function with, say, $n$ components. The dot in $\dot{x}$ means take the derivative of each component. Then $A$, $B$, $C$ are real matrices of sizes

$$n \times n, \quad n \times 1, \quad 1 \times n.$$

The equations are assumed to hold for $t \geq 0$. Take Laplace transforms with zero initial conditions on $x$:

$$\begin{aligned} s\hat{x}(s) &= A\hat{x}(s) + B\hat{u}(s), \\ \hat{y}(s) &= C\hat{x}(s). \end{aligned}$$

Now eliminate $\hat{x}(s)$ to get

$$\hat{y}(s) = C(sI - A)^{-1}B\hat{u}(s).$$

We conclude that the transfer function from $\hat{u}$ to $\hat{y}$ is

$$\hat{G}(s) = C(sI - A)^{-1}B.$$

This transfer function is strictly proper. [Try an example: start with some $A$, $B$, $C$ with $n = 2$, and compute $\hat{G}(s)$.]

Going the other way, from a strictly proper transfer function to a state-space model, is more profound, but it is true that for every strictly proper transfer function $\hat{G}(s)$ there exist $(A, B, C)$ such that

$$\hat{G}(s) = C(sI - A)^{-1}B.$$

From the representation

$$\hat{G}(s) = \frac{1}{\det(sI - A)} C \operatorname{adj}(sI - A)B$$

it should be clear that the poles of $\hat{G}(s)$ are included in the eigenvalues of $A$. We say that $A$ is *stable* if all its eigenvalues lie in $\operatorname{Re} s < 0$, in which case $\hat{G}$ is a stable transfer function.

Now start with the representation

$$\hat{G}(s) = C(sI - A)^{-1}B$$

with $A$ stable. We want to compute $\|\hat{G}\|_2$ and $\|\hat{G}\|_\infty$ from the data $(A, B, C)$.

### The 2-Norm

Define the matrix exponential

$$e^{tA} := I + tA + \frac{t^2}{2!}A^2 + \cdots$$

just as if $A$ were a scalar (convergence can be proved). Let a prime denote transpose and define the matrix

$$L := \int_0^\infty e^{tA}BB'e^{tA'}\,dt$$

(the integral converges because $A$ is stable). Then $L$ satisfies the equation

$$AL + LA' + BB' = 0.$$

**Proof** Integrate both sides of the equation

$$\frac{d}{dt}\mathrm{e}^{tA}BB'\mathrm{e}^{tA'} = A\mathrm{e}^{tA}BB'\mathrm{e}^{tA'} + \mathrm{e}^{tA}BB'\mathrm{e}^{tA'}A'$$

from 0 to $\infty$, noting that $\exp(tA)$ converges to 0 because $A$ is stable, to get

$$-BB' = AL + LA'. \blacksquare$$

In terms of $L$ a simple formula for the 2-norm of $\hat{G}$ is

$$\|\hat{G}\|_2 = (CLC')^{1/2}.$$

**Proof** The impulse response function is

$$G(t) = C\mathrm{e}^{tA}B, \quad t > 0.$$

Calling on Parseval we get

$$\begin{aligned} \|\hat{G}\|_2^2 &= \|G\|_2^2 \\ &= \int_0^\infty C\mathrm{e}^{tA}BB'\mathrm{e}^{tA'}C'dt \\ &= C\int_0^\infty \mathrm{e}^{tA}BB'\mathrm{e}^{tA'}dtC' \\ &= CLC'. \blacksquare \end{aligned}$$

So a procedure to compute the 2-norm is as follows:

**Step 1** Solve the equation

$$AL + LA' + BB' = 0$$

for the matrix $L$.

**Step 2**

$$\|\hat{G}\|_2 = (CLC')^{1/2}$$

### The $\infty$-Norm

Computing the $\infty$-norm is harder; we shall have to be content with a search procedure. Define the $2n \times 2n$ matrix

$$H := \begin{bmatrix} A & BB' \\ -C'C & -A' \end{bmatrix}.$$

**Theorem 1** $\|\hat{G}\|_\infty < 1$ *iff* $H$ *has no eigenvalues on the imaginary axis.*

**Proof** The proof of this theorem is a bit involved, so only sufficiency is considered, and it is only sketched.

It is not too hard to derive that

$$1/[1 - \hat{G}(-s)\hat{G}(s)] = 1 + \begin{bmatrix} 0 & B' \end{bmatrix} (sI - H)^{-1} \begin{bmatrix} B \\ 0 \end{bmatrix}.$$

Thus the poles of $[1-\hat{G}(-s)\hat{G}(s)]^{-1}$ are contained in the eigenvalues of $H$.

Assume that $H$ has no eigenvalues on the imaginary axis. Then $[1-\hat{G}(-s)\hat{G}(s)]^{-1}$ has no poles there, so $1-\hat{G}(-s)\hat{G}(s)$ has no zeros there, that is,

$$|\hat{G}(j\omega)| \neq 1, \quad \forall\omega.$$

Since $\hat{G}$ is strictly proper, this implies that

$$|\hat{G}(j\omega)| < 1, \quad \forall\omega$$

(i.e., $\|\hat{G}\|_\infty < 1$). ■

The theorem suggests this way to compute an $\infty$-norm: Select a positive number $\gamma$; test if $\|\hat{G}\|_\infty < \gamma$ (i.e., if $\|\gamma^{-1}\hat{G}\|_\infty < 1$) by calculating the eigenvalues of the appropriate matrix; increase or decrease $\gamma$ accordingly; repeat. A bisection search is quite efficient: Get upper and lower bounds for $\|\hat{G}\|_\infty$; try $\gamma$ midway between these bounds; continue.

## Exercises

1. Suppose that $u(t)$ is a continuous signal whose derivative $\dot{u}(t)$ is continuous too. Which of the following qualifies as a norm for $u$?

$$\sup_t |\dot{u}(t)|$$

$$|u(0)| + \sup_t |\dot{u}(t)|$$

$$\max\{\sup_t |u(t)|, \sup_t |\dot{u}(t)|\}$$

$$\sup_t |u(t)| + \sup_t |\dot{u}(t)|$$

2. Consider the Venn diagram in Figure 2.1. Show that the functions $u_1$ to $u_9$, defined below, are located in the diagram as shown in Figure 2.2. All the functions are zero for $t < 0$.

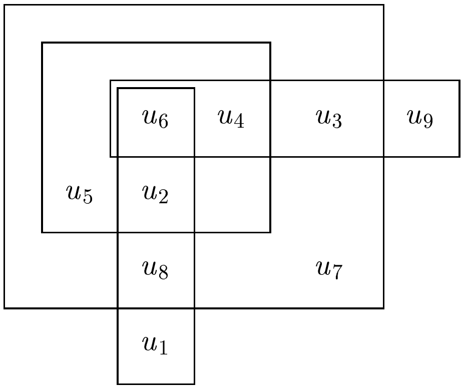


Figure 2.2: Figure for Exercise 2.

$$\begin{aligned}
u_1(t) &= \begin{cases} 1/\sqrt{t}, & \text{if } t \le 1 \\ 0, & \text{if } t > 1 \end{cases} \\
u_2(t) &= \begin{cases} 1/t^{1/4}, & \text{if } t \le 1 \\ 0, & \text{if } t > 1 \end{cases} \\
u_3(t) &= 1 \\
u_4(t) &= 1/(1+t) \\
u_5(t) &= u_2(t) + u_4(t) \\
u_6(t) &= 0 \\
u_7(t) &= u_2(t) + 1
\end{aligned}$$

For $u_8$, set

$$v_k(t) = \begin{cases} k, & \text{if } k < t < k + k^{-3} \\ 0, & \text{otherwise} \end{cases}$$

and then

$$u_8(t) = \sum_1^\infty v_k(t).$$

Finally, let $u_9$ equal 1 in the intervals

$$[2^{2k}, 2^{2k+1}], \quad k = 0, 1, 2, \ldots$$

and zero elsewhere.

3. Suppose that $\hat{G}(s)$ is a real-rational, stable transfer function with $\hat{G}^{-1}$ stable, too (i.e., neither poles nor zeros in $\text{Re}\ s \ge 0$). True or false: The Bode phase plot, $\angle\hat{G}(j\omega)$ versus $\omega$, can be uniquely constructed from the Bode magnitude plot, $|\hat{G}(j\omega)|$ versus $\omega$. (Answer: false!)

4. Recall that the transfer function for a pure timedelay of $\tau$ time units is

$$\hat{D}(s) := \mathrm{e}^{-s\tau}.$$

Say that a norm $\| \ \|$ on transfer functions is *time-delay invariant* if for every transfer function $\hat{G}$ (such that $\|\hat{G}\| < \infty$) and every $\tau > 0$,

$$\|\hat{D}\hat{G}\| = \|\hat{G}\|.$$

Is the 2-norm or $\infty$-norm time-delay invariant?

5. Compute the 1-norm of the impulse response corresponding to the transfer function

$$\frac{1}{\tau s + 1}, \quad \tau > 0.$$

6. For $\hat{G}$ stable and strictly proper, show that $\|G\|_1 < \infty$ and find an inequality relating $\|\hat{G}\|_\infty$ and $\|G\|_1$.

7. This concerns entry (2,2) in Table 2.2. The given entry assumes that $\hat{G}$ is stable and strictly proper. When $\hat{G}$ is stable but only proper, it can be expressed as

$$\hat{G}(s) = c + \hat{G}_1(s)$$

with $c$ constant and $\hat{G}_1$ stable and strictly proper. Show that the correct (2,2)-entry is

$$|c| + \|G_1\|_1.$$

8. Show that entries (2,2) and (3,2) in Table 2.1 and entries (1,1), (3,2), and (3,3) in Table 2.2 hold when $\hat{G}$ is stable and proper (instead of strictly proper).

9. Let $\hat{G}(s)$ be a strictly proper stable transfer function and $G(t)$ its inverse Laplace transform. Let $u(t)$ be a signal of finite 1-norm. True or false:

$$\|G * u\|_1 \leq \|G\|_1 \|u\|_1?$$

10. Consider a system with transfer function

$$\frac{\omega_n^2}{s^2 + 2\zeta\omega_n s + \omega_n^2}, \quad \zeta, \omega_n > 0,$$

and input

$$u(t) = \sin 0.1t, \quad -\infty < t < \infty.$$

Compute *pow* of the output.

11. Consider a system with transfer function

$$\frac{s+2}{4s+1}$$

and input $u$ and output $y$. Compute

$$\sup_{\|u\|_\infty = 1} \|y\|_\infty$$

and find an input achieving this supremum.

12. For a linear system with input $u(t)$ and output $y(t)$, prove that

$$\sup_{\|u\| \leq 1} \|y\| = \sup_{\|u\| = 1} \|y\|$$

where the norm is, say, the 2-norm.

13. Show that the 2-norm for transfer functions is not submultiplicative.

14. Write a MATLAB program to compute the $\infty$-norm of a transfer function using the grid method. Test your program on the function

$$\frac{1}{s^2 + 10^{-6}s + 1}$$

and compare your answer to the exact solution computed by hand using the derivative method.

## Notes and References

The material in this chapter belongs to the field of mathematics called functional analysis. Tools from functional analysis were introduced into the subject of feedback control around 1960 by G. Zames and I. Sandberg. Some references are Desoer and Vidyasagar (1975), Holtzman (1970), Mees (1981), and Willems (1971). The state-space procedure for the $\infty$-norm is from Boyd et al. (1989).

# Chapter 3

# Basic Concepts

This chapter and the next are the most fundamental. We concentrate on the single-loop feedback system. Stability of this system is defined and characterized. Then the system is analyzed for its ability to track certain signals (i.e., steps and ramps) asymptotically as time increases. Finally, tracking is addressed as a performance specification. Uncertainty is postponed until the next chapter.

Now a word about notation. In the preceding chapter we used signals in the time and frequency domains; the notation was $u(t)$ for a function of time and $\hat{u}(s)$ for its Laplace transform. When the context is solely the frequency domain, it is convenient to drop the hat and write $u(s)$; similarly for an impulse response $G(t)$ and the corresponding transfer function $\hat{G}(s)$.

## 3.1 Basic Feedback Loop

The most elementary feedback control system has three components: a plant (the object to be controlled, no matter what it is, is always called the *plant*), a sensor to measure the output of the plant, and a controller to generate the plant's input. Usually, actuators are lumped in with the plant. We begin with the block diagram in Figure 3.1. Notice that each of the three components

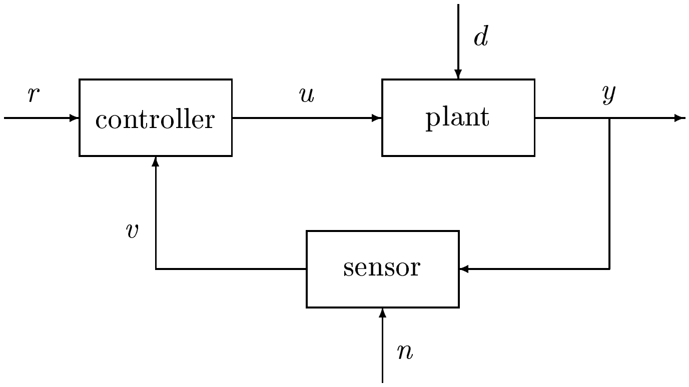


Figure 3.1: Elementary control system.

has two inputs, one internal to the system and one coming from outside, and one output. These signals have the following interpretations:

| | |
|---|---|
| $r$ | reference or command input |
| $v$ | sensor output |
| $u$ | actuating signal, plant input |
| $d$ | external disturbance |
| $y$ | plant output and measured signal |
| $n$ | sensor noise |

The three signals coming from outside—$r$, $d$, and $n$—are called *exogenous inputs.*

In what follows we shall consider a variety of performance objectives, but they can be summarized by saying that $y$ should approximate some prespecified function of $r$, and it should do so in the presence of the disturbance $d$, sensor noise $n$, with uncertainty in the plant. We may also want to limit the size of $u$. Frequently, it makes more sense to describe the performance objective in terms of the measurement $v$ rather than $y$, since often the only knowledge of $y$ is obtained from $v$.

The analysis to follow is done in the frequency domain. To simplify notation, hats are omitted from Laplace transforms.

Each of the three components in Figure 3.1 is assumed to be linear, so its output is a linear function of its input, in this case a two-dimensional vector. For example, the plant equation has the form

$$y = P \begin{pmatrix} d \\ u \end{pmatrix}.$$

Partitioning the $1 \times 2$ transfer matrix $P$ as

$$P = \begin{bmatrix} P_1 & P_2 \end{bmatrix},$$

we get

$$y = P_1 d + P_2 u.$$

We shall take an even more specialized viewpoint and suppose that the outputs of the three components are linear functions of the sums (or difference) of their inputs; that is, the plant, sensor, and controller equations are taken to be of the form

$$\begin{aligned} y &= P(d+u), \\ v &= F(y+n), \\ u &= C(r-v). \end{aligned}$$

The minus sign in the last equation is a matter of tradition. The block diagram for these equations is in Figure 3.2. Our convention is that plus signs at summing junctions are omitted.

This section ends with the notion of *well-posedness.* This means that in Figure 3.2 all closed-loop transfer functions exist, that is, all transfer functions from the three exogenous inputs to all internal signals, namely, $u$, $y$, $v$, and the outputs of the summing junctions. Label the outputs of the summing junctions as in Figure 3.3. For well-posedness it suffices to look at the nine transfer functions from $r$, $d$, $n$ to $x_1$, $x_2$, $x_3$. (The other transfer functions are obtainable from these.) Write the equations at the summing junctions:

$$\begin{aligned} x_1 &= r - F x_3, \\ x_2 &= d + C x_1, \\ x_3 &= n + P x_2. \end{aligned}$$

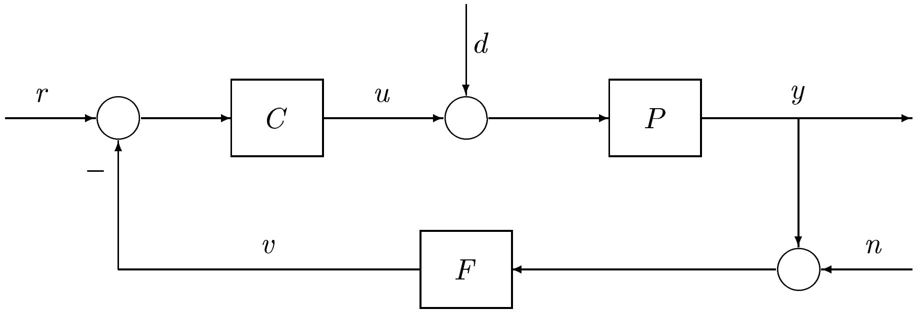


Figure 3.2: Basic feedback loop.

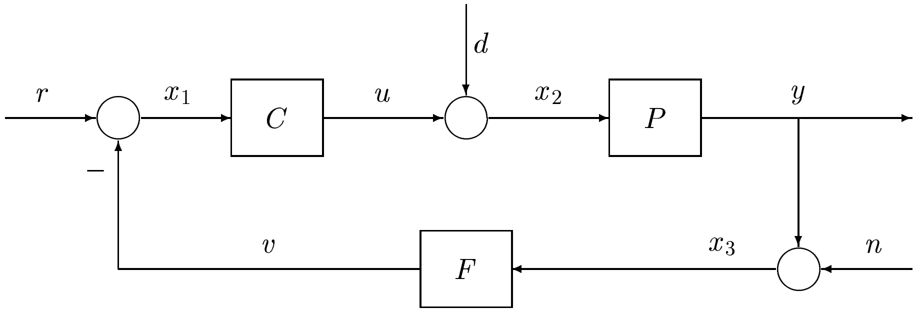


Figure 3.3: Basic feedback loop.

In matrix form these are

$$\begin{bmatrix} 1 & 0 & F \\ -C & 1 & 0 \\ 0 & -P & 1 \end{bmatrix} \begin{pmatrix} x_1 \\ x_2 \\ x_3 \end{pmatrix} = \begin{pmatrix} r \\ d \\ n \end{pmatrix}.$$

Thus, the system is well-posed iff the above $3 \times 3$ matrix is nonsingular, that is, the determinant $1 + PCF$ is not identically equal to zero. [For instance, the system with $P(s) = 1$, $C(s) = 1$, $F(s) = -1$ is not well-posed.] Then the nine transfer functions are obtained from the equation

$$\begin{pmatrix} x_1 \\ x_2 \\ x_3 \end{pmatrix} = \begin{bmatrix} 1 & 0 & F \\ -C & 1 & 0 \\ 0 & -P & 1 \end{bmatrix}^{-1} \begin{pmatrix} r \\ d \\ n \end{pmatrix},$$

that is,

$$\begin{pmatrix} x_1 \\ x_2 \\ x_3 \end{pmatrix} = \frac{1}{1+PCF} \begin{bmatrix} 1 & -PF & -F \\ C & 1 & -CF \\ PC & P & 1 \end{bmatrix} \begin{pmatrix} r \\ d \\ n \end{pmatrix}. \tag{3.1}$$

A stronger notion of well-posedness that makes sense when $P$, $C$, and $F$ are proper is that the nine transfer functions above are proper. A necessary and sufficient condition for this is that $1 + PCF$ not be strictly proper [i.e., $PCF(\infty) \neq -1$].

One might argue that the transfer functions of all physical systems are strictly proper: If a sinusoid of ever-increasing frequency is applied to a (linear, time-invariant) system, the amplitude

of the output will go to zero. This is somewhat misleading because a real system will cease to behave linearly as the frequency of the input increases. Furthermore, our transfer functions will be used to parametrize an uncertainty set, and as we shall see, it may be convenient to allow some of them to be only proper. A proportional-integral-derivative controller is very common in practice, especially in chemical engineering. It has the form

$$k_1 + \frac{k_2}{s} + k_3 s.$$

This is not proper, but it can be approximated over any desired frequency range by a proper one, for example,

$$k_1 + \frac{k_2}{s} + \frac{k_3 s}{\tau s + 1}.$$

Notice that the feedback system is automatically well-posed, in the stronger sense, if $P$, $C$, and $F$ are proper and one is strictly proper. For most of the book, we shall make the following *standing assumption*, under which the nine transfer functions in (3.1) are proper:

$P$ is strictly proper, $C$ and $F$ are proper.

However, at times it will be convenient to require only that $P$ be proper. In this case we shall always assume that $|PCF| < 1$ at $\omega = \infty$, which ensures that $1 + PCF$ is not strictly proper. Given that no model, no matter how complex, can approximate a real system at sufficiently high frequencies, we should be very uncomfortable if $|PCF| > 1$ at $\omega = \infty$, because such a controller would almost surely be unstable if implemented on a real system.

## 3.2 Internal Stability

Consider a system with input $u$, output $y$, and transfer function $\hat{G}$, assumed stable and proper. We can write

$$\hat{G} = G_0 + \hat{G}_1,$$

where $G_0$ is a constant and $\hat{G}_1$ is strictly proper.

$$\text{Example:} \quad \frac{s}{s+1} = 1 - \frac{1}{s+1}.$$

In the time domain the equation is

$$y(t) = G_0 u(t) + \int_{-\infty}^{\infty} G_1(t-\tau)u(\tau)\ d\tau.$$

If $|u(t)| \le c$ for all $t$, then

$$|y(t)| \le |G_0|c + \int_{-\infty}^{\infty} |G_1(\tau)|\ d\tau c.$$

The right-hand side is finite. Thus the output is bounded whenever the input is bounded. [This argument is the basis for entry (2,2) in Table 2.2.]

If the nine transfer functions in (3.1) are stable, then the feedback system is said to be *internally stable*. As a consequence, if the exogenous inputs are bounded in magnitude, so too are $x_1$, $x_2$, and $x_3$, and hence $u$, $y$, and $v$. So internal stability guarantees bounded internal signals for all bounded exogenous signals.

The idea behind this definition of internal stability is that it is not enough to look only at input-output transfer functions, such as from $r$ to $y$, for example. This transfer function could be stable, so that $y$ is bounded when $r$ is, and yet an internal signal could be unbounded, probably causing internal damage to the physical system.

For the remainder of this section hats are dropped.

**Example** In Figure 3.3 take

$$C(s) = \frac{s-1}{s+1}, \quad P(s) = \frac{1}{s^2-1}, \quad F(s) = 1.$$

Check that the transfer function from $r$ to $y$ is stable, but that from $d$ to $y$ is not. The feedback system is therefore not internally stable. As we will see later, this offense is caused by the cancellation of the controller zero and the plant pole at the point $s = 1$.

We shall develop a test for internal stability which is easier than examining nine transfer functions. Write $P$, $C$, and $F$ as ratios of coprime polynomials (i.e., polynomials with no common factors):

$$P = \frac{N_P}{M_P}, \quad C = \frac{N_C}{M_C}, \quad F = \frac{N_F}{M_F}.$$

The *characteristic polynomial* of the feedback system is the one formed by taking the product of the three numerators plus the product of the three denominators:

$$N_P N_C N_F + M_P M_C M_F.$$

The *closed-loop poles* are the zeros of the characteristic polynomial.

**Theorem 1** *The feedback system is internally stable iff there are no closed-loop poles in* $\text{Re}s \geq 0$.

**Proof** For simplicity assume that $F = 1$; the proof in the general case is similar, but a bit messier.

From (3.1) we have

$$\begin{pmatrix} x_1 \\ x_2 \\ x_3 \end{pmatrix} = \frac{1}{1+PC} \begin{bmatrix} 1 & -P & -1 \\ C & 1 & -C \\ PC & P & 1 \end{bmatrix} \begin{pmatrix} r \\ d \\ n \end{pmatrix}.$$

Substitute in the ratios and clear fractions to get

$$\begin{pmatrix} x_1 \\ x_2 \\ x_3 \end{pmatrix} = \frac{1}{N_P N_C + M_P M_C} \begin{bmatrix} M_P M_C & -N_P M_C & -M_P M_C \\ M_P N_C & M_P M_C & -M_P N_C \\ N_P N_C & N_P M_C & M_P M_C \end{bmatrix} \begin{pmatrix} r \\ d \\ n \end{pmatrix}. \tag{3.2}$$

Note that the characteristic polynomial equals $N_P N_C + M_P M_C$. Sufficiency is now evident; the feedback system is internally stable if the characteristic polynomial has no zeros in $\text{Re}s \geq 0$.

Necessity involves a subtle point. Suppose that the feedback system is internally stable. Then all nine transfer functions in (3.2) are stable, that is, they have no poles in $\text{Re}\, s \geq 0$. But we cannot immediately conclude that the polynomial $N_P N_C + M_P M_C$ has no zeros in $\text{Re}s \geq 0$ because this polynomial may conceivably have a right half-plane zero which is also a zero of all nine numerators in (3.2), and hence is canceled to form nine stable transfer functions. However, the characteristic polynomial has no zero which is also a zero of all nine numerators, $M_P M_C$, $N_P M_C$, and so on.

Proof of this statement is left as an exercise. (It follows from the fact that we took coprime factors to start with, that is, $N_P$ and $M_P$ are coprime, as are the other numerator-denominator pairs.) ■

By Theorem 1 internal stability can be determined simply by checking the zeros of a polynomial. There is another test that provides additional insight.

**Theorem 2** *The feedback system is internally stable iff the following two conditions hold:*

*(a) The transfer function $1 + PCF$ has no zeros in Re$s \geq 0$.*

*(b) There is no pole-zero cancellation in Re$s \geq 0$ when the product $PCF$ is formed.*

**Proof** Recall that the feedback system is internally stable iff all nine transfer functions

$$\frac{1}{1+PCF}\begin{bmatrix} 1 & -PF & -F \\ C & 1 & -CF \\ PC & P & 1 \end{bmatrix}$$

are stable.

($\Rightarrow$) Assume that the feedback system is internally stable. Then in particular $(1 + PCF)^{-1}$ is stable (i.e., it has no poles in Re$s \geq 0$). Hence $1 + PCF$ has no zeros there. This proves (a).

To prove (b), write $P, C, F$ as ratios of coprime polynomials:

$$P = \frac{N_P}{M_P}, \quad C = \frac{N_C}{M_C}, \quad F = \frac{N_F}{M_F}.$$

By Theorem 1 the characteristic polynomial

$$N_P N_C N_F + M_P M_C M_F$$

has no zeros in Re$s \geq 0$. Thus the pair $(N_P, M_C)$ have no common zero in Re$s \geq 0$, and similarly for the other numerator-denominator pairs.

($\Leftarrow$) Assume (a) and (b). Factor $P, C, F$ as above, and let $s_0$ be a zero of the characteristic polynomial, that is,

$$(N_P N_C N_F + M_P M_C M_F)(s_0) = 0.$$

We must show that Re$s_0 < 0$; this will prove internal stability by Theorem 1. Suppose to the contrary that Re$s_0 \geq 0$. If

$$(M_P M_C M_F)(s_0) = 0,$$

then

$$(N_P N_C N_F)(s_0) = 0.$$

But this violates (b). Thus

$$(M_P M_C M_F)(s_0) \neq 0,$$

so we can divide by it above to get

$$1 + \frac{N_P N_C N_F}{M_P M_C M_F}(s_0) = 0,$$

that is,

$$1 + (PCF)(s_0) = 0,$$

which violates (a). ■

Finally, let us recall for later use the Nyquist stability criterion. It can be derived from Theorem 2 and the principle of the argument. Begin with the curve $\mathcal{D}$ in the complex plane: It starts at the origin, goes up the imaginary axis, turns into the right half-plane following a semicircle of infinite radius, and comes up the negative imaginary axis to the origin again:

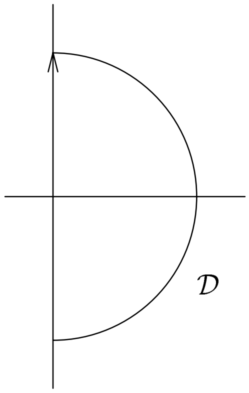


As a point $s$ makes one circuit around this curve, the point $P(s)C(s)F(s)$ traces out a curve called the *Nyquist plot* of $PCF$. If $PCF$ has a pole on the imaginary axis, then $\mathcal{D}$ must have a small indentation to avoid it.

**Nyquist Criterion** *Construct the Nyquist plot of $PCF$, indenting to the left around poles on the imaginary axis. Let $n$ denote the total number of poles of $P$, $C$, and $F$ in Res $\geq 0$. Then the feedback system is internally stable iff the Nyquist plot does not pass through the point -1 and encircles it exactly $n$ times counterclockwise.*

## 3.3 Asymptotic Tracking

In this section we look at a typical performance specification, perfect asymptotic tracking of a reference signal. Both time domain and frequency domain occur, so the notation distinction is required.

For the remainder of this chapter we specialize to the *unity-feedback* case, $\hat{F} = 1$, so the block diagram is as in Figure 3.4. Here $e$ is the tracking error; with $n = d = 0$, $e$ equals the reference input (ideal response), $r$, minus the plant output (actual response), $y$.

We wish to study this system's capability of tracking certain test inputs asymptotically as time tends to infinity. The two test inputs are the step

$$r(t) = \begin{cases} c, & \text{if } t \geq 0 \\ 0, & \text{if } t < 0 \end{cases}$$

and the ramp

$$r(t) = \begin{cases} ct, & \text{if } t \geq 0 \\ 0, & \text{if } t < 0 \end{cases}$$

($c$ is a nonzero real number). As an application of the former think of the temperature-control thermostat in a room; when you change the setting on the thermostat (step input), you would like

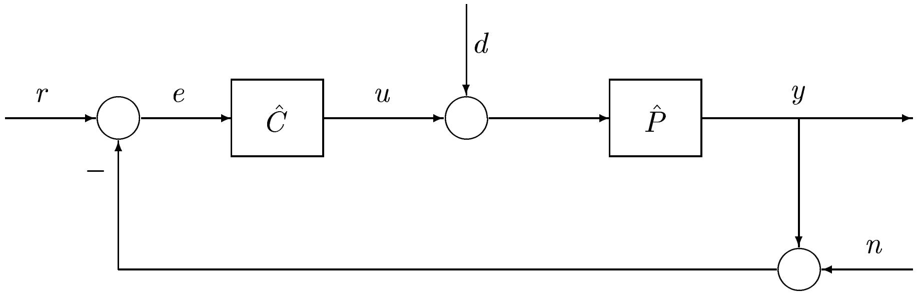


Figure 3.4: Unity-feedback loop.

the room temperature eventually to change to the new setting (of course, you would like the change to occur within a reasonable time). A situation with a ramp input is a radar dish designed to track orbiting satellites. A satellite moving in a circular orbit at constant angular velocity sweeps out an angle that is approximately a linear function of time (i.e., a ramp).

Define the *loop transfer function* $\hat{L} := \hat{P}\hat{C}$. The transfer function from reference input $r$ to tracking error $e$ is

$$\hat{S} := \frac{1}{1+\hat{L}},$$

called the *sensitivity function*—more on this in the next section. The ability of the system to track steps and ramps asymptotically depends on the number of zeros of $\hat{S}$ at $s = 0$.

**Theorem 3** *Assume that the feedback system is internally stable and $n = d = 0$.*

*(a) If $r$ is a step, then $e(t) \longrightarrow 0$ as $t \longrightarrow \infty$ iff $\hat{S}$ has at least one zero at the origin.*

*(b) If $r$ is a ramp, then $e(t) \longrightarrow 0$ as $t \longrightarrow \infty$ iff $\hat{S}$ has at least two zeros at the origin.*

The proof is an application of the *final-value theorem*: If $\hat{y}(s)$ is a rational Laplace transform that has no poles in $\text{Re}s \geq 0$ except possibly a simple pole at $s = 0$, then $\lim_{t\to\infty} y(t)$ exists and it equals $\lim_{s\to 0} s\hat{y}(s)$.

**Proof** (a) The Laplace transform of the foregoing step is $\hat{r}(s) = c/s$. The transfer function from $r$ to $e$ equals $\hat{S}$, so

$$\hat{e}(s) = \hat{S}(s)\frac{c}{s}.$$

Since the feedback system is internally stable, $\hat{S}$ is a stable transfer function. It follows from the final-value theorem that $e(t)$ does indeed converge as $t \longrightarrow \infty$, and its limit is the residue of the function $\hat{e}(s)$ at the pole $s = 0$:

$$e(\infty) = \hat{S}(0)c.$$

The right-hand side equals zero iff $\hat{S}(0) = 0$.

(b) Similarly with $\hat{r}(s) = c/s^2$. ■

Note that $\hat{S}$ has a zero at $s = 0$ iff $\hat{L}$ has a pole there. Thus, from the theorem we see that if the feedback system is internally stable and either $\hat{P}$ or $\hat{C}$ has a pole at the origin (i.e., an inherent integrator), then the output $y(t)$ will asymptotically track any step input $r$.

**Example** To see how this works, take the simplest possible example,

$$\hat{P}(s) = \frac{1}{s}, \quad \hat{C}(s) = 1.$$

Then the transfer function from $r$ to $e$ equals

$$\frac{1}{1+s^{-1}} = \frac{s}{s+1}.$$

So the open-loop pole at $s = 0$ becomes a closed-loop zero of the error transfer function; then this zero cancels the pole of $\hat{r}(s)$, resulting in no unstable poles in $\hat{e}(s)$. Similar remarks apply for a ramp input.

Theorem 3 is a special case of an elementary principle: For perfect asymptotic tracking, the loop transfer function $\hat{L}$ must contain an internal model of the unstable poles of $\hat{r}$.

A similar analysis can be done for the situation where $r = n = 0$ and $d$ is a sinusoid, say $d(t) = \sin(\omega t)1(t)$ (1 is the unit step). You can show this: If the feedback system is internally stable, then $y(t) \longrightarrow 0$ as $t \longrightarrow \infty$ iff either $\hat{P}$ has a zero at $s = j\omega$ or $\hat{C}$ has a pole at $s = j\omega$ (Exercise 3).

## 3.4 Performance

In this section we again look at tracking a reference signal, but whereas in the preceding section we considered perfect asymptotic tracking of a *single* signal, we will now consider a *set* of reference signals and a bound on the steady-state error. This performance objective will be quantified in terms of a weighted norm bound.

As before, let $L$ denote the loop transfer function, $L := PC$. The transfer function from reference input $r$ to tracking error $e$ is

$$S := \frac{1}{1+L},$$

called the *sensitivity function.* In the analysis to follow, it will always be assumed that the feedback system is internally stable, so $S$ is a stable, proper transfer function. Observe that since $L$ is strictly proper (since $P$ is), $S(j\infty) = 1$.

The name *sensitivity function* comes from the following idea. Let $T$ denote the transfer function from $r$ to $y$:

$$T = \frac{PC}{1+PC}.$$

One way to quantify how sensitive $T$ is to variations in $P$ is to take the limiting ratio of a relative perturbation in $T$ (i.e., $\Delta T/T$) to a relative perturbation in $P$ (i.e., $\Delta P/P$). Thinking of $P$ as a variable and $T$ as a function of it, we get

$$\lim_{\Delta P \to 0} \frac{\Delta T/T}{\Delta P/P} = \frac{dT}{dP}\frac{P}{T}.$$

The right-hand side is easily evaluated to be $S$. In this way, $S$ is the sensitivity of the closed-loop transfer function $T$ to an infinitesimal perturbation in $P$.

Now we have to decide on a performance specification, a measure of goodness of tracking. This decision depends on two things: what we know about $r$ and what measure we choose to assign to the tracking error. Usually, $r$ is not known in advance—few control systems are designed for one

and only one input. Rather, a set of possible $r$s will be known or at least postulated for the purpose of design.

Let's first consider sinusoidal inputs. Suppose that $r$ can be any sinusoid of amplitude $\leq 1$ and we want $e$ to have amplitude $< \epsilon$. Then the performance specification can be expressed succinctly as

$$\|S\|_\infty < \epsilon.$$

Here we used Table 2.1: the maximum amplitude of $e$ equals the $\infty$-norm of the transfer function. Or if we define the (trivial, in this case) weighting function $W_1(s) = 1/\epsilon$, then the performance specification is $\|W_1 S\|_\infty < 1$.

The situation becomes more realistic and more interesting with a frequency-dependent weighting function. Assume that $W_1(s)$ is real-rational; you will see below that only the magnitude of $W_1(j\omega)$ is relevant, so any poles or zeros in $\mathrm{Re}\, s > 0$ can be reflected into the left half-plane without changing the magnitude. Let us consider four scenarios giving rise to an $\infty$-norm bound on $W_1 S$. The first three require $W_1$ to be stable.

1. Suppose that the family of reference inputs is all signals of the form $r = W_1 r_{pf}$, where $r_{pf}$, a pre-filtered input, is any sinusoid of amplitude $\leq 1$. Thus the set of $r$s consists of sinusoids with frequency-dependent amplitudes. Then the maximum amplitude of $e$ equals $\|W_1 S\|_\infty$.

2. Recall from Chapter 2 that

$$\|r\|_2^2 = \frac{1}{2\pi} \int_{-\infty}^{\infty} |r(j\omega)|^2 \, d\omega$$

and that $\|r\|_2^2$ is a measure of the energy of $r$. Thus we may think of $|r(j\omega)|^2$ as *energy spectral density*, or energy spectrum. Suppose that the set of all $r$s is

$$\{r : r = W_1 r_{pf}, \|r_{pf}\|_2 \leq 1\},$$

that is,

$$\left\{ r : \frac{1}{2\pi} \int_{-\infty}^{\infty} |r(j\omega)/W_1(j\omega)|^2 \, d\omega \leq 1 \right\}.$$

Thus, $r$ has an energy constraint and its energy spectrum is weighted by $1/|W_1(j\omega)|^2$. For example, if $W_1$ were a bandpass filter, the energy spectrum of $r$ would be confined to the passband. More generally, $W_1$ could be used to shape the energy spectrum of the expected class of reference inputs. Now suppose that the tracking error measure is the 2-norm of $e$. Then from Table 2.2,

$$\sup_r \|e\|_2 = \sup\{\|SW_1 r_{pf}\|_2 : \|r_{pf}\|_2 \leq 1\} = \|W_1 S\|_\infty,$$

so $\|W_1 S\|_\infty < 1$ means that $\|e\|_2 < 1$ for all $r$s in the set above .

3. This scenario is like the preceding one except for signals of finite power. We see from Table 2.2 that $\|W_1 S\|_\infty$ equals the supremum of $pow(e)$ over all $r_{pf}$ with $pow(r_{pf}) \leq 1$. So $W_1$ could be used to shape the power spectrum of the expected class of $r$s.

4. In several applications, for example aircraft flight-control design, designers have acquired through experience desired shapes for the Bode magnitude plot of $S$. In particular, suppose that good performance is known to be achieved if the plot of $|S(j\omega)|$ lies under some curve. We could rewrite this as

$$|S(j\omega)| < |W_1(j\omega)|^{-1}, \quad \forall \omega,$$

or in other words, $\|W_1 S\|_\infty < 1$.

There is a nice graphical interpretation of the norm bound $\|W_1S\|_\infty < 1$. Note that

$$\begin{aligned}\|W_1S\|_\infty < 1 \quad &\Leftrightarrow \quad \left|\frac{W_1(j\omega)}{1+L(j\omega)}\right| < 1, \quad \forall\omega \\ &\Leftrightarrow \quad |W_1(j\omega)| < |1+L(j\omega)|, \quad \forall\omega.\end{aligned}$$

The last inequality says that at every frequency, the point $L(j\omega)$ on the Nyquist plot lies outside the disk of center -1, radius $|W_1(j\omega)|$ (Figure 3.5).

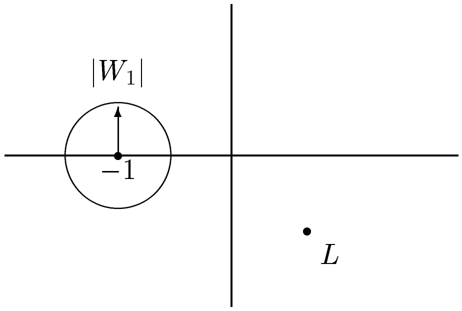


Figure 3.5: Performance specification graphically.

Other performance problems could be posed by focusing on the response to the other two exogenous inputs, $d$ and $n$. Note that the transfer functions from $d$, $n$ to $e$, $u$ are given by

$$\begin{bmatrix} e \\ u \end{bmatrix} = -\begin{bmatrix} PS & S \\ T & CS \end{bmatrix}\begin{bmatrix} d \\ n \end{bmatrix},$$

where

$$T := 1 - S = \frac{PC}{1+PC},$$

called the *complementary sensitivity function*.

Various performance specifications could be made using weighted versions of the transfer functions above. Note that a performance spec with weight $W$ on $PS$ is equivalent to the weight $WP$ on $S$. Similarly, a weight $W$ on $CS = T/P$ is equivalent to the weight $W/P$ on $T$. Thus performance specs that involve $e$ result in weights on $S$ and performance specs on $u$ result in weights on $T$. Essentially all problems in this book boil down to weighting $S$ or $T$ or some combination, and the tradeoff between making $S$ small and making $T$ small is the main issue in design.

## Exercises

1. Consider the unity-feedback system $[F(s) = 1]$. The definition of internal stability is that all nine closed-loop transfer functions should be stable. In the unity-feedback case, it actually suffices to check only two of the nine. Which two?

2. In this problem and the next, there is a mixture of the time and frequency domains, so the ˆ -convention is in force.

   Let

   $$\hat{P}(s) = \frac{1}{10s+1}, \quad \hat{C}(s) = k, \quad \hat{F}(s) = 1.$$

Find the least positive gain $k$ so that the following are all true:

(a) The feedback system is internally stable.

(b) $|e(\infty)| \leq 0.1$ when $r(t)$ is the unit step and $n = d = 0$.

(c) $\|y\|_\infty \leq 0.1$ for all $d(t)$ such that $\|d\|_2 \leq 1$ when $r = n = 0$.

3. For the setup in Figure 3.4, take $r = n = 0$, $d(t) = \sin(\omega t)1(t)$. Prove that if the feedback system is internally stable, then $y(t) \to 0$ as $t \to \infty$ iff either $\hat{P}$ has a zero at $s = j\omega$ or $\hat{C}$ has a pole at $s = j\omega$.

4. Consider the feedback system with plant $P$ and sensor $F$. Assume that $P$ is strictly proper and $F$ is proper. Find conditions on $P$ and $F$ for the existence of a proper controller so that

   The feedback system is internally stable.

   $y(t) - r(t) \to 0$ when $r$ is a unit step.

   $y(t) \to 0$ when $d$ is a sinusoid of frequency 100 rad/s.

## Notes and References

The material in Sections 3.1 to 3.3 is quite standard. However, Section 3.4 reflects the more recent viewpoint of Zames (1981), who formulated the problem of optimizing $W_1S$ with respect to the $\infty$-norm, stressing the role of the weight $W_1$. Additional motivation for this problem is offered in Zames and Francis (1983).

# Chapter 4

# Uncertainty and Robustness

No mathematical system can exactly model a physical system. For this reason we must be aware of how modeling errors might adversely affect the performance of a control system. This chapter begins with a treatment of various models of plant uncertainty. Then robust stability, stability in the face of plant uncertainty, is studied using the small-gain theorem. The final topic is robust performance, guaranteed tracking in the face of plant uncertainty.

## 4.1 Plant Uncertainty

The basic technique is to model the plant as belonging to a set $\mathcal{P}$. The reasons for doing this were presented in Chapter 1. Such a set can be either *structured* or *unstructured*.

For an example of a structured set consider the plant model

$$\frac{1}{s^2 + as + 1}.$$

This is a standard second-order transfer function with natural frequency 1 rad/s and damping ratio $a/2$—it could represent, for example, a mass-spring-damper setup or an R-L-C circuit. Suppose that the constant $a$ is known only to the extent that it lies in some interval $[a_{\min}, a_{\max}]$. Then the plant belongs to the structured set

$$\mathcal{P} = \left\{ \frac{1}{s^2 + as + 1} : a_{\min} \leq a \leq a_{\max} \right\}.$$

Thus one type of structured set is parametrized by a finite number of scalar parameters (one parameter, $a$, in this example). Another type of structured uncertainty is a discrete set of plants, not necessarily parametrized explicitly.

For us, unstructured sets are more important, for two reasons. First, we believe that all models used in feedback design should include some unstructured uncertainty to cover unmodeled dynamics, particularly at high frequency. Other types of uncertainty, though important, may or may not arise naturally in a given problem. Second, for a specific type of unstructured uncertainty, disk uncertainty, we can develop simple, general analysis methods. Thus the basic starting point for an unstructured set is that of disk-like uncertainty. In what follows, multiplicative disk uncertainty is chosen for detailed study. This is only one type of unstructured perturbation. The important point is that we use disk uncertainty instead of a more complicated description. We do this because it greatly simplifies our analysis and lets us say some fairly precise things. The price we pay is conservativeness.

### Multiplicative Perturbation

Suppose that the nominal plant transfer function is $P$ and consider perturbed plant transfer functions of the form $\tilde{P} = (1 + \Delta W_2)P$. Here $W_2$ is a fixed stable transfer function, the weight, and $\Delta$ is a variable stable transfer function satisfying $\|\Delta\|_\infty < 1$. Furthermore, it is assumed that no unstable poles of $P$ are canceled in forming $\tilde{P}$. (Thus, $P$ and $\tilde{P}$ have the same unstable poles.) Such a perturbation $\Delta$ is said to be *allowable*.

The idea behind this uncertainty model is that $\Delta W_2$ is the normalized plant perturbation away from 1:

$$\frac{\tilde{P}}{P} - 1 = \Delta W_2.$$

Hence if $\|\Delta\|_\infty \leq 1$, then

$$\left| \frac{\tilde{P}(j\omega)}{P(j\omega)} - 1 \right| \leq |W_2(j\omega)|, \qquad \forall \omega,$$

so $|W_2(j\omega)|$ provides the uncertainty profile. This inequality describes a disk in the complex plane: At each frequency the point $\tilde{P}/P$ lies in the disk with center 1, radius $|W_2|$. Typically, $|W_2(j\omega)|$ is a (roughly) increasing function of $\omega$: Uncertainty increases with increasing frequency. The main purpose of $\Delta$ is to account for phase uncertainty and to act as a scaling factor on the magnitude of the perturbation (i.e., $|\Delta|$ varies between 0 and 1).

Thus, this uncertainty model is characterized by a nominal plant $P$ together with a weighting function $W_2$. How does one get the weighting function $W_2$ in practice? This is illustrated by a few examples.

**Example 1** Suppose that the plant is stable and its transfer function is arrived at by means of frequency-response experiments: Magnitude and phase are measured at a number of frequencies, $\omega_i, i = 1, \ldots, m$, and this experiment is repeated several, say $n$, times. Let the magnitude-phase measurement for frequency $\omega_i$ and experiment $k$ be denoted $(M_{ik}, \phi_{ik})$. Based on these data select nominal magnitude-phase pairs $(M_i, \phi_i)$ for each frequency $\omega_i$, and fit a nominal transfer function $P(s)$ to these data. Then fit a weighting function $W_2(s)$ so that

$$\left| \frac{M_{ik} e^{j\phi_{ik}}}{M_i e^{j\phi_i}} - 1 \right| \leq |W_2(j\omega_i)|, \quad i = 1, \ldots, m; \; k = 1, \ldots, n.$$

**Example 2** Assume that the nominal plant transfer function is a double integrator:

$$P(s) = \frac{1}{s^2}.$$

For example, a dc motor with negligible viscous damping could have such a transfer function. You can think of other physical systems with only inertia, no damping. Suppose that a more detailed model has a time delay, yielding the transfer function

$$\tilde{P}(s) = e^{-\tau s} \frac{1}{s^2},$$

and suppose that the time delay is known only to the extent that it lies in the interval $0 \leq \tau \leq 0.1$. This time-delay factor $\exp(-\tau s)$ can be treated as a multiplicative perturbation of the nominal plant by embedding $\tilde{P}$ in the family

$$\{(1 + \Delta W_2)P : \|\Delta\|_\infty \leq 1\}.$$

To do this, the weight $W_2$ should be chosen so that the normalized perturbation satisfies

$$\left|\frac{\tilde{P}(j\omega)}{P(j\omega)} - 1\right| \leq |W_2(j\omega)|, \quad \forall \omega, \tau,$$

that is,

$$\left|e^{-\tau j\omega} - 1\right| \leq |W_2(j\omega)|, \quad \forall \omega, \tau.$$

A little time with Bode magnitude plots shows that a suitable first-order weight is

$$W_2(s) = \frac{0.21s}{0.1s + 1}.$$

Figure 4.1 is the Bode magnitude plot of this $W_2$ and $\exp(-\tau s) - 1$ for $\tau = 0.1$, the worst value.

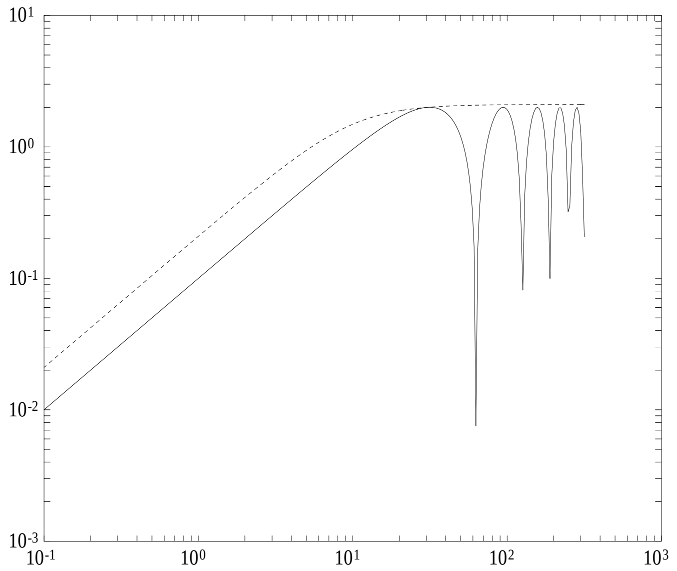


Figure 4.1: Bode plots of $W_2$ (dash) and $\exp(-0.1s) - 1$ (solid).

To get a feeling for how conservative this is, compare at a few frequencies $\omega$ the actual uncertainty set

$$\left\{\frac{\tilde{P}(j\omega)}{P(j\omega)} : 0 \leq \tau \leq 0.1\right\} = \left\{e^{-\tau j\omega} : 0 \leq \tau \leq 0.1\right\}$$

with the covering disk

$$\{s : |s - 1| \leq |W_2(j\omega)|\}.$$

**Example 3** Suppose that the plant transfer function is

$$\tilde{P}(s) = \frac{k}{s - 2},$$

where the gain $k$ is uncertain but is known to lie in the interval $[0.1, 10]$. This plant too can be embedded in a family consisting of multiplicative perturbations of a nominal plant

$$P(s) = \frac{k_0}{s-2}.$$

The weight $W_2$ must satisfy

$$\left| \frac{\tilde{P}(j\omega)}{P(j\omega)} - 1 \right| \leq |W_2(j\omega)|, \quad \forall \omega, k,$$

that is,

$$\max_{0.1 \leq k \leq 10} \left| \frac{k}{k_0} - 1 \right| \leq |W_2(j\omega)|, \quad \forall \omega.$$

The left-hand side is minimized by $k_0 = 5.05$, for which the left-hand side equals $4.95/5.05$. In this way we get the nominal plant

$$P(s) = \frac{5.05}{s-2}$$

and constant weight $W_2(s) = 4.95/5.05$.

The multiplicative perturbation model is not suitable for every application because the disk covering the uncertainty set is sometimes too coarse an approximation. In this case a controller designed for the multiplicative uncertainty model would probably be too conservative for the original uncertainty model.

The discussion above illustrates an important point. In modeling a plant we may arrive at a certain plant set. This set may be too awkward to cope with mathematically, so we may embed it in a larger set that is easier to handle. Conceivably, the achievable performance for the larger set may not be as good as the achievable performance for the smaller; that is, there may exist—even though we cannot find it—a controller that is better for the smaller set than the controller we design for the larger set. In this sense the latter controller is *conservative* for the smaller set.

In this book we stick with plant uncertainty that is disk-like. It will be conservative for some problems, but the payoff is that we obtain some very nice theoretical results. The resulting theory is remarkably practical as well.

## Other Perturbations

Other uncertainty models are possible besides multiplicative perturbations, as illustrated by the following example.

**Example 4** As at the start of this section, consider the family of plant transfer functions

$$\frac{1}{s^2 + as + 1}, \quad 0.4 \leq a \leq 0.8.$$

Thus

$$a = 0.6 + 0.2\Delta, \quad -1 \leq \Delta \leq 1,$$

so the family can be expressed as

$$\frac{P(s)}{1 + \Delta W_2(s) P(s)}, \quad -1 \leq \Delta \leq 1,$$

where

$$P(s) := \frac{1}{s^2 + 0.6s + 1}, \quad W_2(s) := 0.2s.$$

Note that $P$ is the nominal plant transfer function for the value $a = 0.6$, the midpoint of the interval. The block diagram corresponding to this representation of the plant is shown in Figure 4.2. Thus

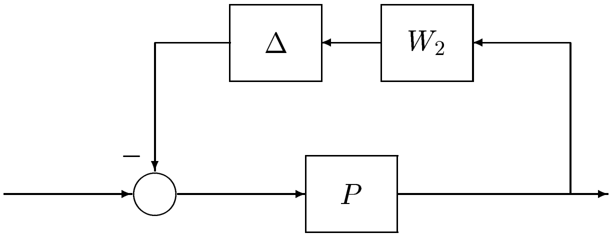


Figure 4.2: Example 4.

the original plant has been represented as a feedback uncertainty around a nominal plant.

The following list summarizes the common uncertainty models:

$$\begin{gathered}(1 + \Delta W_2)P \\ P + \Delta W_2 \\ P/(1 + \Delta W_2 P) \\ P/(1 + \Delta W_2)\end{gathered}$$

Appropriate assumptions would be made on $\Delta$ and $W_2$ in each case. Typically, we can relax the assumption that $\Delta$ be stable; but then the theorems to follow would be harder to prove.

## 4.2 Robust Stability

The notion of robustness can be described as follows. Suppose that the plant transfer function $P$ belongs to a set $\mathcal{P}$, as in the preceding section. Consider some characteristic of the feedback system, for example, that it is internally stable. A controller $C$ is *robust* with respect to this characteristic if this characteristic holds for every plant in $\mathcal{P}$. The notion of robustness therefore requires a controller, a set of plants, and some characteristic of the system. For us, the two most important variations of this notion are robust stability, treated in this section, and robust performance, treated in the next.

A controller $C$ provides *robust stability* if it provides internal stability for every plant in $\mathcal{P}$. We might like to have a test for robust stability, a test involving $C$ and $\mathcal{P}$. Or if $\mathcal{P}$ has an associated size, the maximum size such that $C$ stabilizes all of $\mathcal{P}$ might be a useful notion of stability margin.

The Nyquist plot gives information about stability margin. Note that the distance from the critical point -1 to the nearest point on the Nyquist plot of $L$ equals $1/\|S\|_\infty$:

$$\begin{aligned}\text{distance from -1 to Nyquist plot} &= \inf_\omega |-1 - L(j\omega)| \\ &= \inf_\omega |1 + L(j\omega)| \\ &= \left[\sup_\omega \frac{1}{|1 + L(j\omega)|}\right]^{-1}\end{aligned}$$

$$= \quad \|S\|_\infty^{-1}.$$

Thus if $\|S\|_\infty \gg 1$, the Nyquist plot comes close to the critical point, and the feedback system is nearly unstable. However, as a measure of stability margin this distance is not entirely adequate because it contains no frequency information. More precisely, if the nominal plant $P$ is perturbed to $\tilde{P}$, having the same number of unstable poles as has $P$ and satisfying the inequality

$$|\tilde{P}(j\omega)C(j\omega) - P(j\omega)C(j\omega)| < \|S\|_\infty^{-1}, \quad \forall\omega,$$

then internal stability is preserved (the number of encirclements of the critical point by the Nyquist plot does not change). But this is usually very conservative; for instance, larger perturbations could be allowed at frequencies where $P(j\omega)C(j\omega)$ is far from the critical point.

Better stability margins are obtained by taking explicit frequency-dependent perturbation models: for example, the multiplicative perturbation model, $\tilde{P} = (1 + \Delta W_2)P$. Fix a positive number $\beta$ and consider the family of plants

$$\{\tilde{P} : \Delta \text{ is stable and } \|\Delta\|_\infty \le \beta\}.$$

Now a controller $C$ that achieves internal stability for the nominal plant $P$ will stabilize this entire family if $\beta$ is small enough. Denote by $\beta_{\rm sup}$ the least upper bound on $\beta$ such that $C$ achieves internal stability for the entire family. Then $\beta_{\rm sup}$ is a stability margin (with respect to this uncertainty model). Analogous stability margins could be defined for the other uncertainty models.

We turn now to two classical measures of stability margin, gain and phase margin. Assume that the feedback system is internally stable with plant $P$ and controller $C$. Now perturb the plant to $kP$, with $k$ a positive real number. The *upper gain margin*, denoted $k_{\max}$, is the first value of $k$ greater than 1 when the feedback system is not internally stable; that is, $k_{\max}$ is the maximum number such that internal stability holds for $1 \le k < k_{\max}$. If there is no such number, then set $k_{\max} := \infty$. Similarly, the *lower gain margin*, $k_{\min}$, is the least nonnegative number such that internal stability holds for $k_{\min} < k \le 1$. These two numbers can be read off the Nyquist plot of $L$; for example, $-1/k_{\max}$ is the point where the Nyquist plot intersects the segment $(-1, 0)$ of the real axis, the closest point to $-1$ if there are several points of intersection.

Now perturb the plant to $e^{-j\phi}P$, with $\phi$ a positive real number. The *phase margin*, $\phi_{\max}$, is the maximum number (usually expressed in degrees) such that internal stability holds for $0 \le \phi < \phi_{\max}$. You can see that $\phi_{\max}$ is the angle through which the Nyquist plot must be rotated until it passes through the critical point, $-1$; or, in radians, $\phi_{\max}$ equals the arc length along the unit circle from the Nyquist plot to the critical point.

Thus gain and phase margins measure the distance from the critical point to the Nyquist plot in certain specific directions. Gain and phase margins have traditionally been important measures of stability robustness: if either is small, the system is close to instability. Notice, however, that the gain and phase margins can be relatively large and yet the Nyquist plot of $L$ can pass close to the critical point; that is, *simultaneous* small changes in gain and phase could cause internal instability. We return to these margins in Chapter 11.

Now we look at a typical robust stability test, one for the multiplicative perturbation model. Assume that the nominal feedback system (i.e., with $\Delta = 0$) is internally stable for controller $C$. Bring in again the complementary sensitivity function

$$T = 1 - S = \frac{L}{1+L} = \frac{PC}{1+PC}.$$

**Theorem 1** *(Multiplicative uncertainty model) C provides robust stability iff* $\|W_2T\|_\infty < 1$.

**Proof** ($\Leftarrow$) Assume that $\|W_2T\|_\infty < 1$. Construct the Nyquist plot of $L$, indenting $\mathcal{D}$ to the left around poles on the imaginary axis. Since the nominal feedback system is internally stable, we know this from the Nyquist criterion: The Nyquist plot of $L$ does not pass through -1 and its number of counterclockwise encirclements equals the number of poles of $P$ in Re$s \geq 0$ plus the number of poles of $C$ in Re$s \geq 0$.

Fix an allowable $\Delta$. Construct the Nyquist plot of $\tilde{P}C = (1+\Delta W_2)L$. No additional indentations are required since $\Delta W_2$ introduces no additional imaginary axis poles. We have to show that the Nyquist plot of $(1+\Delta W_2)L$ does not pass through -1 and its number of counterclockwise encirclements equals the number of poles of $(1+\Delta W_2)P$ in Re $s \geq 0$ plus the number of poles of $C$ in Re $s \geq 0$; equivalently, the Nyquist plot of $(1+\Delta W_2)L$ does not pass through -1 and encircles it exactly as many times as does the Nyquist plot of $L$. We must show, in other words, that the perturbation does not change the number of encirclements.

The key equation is

$$1 + (1+\Delta W_2)L = (1+L)(1+\Delta W_2 T). \tag{4.1}$$

Since

$$\|\Delta W_2 T\|_\infty \leq \|W_2T\|_\infty < 1,$$

the point $1+\Delta W_2T$ always lies in some closed disk with center 1, radius $< 1$, for all points $s$ on $\mathcal{D}$. Thus from (4.1), as $s$ goes once around $\mathcal{D}$, the net change in the angle of $1+(1+\Delta W_2)L$ equals the net change in the angle of $1+L$. This gives the desired result.

($\Rightarrow$) Suppose that $\|W_2T\|_\infty \geq 1$. We will construct an allowable $\Delta$ that destabilizes the feedback system. Since $T$ is strictly proper, at some frequency $\omega$,

$$|W_2(j\omega)T(j\omega)| = 1. \tag{4.2}$$

Suppose that $\omega = 0$. Then $W_2(0)T(0)$ is a real number, either $+1$ or $-1$. If $\Delta = -W_2(0)T(0)$, then $\Delta$ is allowable and

$$1 + \Delta W_2(0)T(0) = 0.$$

From (4.1) the Nyquist plot of $(1+\Delta W_2)L$ passes through the critical point, so the perturbed feedback system is not internally stable.

If $\omega > 0$, constructing an admissible $\Delta$ takes a little more work; the details are omitted. ■

The theorem can be used effectively to find the stability margin $\beta_{\text{sup}}$ defined previously. The simple scaling technique

$$\begin{aligned}\{\tilde{P} = (1+\Delta W_2)P : \|\Delta\|_\infty \leq \beta\} &= \{\tilde{P} = (1+\beta^{-1}\Delta\beta W_2)P : \|\beta^{-1}\Delta\|_\infty \leq 1\} \\ &= \{\tilde{P} = (1+\Delta_1\beta W_2)P : \|\Delta_1\|_\infty \leq 1\}\end{aligned}$$

together with the theorem shows that

$$\beta_{\text{sup}} = \sup\{\beta : \|\beta W_2 T\|_\infty < 1\} = 1/\|W_2T\|_\infty.$$

The condition $\|W_2T\|_\infty < 1$ also has a nice graphical interpretation. Note that

$$\begin{aligned}\|W_2T\|_\infty < 1 \quad &\Leftrightarrow \quad \left|\frac{W_2(j\omega)L(j\omega)}{1+L(j\omega)}\right| < 1, \quad \forall\omega \\ &\Leftrightarrow \quad |W_2(j\omega)L(j\omega)| < |1+L(j\omega)|, \quad \forall\omega.\end{aligned}$$

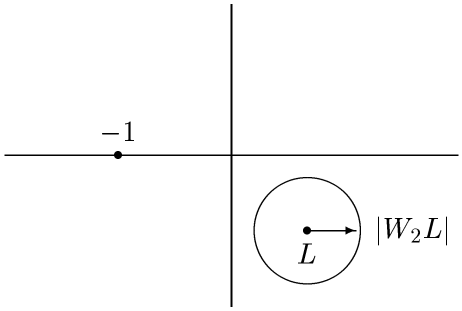


Figure 4.3: Robust stability graphically.

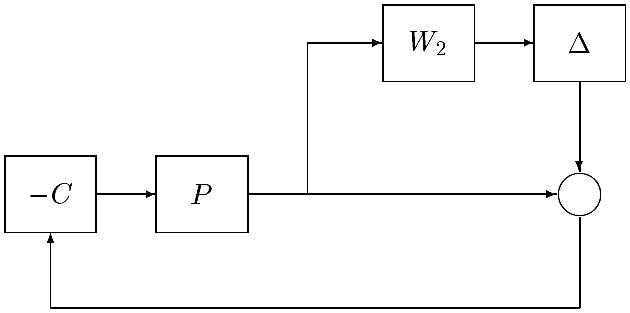


Figure 4.4: Perturbed feedback system.

The last inequality says that at every frequency, the critical point, -1, lies outside the disk of center $L(j\omega)$, radius $|W_2(j\omega)L(j\omega)|$ (Figure 4.3).

There is a simple way to see the relevance of the condition $\|W_2T\|_\infty < 1$. First, draw the block diagram of the perturbed feedback system, but ignoring inputs (Figure 4.4). The transfer function from the output of $\Delta$ around to the input of $\Delta$ equals $-W_2T$, so the block diagram collapses to the configuration shown in Figure 4.5. The maximum loop gain in Figure 4.5 equals $\|-\Delta W_2T\|_\infty$,

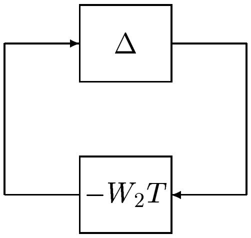


Figure 4.5: Collapsed block diagram.

which is $< 1$ for all allowable $\Delta$s iff the small-gain condition $\|W_2T\|_\infty < 1$ holds.

The foregoing discussion is related to the *small-gain theorem*, a special case of which is this: If $L$ is stable and $\|L\|_\infty < 1$, then $(1+L)^{-1}$ is stable too. An easy proof uses the Nyquist criterion.

### Summary of Robust Stability Tests

Table 4.1 summarizes the robust stability tests for the other uncertainty models.

| Perturbation | Condition |
|---|---|
| $(1+\Delta W_2)P$ | $\|W_2T\|_\infty < 1$ |
| $P+\Delta W_2$ | $\|W_2CS\|_\infty < 1$ |
| $P/(1+\Delta W_2 P)$ | $\|W_2PS\|_\infty < 1$ |
| $P/(1+\Delta W_2)$ | $\|W_2S\|_\infty < 1$ |

Table 4.1

Note that we get the same four transfer functions—$T$, $CS$, $PS$, $S$—as we did in Section 3.4. This should not be too surprising since (up to sign) these are the only closed-loop transfer functions for a unity feedback SISO system.

## 4.3 Robust Performance

Now we look into performance of the perturbed plant. Suppose that the plant transfer function belongs to a set $\mathcal{P}$. The general notion of *robust performance* is that internal stability and performance, of a specified type, should hold for all plants in $\mathcal{P}$. Again we focus on multiplicative perturbations.

Recall that when the nominal feedback system is internally stable, the *nominal performance* condition is $\|W_1S\|_\infty < 1$ and the *robust stability* condition is $\|W_2T\|_\infty < 1$. If $P$ is perturbed to $(1+\Delta W_2)P$, $S$ is perturbed to

$$\frac{1}{1+(1+\Delta W_2)L} = \frac{S}{1+\Delta W_2 T}.$$

Clearly, the *robust performance* condition should therefore be

$$\|W_2T\|_\infty < 1 \text{ and } \left\|\frac{W_1S}{1+\Delta W_2 T}\right\|_\infty < 1, \quad \forall\Delta.$$

Here $\Delta$ must be allowable. The next theorem gives a test for robust performance in terms of the function

$$s \mapsto |W_1(s)S(s)| + |W_2(s)T(s)|,$$

which is denoted $|W_1S| + |W_2T|$.

**Theorem 2** *A necessary and sufficient condition for robust performance is*

$$\||W_1S| + |W_2T|\|_\infty < 1. \tag{4.3}$$

**Proof** ($\Leftarrow$) Assume (4.3), or equivalently,

$$\|W_2T\|_\infty \text{ and } \left\|\frac{W_1S}{1-|W_2T|}\right\|_\infty < 1. \tag{4.4}$$

Fix $\Delta$. In what follows, functions are evaluated at an arbitrary point $j\omega$, but this is suppressed to simplify notation. We have

$$1 = |1 + \Delta W_2 T - \Delta W_2 T| \le |1 + \Delta W_2 T| + |W_2 T|$$

and therefore

$$1 - |W_2 T| \le |1 + \Delta W_2 T|.$$

This implies that

$$\left\| \frac{W_1 S}{1 - |W_2 T|} \right\|_\infty \ge \left\| \frac{W_1 S}{1 + \Delta W_2 T} \right\|_\infty .$$

This and (4.4) yield

$$\left\| \frac{W_1 S}{1 + \Delta W_2 T} \right\|_\infty < 1.$$

($\Rightarrow$) Assume that

$$\|W_2 T\|_\infty < 1 \text{ and } \left\| \frac{W_1 S}{1 + \Delta W_2 T} \right\|_\infty < 1, \quad \forall \Delta. \tag{4.5}$$

Pick a frequency $\omega$ where

$$\frac{|W_1 S|}{1 - |W_2 T|}$$

is maximum. Now pick $\Delta$ so that

$$1 - |W_2 T| = |1 + \Delta W_2 T|.$$

The idea here is that $\Delta(j\omega)$ should rotate $W_2(j\omega)T(j\omega)$ so that $\Delta(j\omega)W_2(j\omega)T(j\omega)$ is negative real. The details of how to construct such an allowable $\Delta$ are omitted. Now we have

$$\begin{aligned} \left\| \frac{W_1 S}{1 - |W_2 T|} \right\|_\infty &= \frac{|W_1 S|}{1 - |W_2 T|} \\ &= \frac{|W_1 S|}{|1 + \Delta W_2 T|} \\ &\le \left\| \frac{W_1 S}{1 + \Delta W_2 T} \right\|_\infty . \end{aligned}$$

So from this and (4.5) there follows (4.4). ■

Test (4.3) also has a nice graphical interpretation. For each frequency $\omega$, construct two closed disks: one with center $-1$, radius $|W_1(j\omega)|$; the other with center $L(j\omega)$, radius $|W_2(j\omega)L(j\omega)|$. Then (4.3) holds iff for each $\omega$ these two disks are disjoint (Figure 4.6).

The robust performance condition says that the robust performance level 1 is achieved. More generally, let's say that robust performance level $\alpha$ is achieved if

$$\|W_2 T\|_\infty < 1 \text{ and } \left\| \frac{W_1 S}{1 + \Delta W_2 T} \right\|_\infty < \alpha, \quad \forall \Delta.$$

Noting that at every frequency

$$\max_{|\Delta| \le 1} \left| \frac{W_1 S}{1 + \Delta W_2 T} \right| = \frac{|W_1 S|}{1 - |W_2 T|}$$

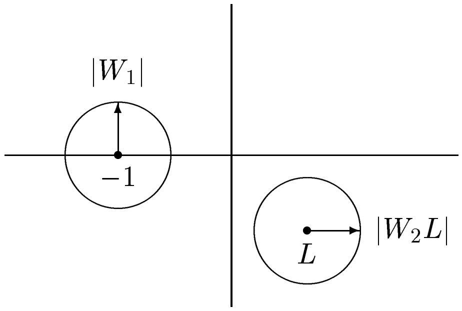


Figure 4.6: Robust performance graphically.

we get that the minimum $\alpha$ equals

$$\left\| \frac{W_1 S}{1 - |W_2 T|} \right\|_\infty . \tag{4.6}$$

Alternatively, we may wish to know how large the uncertainty can be while the robust performance condition holds. To do this, we scale the uncertainty level, that is, we allow $\Delta$ to satisfy $\|\Delta\|_\infty < \beta$. Application of Theorem 1 shows that internal stability is robust iff $\|\beta W_2 T\|_\infty < 1$. Let's say that the uncertainty level $\beta$ is permissible if

$$\|\beta W_2 T\|_\infty < 1 \text{ and } \left\| \frac{W_1 S}{1 + \Delta W_2 T} \right\|_\infty < 1, \quad \forall \Delta.$$

Again, noting that

$$\max_{|\Delta| \le 1} \left| \frac{W_1 S}{1 + \beta \Delta W_2 T} \right| = \frac{|W_1 S|}{1 - \beta |W_2 T|},$$

we get that the maximum $\beta$ equals

$$\left\| \frac{W_2 T}{1 - |W_1 S|} \right\|_\infty^{-1} .$$

Now we turn briefly to some related problems.

### Robust Stability for Multiple Perturbations

Suppose that a nominal plant $P$ is perturbed to

$$\tilde{P} = P \frac{1 + \Delta_2 W_2}{1 + \Delta_1 W_1}$$

with $W_1$, $W_2$ both stable and $\Delta_1$, $\Delta_2$ both admissible. The robust stability condition is

$$\| |W_1 S| + |W_2 T| \|_\infty < 1,$$

which is just the robust performance condition in Theorem 2. A sketch of the proof goes like this: From the fourth entry in Table 4.1, for fixed $\Delta_2$ the robust stability condition for varying $\Delta_1$ is

$$\left\| W_1 \frac{1}{1 + (1 + \Delta_2 W_2) L} \right\|_\infty < 1.$$

Then from Theorem 2 this holds for all admissible $\Delta_2$ iff

$$\| |W_1 S| + |W_2 T| \|_\infty < 1.$$

This illustrates a more general point: Robust performance with one perturbation is equivalent to robust stability with two perturbations, provided that performance is in terms of the $\infty$-norm and the second perturbation is chosen appropriately.

### Robust Command Response

Consider the block diagram shown in Figure 4.7. Shown are a plant $P$ and two controller compo-

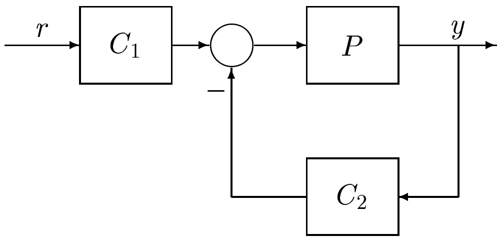


Figure 4.7: Two-degree-of-freedom controller.

nents, $C_1$ and $C_2$. This is known as a two-degree-of-freedom controller because the plant input is allowed to be a function of the two signals $r$ and $y$ independently, not just $r - y$. We will not go into details about such controllers or about the appropriate definition of internal stability.

Define

$$S := \frac{1}{1 + PC_2}, \quad T := 1 - S.$$

Then the transfer function from $r$ to $y$, denoted $T_{yr}$, is

$$T_{yr} = PSC_1.$$

Let $M$ be a transfer function representing a model that we want the foregoing system to emulate. Denote by $e$ the difference between $y$ and the output of $M$. The error transfer function, that from $r$ to $e$, is

$$T_{er} = T_{yr} - M = PSC_1 - M.$$

The ideal choice for $C_1$, the one making $T_{er} = 0$, would therefore be

$$C_1 = \frac{M}{PS}.$$

This choice may violate the internal stability constraint, but let's suppose that in order to continue that it does not (this places some limitations on $M$).

Consider now a multiplicative perturbation of the plant: $P$ becomes $\tilde{P} = (1 + \Delta W_2)P$, $\Delta$ admissible. Then $T_{er}$ becomes

$$\begin{aligned}
\tilde{T}_{er} &= \frac{\tilde{P}C_1}{1 + \tilde{P}C_2} - M \\
&= \frac{\tilde{P}}{1 + \tilde{P}C_2} \frac{M}{PS} - M \\
&= \frac{\Delta W_2 M S}{1 + \Delta W_2 T} \text{ (after some algebra).}
\end{aligned}$$

Defining $W_1 := W_2 M$, we find that the maximum $\infty$-norm of the error transfer function, over all admissible $\Delta$, is

$$\max_{\Delta} \|\tilde{T}_{ec}\|_\infty = \left\| \frac{W_1 S}{1 - |W_2 T|} \right\|_\infty .$$

The right-hand side we have already seen in (4.6).

Note that we convert the problem of making the closed-loop response from $r$ to $y$ match some desired response by subtracting off that desired response and forming an error signal $e$ which we seek to keep small. In some treatments of the command response problem, the performance specification is taken to be: make $|T_{yr}|$ close to a desired model. The problem with this specification is that two transfer functions can be close in magnitude but differ substantially in phase. Surprisingly, this can occur even when both transfer functions are minimum phase. The interested reader may want to investigate this further using the gain-phase relation developed in Chapter 7.

## 4.4 Robust Performance More Generally

Theorem 2 gives the robust performance test under the following conditions:

| | |
|---|---|
| Perturbation model: | $(1 + \Delta W_2)P$ |
| Nominal performance condition: | $\|W_1 S\|_\infty < 1$ |

Table 4.2 gives tests for the four uncertainty models and two nominal performance conditions.

| | Nominal Performance Condition | |
|---|---|---|
| Perturbation | $\|W_1 S\|_\infty < 1$ | $\|W_1 T\|_\infty < 1$ |
| $(1 + \Delta W_2)P$ | $\||W_1 S| + |W_2 T|\|_\infty < 1$ | messy |
| $P + W_2 \Delta$ | $\||W_1 S| + |W_2 C S|\|_\infty < 1$ | messy |
| $P/(1 + \Delta W_2 P)$ | messy | $\||W_1 T| + |W_2 P S|\|_\infty < 1$ |
| $P/(1 + \Delta W_2)$ | messy | $\||W_1 T| + |W_2 S|\|_\infty < 1$ |

Table 4.2

The entries marked *messy* are just that. The difficulty is the way in which $\Delta$ enters. For example, consider the case where

| | |
|---|---|
| Perturbation model: | $(1 + \Delta W_2)P$ |
| Nominal performance condition: | $\|W_1 T\|_\infty < 1$ |

The perturbed $T$ is

$$\frac{(1 + \Delta W_2)PC}{1 + (1 + \Delta W_2)PC} = \frac{(1 + \Delta W_2)T}{1 + \Delta W_2 T},$$

so the perturbed performance condition is equivalent to

$$|W_1(1 + \Delta W_2)T| < |1 + \Delta W_2 T|, \quad \forall \omega.$$

Now for each fixed $\omega$

$$|W_1(1 + \Delta W_2)T| \le |W_1 T|(1 + |W_2|)$$

and

$$1 - |W_2T| \leq |1 + \Delta W_2 T|.$$

So a sufficient condition for robust performance is

$$\left\| \frac{W_1 T(1 + |W_2|)}{1 - |W_2 T|} \right\|_\infty < 1.$$

## 4.5 Conclusion

The nominal feedback system is assumed to be internally stable. Then the *nominal performance* condition is $\|W_1S\|_\infty < 1$ and the *robust stability* condition (with respect to multiplicative perturbations) is $\|W_2T\|_\infty < 1$.

The condition for simultaneously achieving nominal performance and robust stability is

$$\| \max\,(|W_1S|, |W_2T|)\|_\infty < 1. \tag{4.7}$$

The *robust performance* condition is

$$\|W_2T\|_\infty < 1 \text{ and } \left\| \frac{W_1 S}{1 + \Delta W_2 T} \right\|_\infty < 1, \quad \forall \Delta$$

and the test for this is

$$\||W_1S| + |W_2T|\|_\infty < 1. \tag{4.8}$$

Since

$$\max\,(|W_1S|, |W_2T|) \leq |W_1S| + |W_2T| \leq 2 \max\,(|W_1S|, |W_2T|) \tag{4.9}$$

conditions (4.7) and (4.8) are not too far apart. For instance, if nominal performance and robust stability are obtained with a safety factor of 2, that is,

$$\|W_1S\|_\infty < 1/2, \quad \|W_2T\|_\infty < 1/2,$$

then robust performance is automatically obtained.

A compromise condition, which we shall treat in Chapters 8 and 12, is

$$\|(|W_1S|^2 + |W_2T|^2)^{1/2}\|_\infty < 1. \tag{4.10}$$

Simple plane geometry shows that

$$\max\,(|W_1S|, |W_2T|) \leq (|W_1S|^2 + |W_2T|^2)^{1/2} \leq |W_1S| + |W_2T| \tag{4.11}$$

and

$$\frac{1}{\sqrt{2}}(|W_1S| + |W_2T|) \leq (|W_1S|^2 + |W_2T|^2)^{1/2} \leq \sqrt{2} \max\,(|W_1S|, |W_2T|). \tag{4.12}$$

Thus (4.10) is a reasonable approximation to both (4.7) and (4.8).

To elaborate on this point, let's consider

$$x = \begin{pmatrix} x_1 \\ x_2 \end{pmatrix} = \begin{pmatrix} |W_1S| \\ |W_2T| \end{pmatrix}$$

as a vector in $\mathbb{R}^2$. Then (4.7), (4.8), and (4.10) correspond, respectively, to the three different norms

$$\max\,(|x_1|, |x_2|), \quad |x_1| + |x_2|, \quad (|x_1|^2 + |x_2|^2)^{1/2}.$$

The third is the Euclidean norm and is the most tractable. The point being made here is that choice of these spatial norms is not crucial: The tradeoffs between $|W_1S|$ and $|W_2T|$ inherent in control problems mean that although the norms may differ by as much as a factor of 2, the actual solutions one gets by using the different norms are not very different.

## Exercises

1. Consider a unity-feedback system. True or false: If a controller internally stabilizes two plants, they have the same number of poles in Re$s \geq 0$.

2. Unity-feedback problem. Let $P_\alpha(s)$ be a plant depending on a real parameter $\alpha$. Suppose that the poles of $P_\alpha$ move continuously as $\alpha$ varies over the interval $[0, 1]$. True or false: If a controller internally stabilizes $P_\alpha$ for every $\alpha$ in $[0, 1]$, then $P_\alpha$ has the same number of poles in Re $s \geq 0$ for every $\alpha$ in $[0, 1]$.

3. For the unity-feedback system with $P(s) = k/s$, does there exist a proper controller $C(s)$ such that the feedback system is internally stable for both $k = +1$ and $k = -1$?

4. Suppose that
$$P(s) = \frac{\omega_n^2}{s(s + 2\zeta\omega_n)}, \quad C(s) = 1$$
with $\omega_n, \zeta > 0$. Note that the characteristic polynomial is the standard second-order one. Find the phase margin as a function of $\zeta$. Sketch the graph of this function.

5. Consider the unity-feedback system with
$$P(s) = \frac{1}{(s+1)(s+\alpha)}, \quad C(s) = \frac{1}{s}.$$
For what range of $\alpha$ (a real number) is the feedback system internally stable? Find the upper and lower gain margins as functions of $\alpha$.

6. This problem studies robust stability for real parameter variations. Consider the unity-feedback system with $C(s) = 10$ and plant
$$\frac{1}{s - a},$$
where $a$ is real.

   (a) Find the range of $a$ for the feedback system to be internally stable.

   (b) For $a = 0$ the plant is $P(s) = 1/s$. Regarding $a$ as a perturbation, we can write the plant as
   $$\tilde{P} = \frac{P}{1 + \Delta W_2 P}$$
   with $W_2(s) = -a$. Then $\tilde{P}$ equals the true plant when $\Delta(s) = 1$. Apply robust stability theory to see when the feedback system with plant $\tilde{P}$ is internally stable for all $\|\Delta\|_\infty \leq 1$. You will get a smaller range for $a$ than in part (a).

   (c) Repeat with the nominal plant $P(s) = 1/(s + 100)$.

7. This problem concerns robust stability of the unity-feedback system. Suppose that $P$ and $C$ are nominal transfer functions for which the feedback system is internally stable. Instead of allowing perturbations in just $P$, this problem allows perturbations in $C$ too. Suppose that $P$ may be perturbed to
$$(1 + \Delta_1 W)P$$

and $C$ may be perturbed to

$$(1 + \Delta_2 V)C.$$

The transfer functions $W$ and $V$ are fixed, while $\Delta_1$ and $\Delta_2$ are variable transfer functions having $\infty$-norms no greater than 1. Making appropriate additional assumptions, find a sufficient condition, depending only on the four functions $P$, $C$, $W$, $V$, for robust stability. Prove sufficiency. (A weak sufficient condition is the goal; for example, the condition $W = V = 0$ would be too strong.)

8. Assume that the nominal plant transfer function is a double integrator,

$$P(s) = \frac{1}{s^2}.$$

The performance requirement is that the plant output should track reference inputs over the frequency range $[0, 1]$. The performance weight $W_1$ could therefore be chosen so that its magnitude is constant over this frequency range and then rolls off at higher frequencies. A common choice for $W_1$ is a Butterworth filter, which is maximally flat over its bandwidth. Choose a third-order Butterworth filter for $W_1$ with cutoff frequency 1 rad/s. Take the weight $W_2$ to be

$$W_2(s) = \frac{0.21s}{0.1s + 1}.$$

(a) Design a proper $C$ to achieve internal stability for the nominal plant.

(b) Check the robust stability condition $\|W_2 T\|_\infty < 1$. If this does not hold, redesign $C$ until it does. It is not necessary to get a $C$ that yields good performance.

(c) Compute the robust performance level $\alpha$ for your controller from (4.6).

9. Consider the class of perturbed plants of the form

$$\frac{P}{1 + \Delta W_2 P},$$

where $W_2$ is a fixed stable weighting function with $W_2 P$ strictly proper and $\Delta$ is a variable stable transfer function with $\|\Delta\|_\infty \le 1$. Assume that $C$ is a controller achieving internal stability for $P$. Prove that $C$ provides internal stability for the perturbed plant if $\|W_2 P S\|_\infty < 1$.

10. Suppose that the plant transfer function is

$$\tilde{P}(s) = [1 + \Delta(s) W_2(s)]\, P(s),$$

where

$$W_2(s) = \frac{2}{s + 10}, \quad P(s) = \frac{1}{s - 1},$$

and the stable perturbation $\Delta$ satisfies $\|\Delta\|_\infty \le 2$. Suppose that the controller is the pure gain $C(s) = k$. We want the feedback system to be internally stable for all such perturbations. Determine over what range of $k$ this is true.

## Notes and References

The basis for this chapter is Doyle and Stein (1981). This paper emphasized the importance of explicit uncertainty models such as multiplicative and additive. Theorem 1 is stated in that paper, but a complete proof is due to Chen and Desoer (1982). The sufficiency part of this theorem is a version of the small-gain theorem, due to Sandberg and Zames [see, e.g., Desoer and Vidyasagar (1975)].

# Chapter 5

# Stabilization

In this chapter we study the unity-feedback system with block diagram shown in Figure 5.1. Here

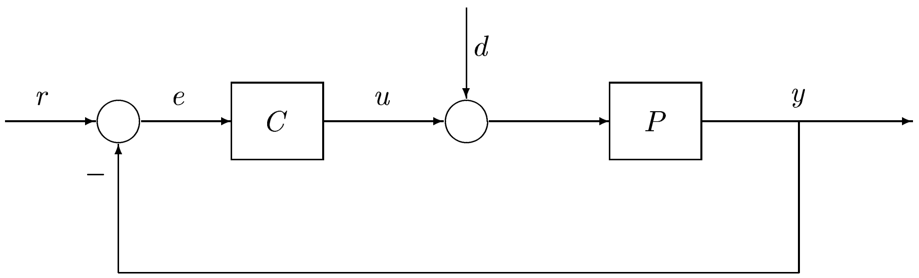


Figure 5.1: Unity-feedback system.

$P$ is strictly proper and $C$ is proper.

Most synthesis problems can be formulated in this way: Given $P$, design $C$ so that the feedback system (1) is internally stable, and (2) acquires some additional desired property; for example, the output $y$ asymptotically tracks a step input $r$. The method of solution is to parametrize all $C$s for which (1) is true, and then to see if there exists a parameter for which (2) holds. In this chapter such a parametrization is derived and then applied to two problems: achieving asymptotic performance specs and internal stabilization by a stable controller.

## 5.1 Controller Parametrization: Stable Plant

In this section we assume that $P$ is already stable, and we parametrize all $C$s for which the feedback system is internally stable. Introduce the symbol $\mathcal{S}$ for the family of all stable, proper, real-rational functions. Notice that $\mathcal{S}$ is closed under addition and multiplication: If $F, G \in \mathcal{S}$, then $F + G, FG \in \mathcal{S}$. Also, $1 \in \mathcal{S}$. (Thus $\mathcal{S}$ is a commutative ring with identity.)

**Theorem 1** *Assume that $P \in \mathcal{S}$. The set of all $C$s for which the feedback system is internally stable equals*

$$\left\{ \frac{Q}{1 - PQ} : Q \in \mathcal{S} \right\}.$$

**Proof** ($\subset$) Suppose that $C$ achieves internal stability. Let $Q$ denote the transfer function from $r$ to $u$, that is,

$$Q := \frac{C}{1+PC}.$$

Then $Q \in \mathcal{S}$ and

$$C = \frac{Q}{1-PQ}.$$

($\supset$) Conversely, suppose that $Q \in \mathcal{S}$ and define

$$C := \frac{Q}{1-PQ}. \tag{5.1}$$

According to the definition in Section 3.2, the feedback system is internally stable iff the nine transfer functions

$$\frac{1}{1+PC}\begin{bmatrix} 1 & -P & -1 \\ C & 1 & -C \\ PC & P & 1 \end{bmatrix}$$

all are stable and proper. After substitution from (5.1) and clearing of fractions, this matrix becomes

$$\begin{bmatrix} 1-PQ & -P(1-PQ) & -(1-PQ) \\ Q & 1-PQ & -Q \\ PQ & P(1-PQ) & 1-PQ \end{bmatrix}.$$

Clearly, these nine entries belong to $\mathcal{S}$. ■

Note that all nine transfer functions above are affine functions of the free parameter $Q$; that is, each is of the form $T_1 + T_2Q$ for some $T_1, T_2$ in $\mathcal{S}$. In particular the sensitivity and complementary sensitivity functions are

$$\begin{aligned} S &= 1-PQ, \\ T &= PQ. \end{aligned}$$

Let us look at a simple application. Suppose that we want to find a $C$ so that the feedback system is internally stable and $y$ asymptotically tracks a step $r$ (when $d=0$). Parametrize $C$ as in the theorem. Then $y$ asymptotically tracks a step iff the transfer function from $r$ to $e$ (i.e., $S$) has a zero at $s=0$, that is,

$$P(0)Q(0) = 1.$$

This equation has a solution $Q$ in $\mathcal{S}$ iff $P(0) \neq 0$. Conclusion: The problem has a solution iff $P(0) \neq 0$; when this holds, the set of all solutions is

$$\left\{ C = \frac{Q}{1-PQ} : Q \in \mathcal{S}, Q(0) = \frac{1}{P(0)} \right\}.$$

Observe that $Q$ inverts $P$ at dc. Also, you can check that a controller of the latter form has a pole at $s=0$, as it must by Theorem 3 of Chapter 3.

**Example** For the plant

$$P(s) = \frac{1}{(s+1)(s+2)}$$

suppose that it is desired to find an internally stabilizing controller so that $y$ asymptotically tracks a ramp $r$. Parametrize $C$ as in the theorem. The transfer function $S$ from $r$ to $e$ must have (at least) two zeros at $s = 0$, where $r$ has two poles. Let us take

$$Q(s) = \frac{as+b}{s+1}.$$

This belongs to $\mathcal{S}$ and has two variables, $a$ and $b$, for the assignment of the two zeros of $S$. We have

$$\begin{aligned} S(s) &= 1 - \frac{as+b}{(s+1)^2(s+2)} \\ &= \frac{s^3+4s^2+(5-a)s+(2-b)}{(s+1)^2(s+2)}, \end{aligned}$$

so we should take $a = 5, b = 2$. This gives

$$\begin{aligned} Q(s) &= \frac{5s+2}{s+1}, \\ C(s) &= \frac{(5s+2)(s+1)(s+2)}{s^2(s+4)}. \end{aligned}$$

The controller is internally stabilizing and has two poles at $s = 0$.

## 5.2 Coprime Factorization

Now suppose that $P$ is not stable and we want to find an internally stabilizing $C$. We might try as follows. Write $P$ as the ratio of coprime polynomials,

$$P = \frac{N}{M}.$$

By Euclid's algorithm (reviewed below) we can get two other polynomials $X$, $Y$ satisfying the equation

$$NX + MY = 1.$$

Remembering Theorem 3.1 (the feedback system is internally stable iff the characteristic polynomial has no zeros in Re $s \geq 0$), we might try to make the left-hand side equal to the characteristic polynomial by setting

$$C = \frac{X}{Y}.$$

The trouble is that $Y$ may be 0; even if not, this $C$ may not be proper.

**Example 1** For $P(s) = 1/s$, we can take $N(s) = 1$, $M(s) = s$. One solution to the equation $NX + MY = 1$ is $X(s) = 1$, $Y(s) = 0$, for which $X/Y$ is undefined. Another solution is $X(s) = -s + 1$, $Y(s) = 1$, for which $X/Y$ is not proper.

The remedy is to arrange that $N$, $M$, $X$, $Y$ are all elements of $\mathcal{S}$ instead of polynomials. Two functions $N$ and $M$ in $\mathcal{S}$ are *coprime* if there exist two other functions $X$ and $Y$ also in $\mathcal{S}$ and satisfying the equation

$$NX + MY = 1.$$

Notice that for this equation to hold, $N$ and $M$ can have no common zeros in $\mathrm{Re}s \geq 0$ nor at the point $s = \infty$—if there were such a point $s_0$, there would follow

$$0 = N(s_0)X(s_0) + M(s_0)Y(s_0) \neq 1.$$

It can be proved that this condition is also sufficient for coprimeness.

Let $G$ be a real-rational transfer function. A representation of the form

$$G = \frac{N}{M}, \quad N, M \in \mathcal{S},$$

where $N$ and $M$ are coprime, is called a *coprime factorization* of $G$ over $\mathcal{S}$. The purpose of this section is to present a method for the construction of four functions in $\mathcal{S}$ satisfying the two equations

$$G = \frac{N}{M}, \quad NX + MY = 1.$$

The construction of $N$ and $M$ is easy.

**Example 2** Take $G(s) = 1/(s-1)$. To write $G = N/M$ with $N$ and $M$ in $\mathcal{S}$, simply divide the numerator and denominator polynomials, 1 and $s-1$, by a common polynomial with no zeros in $\mathrm{Re}s \geq 0$, say $(s+1)^k$:

$$\frac{1}{s-1} = \frac{N(s)}{M(s)}, \quad N(s) = \frac{1}{(s+1)^k}, \quad M(s) = \frac{s-1}{(s+1)^k}.$$

If the integer $k$ is greater than 1, then $N$ and $M$ are not coprime—they have a common zero at $s = \infty$. So

$$N(s) = \frac{1}{s+1}, \quad M(s) = \frac{s-1}{s+1}$$

suffice.

More generally, to get $N$ and $M$ we could divide the numerator and denominator polynomials of $G$ by $(s+1)^k$, where $k$ equals the maximum of their degrees. What is not so easy is to get the other two functions, $X$ and $Y$, and this is why we need Euclid's algorithm.

Euclid's algorithm computes the greatest common divisor of two given polynomials, say $n(\lambda)$ and $m(\lambda)$. When $n$ and $m$ are coprime, the algorithm can be used to compute polynomials $x(\lambda)$, $y(\lambda)$ satisfying

$$nx + my = 1.$$

**Procedure A:** Euclid's Algorithm

Input: polynomials $n$, $m$

Initialize: If it is not true that degree $(n) \geq$ degree $(m)$, interchange $n$ and $m$.

**Step 1** Divide $m$ into $n$ to get quotient $q_1$ and remainder $r_1$:

$$n = mq_1 + r_1,$$

$$\text{degree } r_1 < \text{ degree } m.$$

**Step 2** Divide $r_1$ into $m$ to get quotient $q_2$ and remainder $r_2$:

$$m = r_1 q_2 + r_2,$$

$$\text{degree } r_2 < \text{ degree } r_1.$$

**Step 3** Divide $r_2$ into $r_1$:

$$r_1 = r_2 q_3 + r_3,$$

$$\text{degree } r_3 < \text{ degree } r_2.$$

**Continue.**

Stop at Step $k$ when $r_k$ is a nonzero constant.

Then $x$, $y$ are obtained as illustrated by the following example for $k = 3$. The equations are

$$n = mq_1 + r_1,$$

$$m = r_1 q_2 + r_2,$$

$$r_1 = r_2 q_3 + r_3,$$

that is,

$$\begin{bmatrix} 1 & 0 & 0 \\ q_2 & 1 & 0 \\ -1 & q_3 & 1 \end{bmatrix} \begin{bmatrix} r_1 \\ r_2 \\ r_3 \end{bmatrix} = \begin{bmatrix} 1 & -q_1 \\ 0 & 1 \\ 0 & 0 \end{bmatrix} \begin{bmatrix} n \\ m \end{bmatrix}.$$

Solve for $r_3$ by, say, Gaussian elimination:

$$r_3 = (1 + q_2 q_3)n + [-q_3 - q_1(1 + q_2 q_3)]m.$$

Set

$$\begin{aligned} x &= \frac{1}{r_3}(1 + q_2 q_3), \\ y &= \frac{1}{r_3}[-q_3 - q_1(1 + q_2 q_3)]. \end{aligned}$$

**Example 3** The algorithm for $n(\lambda) = \lambda^2$, $m(\lambda) = 6\lambda^2 - 5\lambda + 1$ goes like this:

$$\begin{aligned} q_1(\lambda) &= \frac{1}{6}, \\ r_1(\lambda) &= \frac{5}{6}\lambda - \frac{1}{6}, \\ q_2(\lambda) &= \frac{36}{5}\lambda - \frac{114}{25}, \\ r_2(\lambda) &= \frac{6}{25}. \end{aligned}$$

Since $r_2$ is a nonzero constant, we stop after Step 2. Then the equations are

$$\begin{aligned} n &= mq_1 + r_1, \\ m &= r_1 q_2 + r_2, \end{aligned}$$

yielding

$$r_2 = (1 + q_1 q_2)m - q_2 n.$$

So we should take

$$x = -\frac{q_2}{r_2}, \quad y = \frac{1 + q_1 q_2}{r_2},$$

that is,

$$x(\lambda) = -30\lambda + 19, \quad y(\lambda) = 5\lambda + 1.$$

Next is a procedure for doing a coprime factorization of $G$. The main idea is to transform variables, $s \to \lambda$, so that polynomials in $\lambda$ yield functions in $\mathcal{S}$.

**Procedure B**

Input: $G$

**Step 1** If $G$ is stable, set $N = G$, $M = 1$, $X = 0$, $Y = 1$, and stop; else, continue.

**Step 2** Transform $G(s)$ to $\tilde{G}(\lambda)$ under the mapping $s = (1 - \lambda)/\lambda$. Write $\tilde{G}$ as a ratio of coprime polynomials:

$$\tilde{G}(\lambda) = \frac{n(\lambda)}{m(\lambda)}.$$

**Step 3** Using Euclid's algorithm, find polynomials $x(\lambda)$, $y(\lambda)$ such that

$$nx + my = 1.$$

**Step 4** Transform $n(\lambda)$, $m(\lambda)$, $x(\lambda)$, $y(\lambda)$ to $N(s)$, $M(s)$, $X(s)$, $Y(s)$ under the mapping $\lambda = 1/(s + 1)$.

The mapping used in this procedure is not unique; the only requirement is that polynomials $n$, and so on, map to $N$, and so on, in $\mathcal{S}$.

**Example 4** For

$$G(s) = \frac{1}{(s-1)(s-2)}$$

the algorithm gives

$$\begin{aligned}
\tilde{G}(\lambda) &= \frac{\lambda^2}{6\lambda^2 - 5\lambda + 1}, \\
n(\lambda) &= \lambda^2, \\
m(\lambda) &= 6\lambda^2 - 5\lambda + 1, \\
x(\lambda) &= -30\lambda + 19, \\
y(\lambda) &= 5\lambda + 1 \quad \text{(from Example 3)}, \\
N(s) &= \frac{1}{(s+1)^2},
\end{aligned}$$

$$\begin{aligned} M(s) &= \frac{(s-1)(s-2)}{(s+1)^2}, \\ X(s) &= \frac{19s-11}{s+1}, \\ Y(s) &= \frac{s+6}{s+1}. \end{aligned}$$

## 5.3 Coprime Factorization by State-Space Methods (Optional)

This optional section presents a state-space procedure for computing a coprime factorization over $\mathcal{S}$ of a proper $G$. This procedure is more efficient than the polynomial method in the preceding section.

We start with a new data structure. Suppose that $A$, $B$, $C$, $D$ are real matrices of dimensions

$$n \times n, \quad n \times 1, \quad 1 \times n, \quad 1 \times 1.$$

The transfer function going along with this quartet is

$$D + C(sI - A)^{-1}B.$$

Note that the constant $D$ equals the value of the transfer function at $s = \infty$; if the transfer function is strictly proper, then $D = 0$. It is convenient to write

$$\left[\begin{array}{c|c} A & B \\ \hline C & D \end{array}\right]$$

instead of

$$D + C(sI - A)^{-1}B.$$

Beginning with a realization of $G$,

$$G(s) = \left[\begin{array}{c|c} A & B \\ \hline C & D \end{array}\right],$$

the goal is to get state-space realizations for four functions $N$, $M$, $X$, $Y$, all in $\mathcal{S}$, such that

$$G = \frac{N}{M}, \quad NX + MY = 1.$$

First, we look at how to get $N$ and $M$. If the input and output of $G$ are denoted $u$ and $y$, respectively, then the state model of $G$ is

$$\dot{x} = Ax + Bu, \tag{5.2}$$

$$y = Cx + Du. \tag{5.3}$$

Choose a real matrix $F$, $1 \times n$, such that $A + BF$ is stable (i.e., all eigenvalues in $\mathrm{Re}\, s < 0$). Now define the signal $v := u - Fx$. Then from (5.2) and (5.3) we get

$$\begin{aligned} \dot{x} &= (A + BF)x + Bv, \\ u &= Fx + v, \\ y &= (C + DF)x + Dv. \end{aligned}$$

Evidently from these equations, the transfer function from $v$ to $u$ is

$$M(s) := \left[\begin{array}{c|c} A+BF & B \\ \hline F & 1 \end{array}\right], \tag{5.4}$$

and that from $v$ to $y$ is

$$N(s) := \left[\begin{array}{c|c} A+BF & B \\ \hline C+DF & D \end{array}\right]. \tag{5.5}$$

Therefore,

$$u = Mv, \quad y = Nv,$$

so that $y = NM^{-1}u$, that is, $G = N/M$. Clearly, $N$ and $M$ are proper, and they are stable because $A+BF$ is. Thus $N, M \in \mathcal{S}$. Suggestion: Test the formulas above for the simplest case, $G(s) = 1/s$ ($A = 0$, $B = 1$, $C = 1$, $D = 0$).

The theory behind the formulas for $X$ and $Y$ is beyond the scope of this book. The procedure is to choose a real matrix $H$, $n \times 1$, so that $A + HC$ is stable, and then set

$$X(s) := \left[\begin{array}{c|c} A+HC & H \\ \hline F & 0 \end{array}\right], \tag{5.6}$$

$$Y(s) := \left[\begin{array}{c|c} A+HC & -B-HD \\ \hline F & 1 \end{array}\right]. \tag{5.7}$$

In summary, the procedure to do a coprime factorization of $G$ is this:

**Step 1** Get a realization $(A, B, C, D)$ of $G$.

**Step 2** Compute matrices $F$ and $H$ so that $A + BF$ and $A + HC$ are stable.

**Step 3** Using formulas (5.4) to (5.7), compute the four functions $N$, $M$, $X$, $Y$.

## 5.4 Controller Parametrization: General Plant

The transfer function $P$ is no longer assumed to be stable. Let $P = N/M$ be a coprime factorization over $\mathcal{S}$ and let $X$, $Y$ be two functions in $\mathcal{S}$ satisfying the equation

$$NX + MY = 1. \tag{5.8}$$

**Theorem 2** *The set of all $C$s for which the feedback system is internally stable equals*

$$\left\{ \frac{X + MQ}{Y - NQ} : Q \in \mathcal{S} \right\}.$$

It is useful to note that Theorem 2 reduces to Theorem 1 when $P \in \mathcal{S}$. To see this, recall from Section 5.2 (Step 1 of Procedure B) that we can take

$$N = P, \quad M = 1, \quad X = 0, \quad Y = 1$$

when $P \in \mathcal{S}$. Then

$$\frac{X + MQ}{Y - NQ} = \frac{Q}{1 - PQ}.$$

The proof of Theorem 2 requires a preliminary result.

**Lemma 1** *Let* $C = N_C/M_C$ *be a coprime factorization over* $\mathcal{S}$. *Then the feedback system is internally stable iff*

$$(NN_C + MM_C)^{-1} \in \mathcal{S}.$$

The proof of this lemma is almost identical to the proof of Theorem 3.1, and so is omitted.

**Proof of Theorem 2** ($\supset$) Suppose that $Q \in \mathcal{S}$ and

$$C := \frac{X + MQ}{Y - NQ}.$$

To show that the feedback system is internally stable, define

$$N_C := X + MQ, \quad M_C := Y - NQ.$$

Then from the equation

$$NX + MY = 1$$

it follows that

$$NN_C + MM_C = 1.$$

Therefore, $C = N_C/M_C$ is a coprime factorization, and from Lemma 1 the feedback system is internally stable.

($\subset$) Conversely, let $C$ be any controller achieving internal stability. We must find a $Q$ in $\mathcal{S}$ such that

$$C = \frac{X + MQ}{Y - NQ}.$$

Let $C = N_C/M_C$ be a coprime factorization over $\mathcal{S}$ and define

$$V := (NN_C + MM_C)^{-1}$$

so that

$$NN_CV + MM_CV = 1. \tag{5.9}$$

By Lemma 1, $V \in \mathcal{S}$. Let $Q$ be the solution of

$$M_CV = Y - NQ. \tag{5.10}$$

Substitute (5.10) into (5.9) to get

$$NN_CV + M(Y - NQ) = 1. \tag{5.11}$$

Also, add and subtract $NMQ$ in (5.8) to give

$$N(X + MQ) + M(Y - NQ) = 1. \tag{5.12}$$

Comparing (5.11) and (5.12), we see that

$$N_CV = X + MQ. \tag{5.13}$$

Now (5.10) and (5.13) give

$$C = \frac{N_CV}{M_CV} = \frac{X + MQ}{Y - NQ}.$$

It remains to show that $Q \in \mathcal{S}$. Multiply (5.10) by $X$ and (5.13) by $Y$, then subtract and switch sides:

$$(NX + MY)Q = YN_CV - XM_CV.$$

But the left-hand side equals $Q$ by (5.8), while the right-hand side belongs to $\mathcal{S}$. So we are done. ■

Theorem 2 gives an automatic way to stabilize a plant.

**Example** Let

$$P(s) = \frac{1}{(s-1)(s-2)}.$$

Apply Procedure B to get

$$\begin{aligned} N(s) &= \frac{1}{(s+1)^2}, \\ M(s) &= \frac{(s-1)(s-2)}{(s+1)^2}, \\ X(s) &= \frac{19s-11}{s+1}, \\ Y(s) &= \frac{s+6}{s+1}. \end{aligned}$$

According to the theorem, the controller

$$C(s) = \frac{X(s)}{Y(s)} = \frac{19s-11}{s+6}$$

achieves internal stability.

As before, when $P$ was stable, all closed-loop transfer functions are affine functions of $Q$ if $C$ is parametrized as in the theorem statement. For example, the sensitivity and complementary sensitivity functions are

$$\begin{aligned} S &= M(Y - NQ), \\ T &= N(X + MQ). \end{aligned}$$

Finally, it is sometimes useful to note that Lemma 1 suggests another way to solve the equation $NX + MY = 1$ given coprime $N$ and $M$. First, find a controller $C$ achieving internal stability for $P = N/M$—this might be easier than solving for $X$ and $Y$. Next, write a coprime factorization of $C$: $C = N_C/M_C$. Then Lemma 1 says that

$$V := NN_C + MM_C$$

is invertible in $\mathcal{S}$. Finally, set $X = N_CV^{-1}$ and $Y = M_CV^{-1}$.

## 5.5 Asymptotic Properties

How to find a $C$ to achieve internal stability and asymptotic properties simultaneously is perhaps best shown by an example.

Let

$$P(s) = \frac{1}{(s-1)(s-2)}.$$

The problem is to find a proper $C$ so that

1. The feedback system is internally stable.
2. The final value of $y$ equals 1 when $r$ is a unit step and $d = 0$.
3. The final value of $y$ equals zero when $d$ is a sinusoid of 10 rad/s and $r = 0$.

The first step is to parametrize all stabilizing $C$s. Suitable $N$, $M$, $X$, $Y$ are given in the example of the preceding section. From Theorem 2 $C$ must have the form

$$C = \frac{X + MQ}{Y - NQ} \tag{5.14}$$

for some $Q$ in $\mathcal{S}$ in order to satisfy (1). For such $C$ the transfer function from $r$ to $y$ equals $N(X + MQ)$. By the final-value theorem (2) holds iff

$$N(0)[X(0) + M(0)Q(0)] = 1. \tag{5.15}$$

Similarly, the transfer function from $d$ to $y$ equals $N(Y - NQ)$, so (3) holds iff

$$N(10j)[Y(10j) - N(10j)Q(10j)] = 0. \tag{5.16}$$

So the problem reduces to the purely algebraic one of finding a function $Q$ in $\mathcal{S}$ satisfying (5.15) and (5.16), which reduce to

$$\begin{aligned} Q(0) &= 6, \quad (5.17)\\ Q(10j) &= (6 + 10j)(1 + 10j) = -94 + 70j. \end{aligned}$$

This last equation is really two real equations:

$$\operatorname{Re} Q(10j) = -94, \tag{5.18}$$

$$\operatorname{Im} Q(10j) = 70. \tag{5.19}$$

So we must find a function $Q$ in $\mathcal{S}$ satisfying (5.17), (5.18), and (5.19).

A method that will certainly work is to let $Q$ be a polynomial in $(s+1)^{-1}$ with enough variable coefficients. This guarantees that $Q \in \mathcal{S}$. Since we need to satisfy three equations, we should allow three coefficients. So take $Q$ in the form

$$Q(s) = x_1 + x_2 \frac{1}{s+1} + x_3 \frac{1}{(s+1)^2}.$$

The three equations (5.17)-(5.19) lead to one of the form $Ax = b$, where

$$x = \begin{pmatrix} x_1 \\ x_2 \\ x_3 \end{pmatrix}.$$

Solve for $x$. In this case the solution is

$$x_1 = -79, \quad x_2 = -723, \quad x_3 = 808.$$

This gives

$$Q(s) = \frac{-79s^2 - 881s + 6}{(s+1)^2}.$$

Finally, we get $C$ from (5.14):

$$C(s) = \frac{-60s^4 - 598s^3 + 2515s^2 - 1794s + 1}{s(s^2 + 100)(s + 9)}.$$

In summary, the procedure consists of four steps:

1. Parametrize all internally stabilizing controllers.
2. Reduce the asymptotic specs to interpolation constraints on the parameter.
3. Find (if possible) a parameter to satisfy these constraints.
4. Back-substitute to get the controller.

## 5.6 Strong and Simultaneous Stabilization

Practicing control engineers are reluctant to use unstable controllers, especially if the plant itself is stable. System integrity is the motivation: For example, if a sensor or actuator fails, or is deliberately turned off during start-up or shutdown, and the feedback loop opens, overall stability is maintained if both plant and controller individually are stable. If the plant itself is unstable, the argument against using an unstable controller is less compelling. However, knowledge of when a plant is or is not stabilizable with a stable controller is useful for another problem, namely, simultaneous stabilization, meaning stabilization of several plants by the same controller.

The issue of simultaneous stabilization arises when a plant is subject to a discrete change, such as when a component burns out. Simultaneous stabilization of two plants can also be viewed as an example of a problem involving highly structured uncertainty. A set of plants with exactly two elements is the most extreme example of highly structured uncertainty, standing at the opposite end of the spectrum from the unstructured disk-like uncertainty, which is the type of uncertainty focused on in this book.

Say that a plant is *strongly stabilizable* if internal stabilization can be achieved with $C$ itself a stable transfer function. We start with an example of a plant that is not strongly stabilizable.

**Example 1** Consider the plant transfer function

$$P(s) = \frac{s-1}{s(s-2)}.$$

Every $C$ achieving internal stability is itself unstable. To prove this, start with a coprime factorization of $P$:

$$\begin{aligned} N(s) &= \frac{s-1}{(s+1)^2}, \\ M(s) &= \frac{s(s-2)}{(s+1)^2}, \\ X(s) &= \frac{14s-1}{s+1}, \\ Y(s) &= \frac{s-9}{s+1}. \end{aligned}$$

According to Theorem 2, all stabilizing controllers have the form

$$C = \frac{X+MQ}{Y-NQ}$$

for some $Q$ in $\mathcal{S}$. Since $X+MQ$ and $Y-NQ$ too are coprime—they satisfy the equation

$$N(X+MQ) + M(Y-NQ) = 1$$

—they have no common zero in Re$s \geq 0$. So to show that all such $C$s are unstable, it suffices to show that $Y - NQ$ has a zero in Re$s \geq 0$ for every $Q$ in $\mathcal{S}$. Now

$$N(1) = 0, \quad N(\infty) = 0,$$

so for every $Q$ in $\mathcal{S}$

$$\begin{aligned} (Y - NQ)(1) &= Y(1) \\ &= -4, \\ (Y - NQ)(\infty) &= Y(\infty) \\ &= 1. \end{aligned}$$

Notice that the two numbers on the right-hand side have opposite sign. Thus as $s$ moves along the positive real axis, the function $(Y - NQ)(s)$ changes sign. By continuity, it must equal zero at some such point, that is, $Y - NQ$ has a real zero somewhere on the positive real axis.

The poles and zeros of $P$ must share a certain property in order for $P$ to be strongly stabilizable. In the following theorem, the point at $s = \infty$ is included among the real zeros of $P$.

**Theorem 3** *$P$ is strongly stabilizable iff it has an even number of real poles between every pair of real zeros in Re$s \geq 0$.*

To illustrate, continue with the example above. The zeros, including the point at infinity, are at $s = 1, \infty$. Between this pair is a single pole, at $s = 2$. This plant therefore fails the test.

As another example, consider

$$P(s) = \frac{(s-1)^2(s^2 - s + 1)}{(s-2)^2(s+1)^3}.$$

On the positive real axis, including $\infty$, $P$ has three zeros, two at $s = 1$ and one at $s = \infty$. It has two other zeros in Re$s \geq 0$, which, not being real, are irrelevant. In counting poles between pairs of zeros we only have to consider distinct zeros (there are no poles between coincident zeros). Between zeros at $s = 1, \infty$ lie two poles, at $s = 2$. So this $P$ is strongly stabilizable.

**Proof of Theorem 3, Necessity** The proof is just as in Example 1. Assume that the pole-zero test fails. To show that every stabilizing controller is unstable, start with a coprime factorization of $P$,

$$P = \frac{N}{M}, \quad NX + MY = 1,$$

and some stabilizing controller,

$$C = \frac{X + MQ}{Y - NQ}, \quad Q \in \mathcal{S}.$$

It suffices to show that $Y - NQ$ has a zero in Re$s \geq 0$.

By assumption, there is some pair of real zeros of $N$ in Re$s \geq 0$, at $s = \sigma_1, \sigma_2$, say, with an odd number of zeros of $M$ in between. It follows that $M(\sigma_1)$ and $M(\sigma_2)$ have opposite sign; then so do $Y(\sigma_1)$ and $Y(\sigma_2)$, since $MY = 1$ at the right half-plane zeros of $N$. Hence the function $Y - NQ$ has a real zero somewhere between $s = \sigma_1$ and $s = \sigma_2$. ■

The proof of sufficiency is first illustrated by means of an example.

**Example 2** Take the plant transfer function

$$P(s) = \frac{s-1}{(s-2)^2}.$$

This has two poles, at $s = 2$, between the two zeros at $s = 1, \infty$, so $P$ is strongly stabilizable. To get a stable, stabilizing $C$, we should get a $Q$ in $\mathcal{S}$ such that the inverse of $U := Y - NQ$ belongs to $\mathcal{S}$. Equivalently, we should get a $U$ in $\mathcal{S}$ such that $U^{-1} \in \mathcal{S}$ and $U = Y$ at the two zeros of $N$, namely, $s = 1, \infty$. For this $P$ we have

$$N(s) = \frac{s-1}{(s+1)^2}, \quad M(s) = \frac{(s-2)^2}{(s+1)^2}.$$

Now

$$Y(1) = \frac{1}{M(1)} = 4, \quad Y(\infty) = \frac{1}{M(\infty)} = 1.$$

So the problem reduces to constructing a $U$ in $\mathcal{S}$ such that

$$U^{-1} \in \mathcal{S}, \quad U(1) = 4, \quad U(\infty) = 1.$$

The latter problem can be solved in two steps. First, get a $U_1$ in $\mathcal{S}$ such that

$$U_1^{-1} \in \mathcal{S}, \quad U_1(1) = 4.$$

The easiest choice is the constant $U_1(s) = 4$. Now we look for $U$ of the form

$$U = (1 + aF)^l U_1,$$

where $a$ is a constant, $l$ an integer, and $F \in \mathcal{S}$. To guarantee that $U(1) = U_1(1)$ we should arrange that $F(1) = 0$, for example,

$$F(s) = \frac{s-1}{s+1}.$$

Then for $U(\infty) = 1$ we need $(1 + a)^l 4 = 1$, that is,

$$a = \left(\frac{1}{4}\right)^{1/l} - 1, \tag{5.20}$$

and for $U^{-1} \in \mathcal{S}$ it suffices to have $\|aF\|_\infty < 1$ (i.e., $|a| < 1/\|F\|_\infty = 1$). So suitable $l$ and $a$ can be obtained by first choosing $l$ large enough that

$$\left| \left(\frac{1}{4}\right)^{1/l} - 1 \right| < 1$$

and then getting $a$ from (5.20), for example, $l = 1, a = -3/4$. This gives

$$U(s) = \left(1 - \frac{3}{4}\frac{s-1}{s+1}\right) 4 = \frac{s+7}{s+1}.$$

Finally, $U, M, N$ uniquely determine $C$, as follows:

$$U = Y - NQ \Longrightarrow Q = \frac{Y - U}{N} \Longrightarrow C = \frac{X + MQ}{Y - NQ} = \frac{1 - MU}{NU}.$$

For this example we get $C(s) = 27/(s+7)$. Notice that we did not actually have to construct $X$ and $Y$.

Now for the constructive procedure that proves sufficiency in Theorem 3. The general procedure is fairly involved, so a simplifying assumption will be made that $P$s unstable poles and zeros (including $\infty$) are all real and distinct. (Of course, Theorem 3 holds without this assumption.)

**Procedure**

**Step 0** Write $P = N/M$ with $N, M$ coprime. Arrange the non-negative real zeros of $N$ as follows:

$$0 \le \sigma_1 < \sigma_2 < \cdots < \sigma_m = \infty.$$

Define $r_i := 1/M(\sigma_i)$, $i = 1, \ldots, m$. Then $P$ is strongly stabilizable iff $r_1, \ldots, r_m$ all have the same sign. If this is true, continue.

**Step 1** Set $U_1(s) = r_1$.

Continue. Assume that $U_k$ has been constructed to satisfy

$$U_k, U_k^{-1} \in \mathcal{S}, \quad U_k(\sigma_i) = r_i, \quad i = 1, \ldots, k.$$

**Step** $k+1$ Choose $F$ in $\mathcal{S}$ to have zeros at $s = \sigma_1, \ldots, \sigma_k$. Choose $l \ge 1$ and $a$ so that

$$[1 + aF(\sigma_{k+1})]^l \, U_k(\sigma_{k+1}) = r_{k+1},$$

$$|a| < \frac{1}{\|F\|_\infty}.$$

Set $U_{k+1} = (1 + aF)^l U_k$.

Continue to Step $m$.

**Step** $m+1$ Set $U = U_m$ and $C = (1 - MU)/(NU)$.

Now we return to the problem of simultaneous stabilization and see that it can be reduced to one of strong stabilization. Two plants $P_1$ and $P_2$ are *simultaneously stabilizable* if internal stability is achievable for both by a common controller. Bring in coprime factorizations:

$$P_i = \frac{N_i}{M_i}, \quad N_i X_i + M_i Y_i = 1, \quad i = 1, 2$$

and define

$$N = N_2 M_1 - N_1 M_2, \quad M = N_2 X_1 + M_2 Y_1, \quad P = \frac{N}{M}.$$

For example, if $P_1$ is already stable, we may take

$$N_1 = P_1, \quad M_1 = 1, \quad X_1 = 0, \quad Y_1 = 1,$$

in which case

$$N = N_2 - P_1 M_2, \quad M = M_2,$$

so $P = P_2 - P_1$.

**Theorem 4** *$P_1$ and $P_2$ are simultaneously stabilizable iff $P$ is strongly stabilizable.*

**Proof** The controllers stabilizing $P_i$ are

$$\frac{X_i + M_i Q_i}{Y_i - N_i Q_i}, \quad Q_i \in \mathcal{S}.$$

Thus $P_1$ and $P_2$ are simultaneously stabilizable iff there exist $Q_1, Q_2$ in $\mathcal{S}$ such that

$$\frac{X_1 + M_1 Q_1}{Y_1 - N_1 Q_1} = \frac{X_2 + M_2 Q_2}{Y_2 - N_2 Q_2}.$$

Since these two fractions both have coprime factors, this equation holds iff there exists $U$ in $\mathcal{S}$ such that

$$\begin{aligned} U^{-1} &\in \mathcal{S}, \\ X_1 + M_1 Q_1 &= U(X_2 + M_2 Q_2), \\ Y_1 - N_1 Q_1 &= U(Y_2 - N_2 Q_2). \end{aligned}$$

To simplify the bookkeeping, write these last two equations in matrix form:

$$\begin{bmatrix} 1 & Q_1 \end{bmatrix} \begin{bmatrix} X_1 & Y_1 \\ M_1 & -N_1 \end{bmatrix} = U \begin{bmatrix} 1 & Q_2 \end{bmatrix} \begin{bmatrix} X_2 & Y_2 \\ M_2 & -N_2 \end{bmatrix}.$$

Postmultiply this equation by

$$\begin{bmatrix} N_2 & Y_2 \\ M_2 & -X_2 \end{bmatrix}$$

to get

$$\begin{bmatrix} 1 & Q_1 \end{bmatrix} \begin{bmatrix} X_1 & Y_1 \\ M_1 & -N_1 \end{bmatrix} \begin{bmatrix} N_2 & Y_2 \\ M_2 & -X_2 \end{bmatrix} = U \begin{bmatrix} 1 & Q_2 \end{bmatrix}.$$

Now the first column of the matrix

$$\begin{bmatrix} X_1 & Y_1 \\ M_1 & -N_1 \end{bmatrix} \begin{bmatrix} N_2 & Y_2 \\ M_2 & -X_2 \end{bmatrix}$$

is

$$\begin{bmatrix} M \\ N \end{bmatrix};$$

denote the second column by

$$\begin{bmatrix} X \\ Y \end{bmatrix}.$$

Then the previous matrix equation is equivalent to the two equations

$$\begin{aligned} M + NQ_1 &= U, \\ X + YQ_1 &= UQ_2. \end{aligned}$$

To recap, $P_1$ and $P_2$ are simultaneously stabilizable iff there exist $Q_1, Q_2, U$ in $\mathcal{S}$ such that

$$\begin{aligned} U^{-1} &\in \mathcal{S}, \\ M + NQ_1 &= U, \\ X + YQ_1 &= UQ_2. \end{aligned}$$

Clearly, this is equivalent to the condition, there exist $Q_1, U$ in $\mathcal{S}$ such that

$$\begin{aligned} U^{-1} &\in \mathcal{S}, \\ M + NQ_1 &= U \end{aligned}$$

[because we can get $Q_2$ via $(X + YQ_1)/U$]. But it can be checked that $N$ and $M$ are coprime. Thus from Lemma 1 the previous condition is equivalent to, $P$ can be stabilized by some stable controller, namely, $Q_1$. ■

**Example 3** Consider

$$P_1(s) = \frac{1}{s+1}, \quad P_2(s) = \frac{as+b}{(s+1)(s-1)},$$

where $a$ and $b$ are real constants with $a \neq 1$. Since $P_1$ is stable, we have

$$P(s) = P_2(s) - P_1(s) = -\frac{(1-a)s - (1+b)}{(s+1)(s-1)}.$$

This has zeros at

$$s = \frac{1+b}{1-a}, \infty$$

and a simple unstable pole at $s = 1$. So $P$ is strongly stabilizable, or $P_1$ and $P_2$ are simultaneously stabilizable, iff either the zero $(1+b)/(1-a)$ is negative or it lies to the right of the unstable pole, that is,

$$\text{either } \frac{1+b}{1-a} < 0 \text{ or } \frac{1+b}{1-a} > 1.$$

Simultaneous stabilization for more than two plants is still an unsolved problem.

## 5.7 Cart-Pendulum Example

An interesting stabilization problem is afforded by the cart-pendulum example, a common toy control system. The setup is shown in Figure 5.2. The system consists of a cart of mass $M$ that slides in one dimension $x$ on a horizontal surface, with a ball of mass $m$ at the end of a rigid massless pendulum of length $l$. The cart and ball are treated as point masses, with the pivot at the center of the cart. There is assumed to be no friction and no air resistance. Shown as inputs are a horizontal force $u$ on the cart and a force $d$ on the ball perpendicular to the pendulum. The other signals shown are the angle $\theta$ and the position of the ball $y = x + l\sin\theta$.

Elementary dynamics yields the following equations of motion:

$$(M+m)\ddot{x} + ml(\ddot{\theta}\cos\theta - \dot{\theta}^2\sin\theta) = u + d\cos\theta, \quad (5.21)$$

$$m\left(\ddot{x}\cos\theta + l\ddot{\theta} - g\sin\theta\right) = d. \quad (5.22)$$

These are nonlinear equations which can be linearized about an equilibrium position, of which there are two: $(x,\theta) = (0,0)$ and $(x,\theta) = (0,\pi)$ (i.e., the pendulum either up or down).

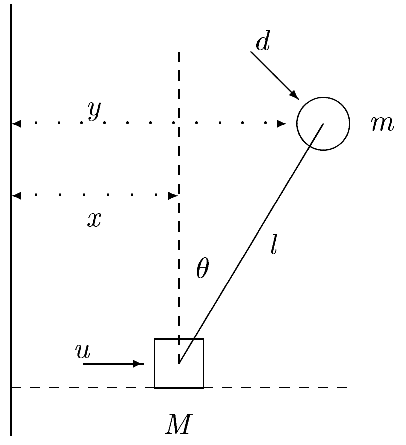


Figure 5.2: Cart-pendulum example.

### Linearization About Pendulum Up

The two linearized equations are

$$\begin{aligned}(M+m)\ddot{x}+ml\ddot{\theta} &= u+d,\\ \ddot{x}+l\ddot{\theta}-g\theta &= \frac{1}{m}d.\end{aligned}$$

Take Laplace transforms to get

$$\begin{bmatrix}(M+m)s^2 & mls^2\\ s^2 & ls^2-g\end{bmatrix}\begin{bmatrix}\hat{x}\\ \hat{\theta}\end{bmatrix}=\begin{bmatrix}\hat{u}+\hat{d}\\ \frac{1}{m}\hat{d}\end{bmatrix}.$$

Thus

$$\begin{bmatrix}\hat{x}\\ \hat{\theta}\end{bmatrix}=\frac{1}{D(s)}\begin{bmatrix}ls^2-g & -g\\ -s^2 & \frac{M}{m}s^2\end{bmatrix}\begin{bmatrix}\hat{u}\\ \hat{d}\end{bmatrix},$$

where

$$D(s)=s^2[Mls^2-(M+m)g].$$

Finally,

$$\hat{y}=\hat{x}+l\hat{\theta}=\frac{1}{D(s)}\begin{bmatrix}-g & \frac{M}{m}ls^2g\end{bmatrix}\begin{bmatrix}\hat{u}\\ \hat{d}\end{bmatrix}.$$

In particular, the transfer functions from $u$ to $x$ and $y$ are, respectively,

$$\frac{ls^2-g}{D(s)},\quad \frac{-g}{D(s)}.$$

These are both unstable, having right half-plane poles at

$$s=0,\ 0,\ \sqrt{\frac{(M+m)g}{Ml}}.$$

Also, the transfer function from $u$ to $x$ has a right half-plane zero at $s=\sqrt{g/l}$.

### Linearization About Pendulum Down

Replacing $\theta$ by $\pi + \theta$ in equations (5.21) and (5.22) and linearizing, we get

$$\begin{aligned}(M+m)\ddot{x} - ml\ddot{\theta} &= u - d,\\ -\ddot{x} + l\ddot{\theta} + g\theta &= \frac{1}{m}d,\end{aligned}$$

so

$$\begin{bmatrix}\hat{x}\\ \hat{\theta}\end{bmatrix} = \frac{1}{D(s)}\begin{bmatrix} ls^2+g & -g\\ s^2 & \frac{M}{m}s^2\end{bmatrix}\begin{bmatrix}\hat{u}\\ \hat{d}\end{bmatrix},$$

and

$$\hat{y} = \hat{x} - l\hat{\theta} = \frac{1}{D(s)}\begin{bmatrix} g & -\frac{M}{m}ls^2g\end{bmatrix}\begin{bmatrix}\hat{u}\\ \hat{d}\end{bmatrix},$$

where

$$D(s) = s^2[Mls^2 + (M+m)g].$$

The transfer functions from $u$ to $x$ and $y$ are now, respectively,

$$\frac{ls^2+g}{D(s)}, \quad \frac{g}{D(s)}.$$

Let us look at the problem of stabilizing the $u$-to-$x$ transfer function with the pendulum in the up position. The transfer function is

$$\frac{ls^2-g}{s^2[Mls^2-(M+m)g]}.$$

Since this has an unstable pole, namely,

$$\sqrt{\frac{(M+m)g}{Ml}},$$

between two real zeros at $s = \sqrt{g/l}, \infty$, from the preceding section this transfer function is not strongly stabilizable. Having no finite zeros, the $u$-to-$y$ transfer function is, however.

Is the cart-pendulum stabilizable if we measure $x$ and control $d$? First of all, what does this mean? The cart-pendulum as configured is really a multivariable system: It has two inputs, $u$ and $d$, and two outputs, $x$ and $\theta$ ($y$ is a linear combination of these two). So really there are four loops we could close: from $x$ to $u$ and $d$ and from $\theta$ to $u$ and $d$. Let us contemplate closing just from $x$ to $d$. We would like all closed-loop transfer functions to be stable, for example, $u$-to-$\theta$, $x$-to-$\theta$, and so on. Is this possible?

(This analysis applies only to the linearized system. Since there are poles on the imaginary axis, the stability of the linear system does not determine even the local stability of the nonlinear system.)

We shall return to this example in the next chapter.

## Exercises

1. Compute a coprime factorization over $\mathcal{S}$ of
$$G(s) = \frac{s^3}{s^2 - s + 1}.$$

2. For
$$P(s) = \frac{3}{s-4}$$
compute a controller $C$ so that the feedback system is internally stable and the tracking error $e$ goes to 0 when $r$ is a ramp and $d = 0$.

3. For
$$P(s) = \frac{1}{s(s^2 + 0.2s + 1)}$$
find an internally stabilizing $C$ so that the final value of $r - y$ equals zero when $r$ is a unit ramp and $d$ is a sinusoid of frequency 2 rad/s.

4. Suppose that $P(s) = 1/s$ and $C = Q/(1 - PQ)$, where $Q$ is a proper real-rational function. Characterize those functions $Q$ for which the feedback system is internally stable.

5. Suppose that $N, M$ are coprime functions in $\mathcal{S}$. Prove that if $NM^{-1} \in \mathcal{S}$, then $M^{-1} \in \mathcal{S}$. Is this true without the coprimeness assumption?

6. The problem is to find an internally stabilizing $C$ so that $e$ tends to zero asymptotically when $r$ is a step and $d = 0$. When is the problem solvable? Characterize all solutions. (Do not assume $P$ is stable.)

7. Let
$$P(s) = \frac{s}{(s-1)(10s+1)}.$$
Find a $C$ to achieve internal stability. What are the closed-loop poles? What is the dc gain from $d$ to $y$?

8. For formulas (5.4) to (5.7), verify that $NX + MY = 1$.

9. Consider the feedback system with plant $P$ and controller $C$. Assume internal stability. Consider a coprime factorization of $P$ over $\mathcal{S}$, $P = N/M$. Suppose that $P$ is perturbed to
$$P = \frac{N + \Delta_1}{M + \Delta_2},$$
where
$$\begin{aligned} \Delta_1, \Delta_2 &\in \mathcal{S}, \\ \|\Delta_1\|_\infty, \|\Delta_2\|_\infty &\leq \gamma. \end{aligned}$$
Find a bound on $\gamma$ for robust stability.

10. Compute a stable, stabilizing controller for
$$P(s) = \frac{(s-1)(s^2+s+1)}{(s-2)(s-3)(s+1)^2}.$$

11. Study simultaneous stabilization of the cart-pendulum system in the up and down configurations.

## Notes and References

The idea behind Theorem 1 is due to Newton et al. (1957). They observed that while the transfer function from $r$ to $y$ is nonlinear in $C$, it is linear in $Q$, the transfer function from $r$ to $u$. So they proposed to design $Q$ to achieve desired performance and then obtain $C$ by back-substitution. Theorem 1 itself is due to Zames (1981). The controller parametrization in Theorem 1 is used in the field of process control, where it is called *internal model control* [because the controller $C = Q/(1 - PQ)$ contains a model of the plant $P$] (Morari and Zafiriou, 1989).

The original form of Theorem 2 is due independently to Youla et al. (1976) and Kucera (1979). Its present form is due to Desoer et al. (1980), who saw the advantage of doing coprime factorization over $\mathcal{S}$ instead of the ring of polynomials. This idea in turn is due to Vidyasagar (1972). State-space formulas for coprime factorization were first derived by Khargonekar and Sontag (1982). The formulas in Section 5.3 are from Nett et al. 1984. Section 5.5 is adapted from Francis and Vidyasagar (1983). The algebraic point of view has been explored in detail by Desoer and co-workers (e.g., Desoer and Gustafson, 1984) and by Vidyasagar (1985). The notion of strong stabilization and Theorem 3 are due to Youla et al. (1974). Simultaneous stabilization was first treated by Saeks and Murray (1982). The simple proofs of Theorems 3 and 4 given here are borrowed from Vidyasagar (1985). The controller parametrization of Theorem 2 has been exploited in a CAD method for controller design (Boyd et al., 1988).

# Chapter 6

# Design Constraints

Before we see how to design control systems for the robust performance specification, it is useful to determine the basic limitations on achievable performance. In this chapter we study design constraints arising from two sources: from algebraic relationships that must hold among various transfer functions; from the fact that closed-loop transfer functions must be stable (i.e., analytic in the right half-plane). It is assumed throughout this chapter that the feedback system is internally stable.

## 6.1 Algebraic Constraints

There are three items in this section.

1. The identity $S + T = 1$ always holds. This is an immediate consequence of the definitions of $S$ and $T$. So in particular, $|S(j\omega)|$ and $|T(j\omega)|$ cannot both be less than 1/2 at the same frequency $\omega$.

2. A necessary condition for robust performance is that the weighting functions satisfy

$$\min\{|W_1(j\omega)|, |W_2(j\omega)|\} < 1, \quad \forall\omega.$$

**Proof** Fix $\omega$ and assume that $|W_1| \leq |W_2|$ (the argument $j\omega$ is suppressed). Then

$$\begin{aligned} |W_1| &= |W_1(S+T)| \\ &\leq |W_1 S| + |W_1 T| \\ &\leq |W_1 S| + |W_2 T|. \end{aligned}$$

So robust performance (see Theorem 4.2), that is,

$$\| |W_1 S| + |W_2 T| \|_\infty < 1,$$

implies that $|W_1| < 1$, and hence

$$\min\{|W_1|, |W_2|\} < 1.$$

The same conclusion can be drawn when $|W_2| \leq |W_1|$. ■

So at every frequency either $|W_1|$ or $|W_2|$ must be less than 1. Typically, $|W_1(j\omega)|$ is monotonically decreasing—for good tracking of low-frequency signals—and $|W_2(j\omega)|$ is monotonically increasing—uncertainty increases with increasing frequency.

3. If $p$ is a pole of $L$ in $\text{Re}s \geq 0$ and $z$ is a zero of $L$ in the same half-plane, then

$$S(p) = 0, \quad S(z) = 1, \tag{6.1}$$

$$T(p) = 1, \quad T(z) = 0. \tag{6.2}$$

These interpolation constraints are immediate from the definitions of $S$ and $T$. For example,

$$S(p) = \frac{1}{1 + L(p)} = \frac{1}{\infty} = 0.$$

## 6.2 Analytic Constraints

In this section we derive some constraints concerning achievable performance obtained from analytic function theory. The first subsection presents some mathematical preliminaries.

### Preliminaries

We begin with the following fundamental facts concerning complex functions: the maximum modulus theorem, Cauchy's theorem, and Cauchy's integral formula. These are stated here for convenience.

**Maximum Modulus Theorem** *Suppose that $\Omega$ is a region (nonempty, open, connected set) in the complex plane and $F$ is a function that is analytic in $\Omega$. Suppose that $F$ is not equal to a constant. Then $|F|$ does not attain its maximum value at an interior point of $\Omega$.*

A simple application of this theorem, with $\Omega$ equal to the open right half-plane, shows that for $F$ in $\mathcal{S}$

$$\|F\|_\infty = \sup_{\text{Re}s > 0} |F(s)|.$$

**Cauchy's Theorem** *Suppose that $\Omega$ is a bounded open set with connected complement and $\mathcal{D}$ is a non-self-intersecting closed contour in $\Omega$. If $F$ is analytic in $\Omega$, then*

$$\oint_{\mathcal{D}} F(s)ds = 0.$$

**Cauchy's Integral Formula** *Suppose that $F$ is analytic on a non-self-intersecting closed contour $\mathcal{D}$ and in its interior $\Omega$. Let $s_0$ be a point in $\Omega$. Then*

$$F(s_0) = \frac{1}{2\pi j} \oint_{\mathcal{D}} \frac{F(s)}{s - s_0} ds.$$

We shall also need the Poisson integral formula, which says that the value of a bounded analytic function at a point in the right half-plane is completely determined by the coordinates of the point together with the values of the function on the imaginary axis.

**Lemma 1** *Let $F$ be analytic and of bounded magnitude in $\text{Re}s \geq 0$ and let $s_0 = \sigma_0 + j\omega_0$ be a point in the complex plane with $\sigma_0 > 0$. Then*

$$F(s_0) = \frac{1}{\pi} \int_{-\infty}^{\infty} F(j\omega) \frac{\sigma_0}{\sigma_0^2 + (\omega - \omega_0)^2} d\omega.$$

**Proof** Construct the Nyquist contour $\mathcal{D}$ in the complex plane taking the radius, $r$, large enough so that the point $s_0$ is encircled by $\mathcal{D}$.

Cauchy's integral formula gives

$$F(s_0) = \frac{1}{2\pi j} \oint_{\mathcal{D}} \frac{F(s)}{s - s_0} ds.$$

Also, since $-\overline{s}_0$ is not encircled by $\mathcal{D}$, Cauchy's theorem gives

$$0 = \frac{1}{2\pi j} \oint_{\mathcal{D}} \frac{F(s)}{s + \overline{s}_0} ds.$$

Subtract these two equations to get

$$F(s_0) = \frac{1}{2\pi j} \oint_{\mathcal{D}} F(s) \frac{\overline{s}_0 + s_0}{(s - s_0)(s + \overline{s}_0)} ds.$$

Thus

$$F(s_0) = I_1 + I_2,$$

where

$$\begin{aligned}
I_1 &:= \frac{1}{\pi} \int_{-r}^{r} F(j\omega) \frac{\sigma_0}{(s_0 - j\omega)(\overline{s}_0 + j\omega)} d\omega \\
&= \frac{1}{\pi} \int_{-r}^{r} F(j\omega) \frac{\sigma_0}{\sigma_0^2 + (\omega - \omega_0)^2} d\omega, \\
I_2 &:= \frac{1}{\pi j} \int_{-\pi/2}^{\pi/2} F(re^{j\theta}) \frac{\sigma_0}{(re^{j\theta} - s_0)(re^{j\theta} + \overline{s}_0)} rje^{j\theta} d\theta.
\end{aligned}$$

As $r \to \infty$

$$I_1 \to \frac{1}{\pi} \int_{-\infty}^{\infty} F(j\omega) \frac{\sigma_0}{\sigma_0^2 + (\omega - \omega_0)^2} d\omega.$$

So it remains to show that $I_2 \to 0$ as $r \to \infty$.

We have

$$I_2 \leq \frac{\sigma_0}{\pi} \|F\|_\infty \frac{1}{r} \int_{-\pi/2}^{\pi/2} \frac{1}{|e^{j\theta} - s_0 r^{-1}||e^{j\theta} + \overline{s}_0 r^{-1}|} d\theta.$$

The integral

$$\int_{-\pi/2}^{\pi/2} \frac{1}{|e^{j\theta} - s_0 r^{-1}||e^{j\theta} + \overline{s}_0 r^{-1}|} d\theta$$

converges as $r \to \infty$. Thus

$$I_2 \leq \text{constant} \times \frac{1}{r},$$

which gives the desired result. ■

### Bounds on the Weights $W_1$ and $W_2$

Suppose that the loop transfer function $L$ has a zero $z$ in $\mathrm{Re}s \geq 0$. Then

$$\|W_1 S\|_\infty \geq |W_1(z)|. \tag{6.3}$$

This is a direct consequence of the maximum modulus theorem and (6.1):

$$|W_1(z)| = |W_1(z)S(z)| \leq \sup_{\mathrm{Re}s \geq 0} |W_1(s)S(s)| = \|W_1 S\|_\infty.$$

So a necessary condition that the performance criterion $\|W_1 S\|_\infty < 1$ be achievable is that the weight satisfy $|W_1(z)| < 1$. In words, the magnitude of the weight at a right half-plane zero of $P$ or $C$ must be less than 1.

Similarly, suppose that $L$ has a pole $p$ in $\mathrm{Re}s \geq 0$. Then

$$\|W_2 T\|_\infty \geq |W_2(p)|, \tag{6.4}$$

so a necessary condition for the robust stability criterion $\|W_2 T\|_\infty < 1$ is that the weight $W_2$ satisfy $|W_2(p)| < 1$.

### All-Pass and Minimum-Phase Transfer Functions

Two types of transfer functions play a critical role in the rest of this book: all-pass and minimum-phase. A function in $\mathcal{S}$ is *all-pass* if its magnitude equals 1 at all points on the imaginary axis. The terminology comes from the fact that a filter with an all-pass transfer function passes without attenuation input sinusoids of all frequencies. It is not difficult to show that such a function has pole-zero symmetry about the imaginary axis in the sense that a point $s_0$ is a zero iff its reflection, $-\overline{s}_0$, is a pole. Consequently, the function being stable, all its zeros lie in the right half-plane. Thus an all-pass function is, up to sign, the product of factors of the form

$$\frac{s - s_0}{s + \overline{s}_0}, \qquad \mathrm{Re}s_0 > 0.$$

Examples of all-pass functions are

$$1, \quad \frac{s-1}{s+1}, \quad \frac{s^2 - s + 2}{s^2 + s + 2}.$$

A function in $\mathcal{S}$ is *minimum-phase* if it has no zeros in $\mathrm{Re}s > 0$. This terminology can be explained as follows. Let $G$ be a minimum-phase transfer function. There are many other transfer functions having the same magnitude as $G$, for example $FG$ where $F$ is all-pass. But all these other transfer functions have greater phase. Thus, of all the transfer functions having $G$s magnitude, the one with the minimum phase is $G$. Examples of minimum-phase functions are

$$1, \quad \frac{1}{s+1}, \quad \frac{s}{s+1}, \quad \frac{s+2}{s^2+s+1}.$$

It is a useful fact that every function in $\mathcal{S}$ can be written as the product of two such factors: for example

$$\frac{4(s-2)}{s^2+s+1} = \left(\frac{s-2}{s+2}\right)\left(\frac{4(s+2)}{s^2+s+1}\right).$$

**Lemma 2** *For each function $G$ in $\mathcal{S}$ there exist an all-pass function $G_{ap}$ and a minimum-phase function $G_{mp}$ such that $G = G_{ap}G_{mp}$. The factors are unique up to sign.*

**Proof** Let $G_{ap}$ be the product of all factors of the form

$$\frac{s - s_0}{s + \overline{s}_0},$$

where $s_0$ ranges over all zeros of $G$ in $\mathrm{Re}s > 0$, counting multiplicities, and then define

$$G_{mp} = \frac{G}{G_{ap}}.$$

The proof of uniqueness is left as an exercise. ■

For technical reasons we assume for the remainder of this section that $L$ has no poles on the imaginary axis. Factor the sensitivity function as

$$S = S_{ap}S_{mp}.$$

Then $S_{mp}$ has no zeros on the imaginary axis (such zeros would be poles of $L$) and $S_{mp}$ is not strictly proper (since $S$ is not). Thus $S_{mp}^{-1} \in \mathcal{S}$.

As a simple example of the use of all-pass functions, suppose that $P$ has a zero at $z$ with $z > 0$, a pole at $p$ with $p > 0$; also, suppose that $C$ has neither poles nor zeros in the closed right half-plane. Then

$$S_{ap}(s) = \frac{s-p}{s+p}, \quad T_{ap}(s) = \frac{s-z}{s+z}.$$

It follows from the preceding section that $S(z) = 1$, and hence

$$S_{mp}(z) = S_{ap}(z)^{-1} = \frac{z+p}{z-p}.$$

Similarly,

$$T_{mp}(p) = T_{ap}(p)^{-1} = \frac{p+z}{p-z}.$$

Then

$$\|W_1 S\|_\infty = \|W_1 S_{mp}\|_\infty \geq |W_1(z) S_{mp}(z)| = \left| W_1(z) \frac{z+p}{z-p} \right|$$

and

$$\|W_2 T\|_\infty \geq \left| W_2(p) \frac{p+z}{p-z} \right|.$$

Thus, if there are a pole and zero close to each other in the right half-plane, they can greatly amplify the effect that either would have alone.

**Example** These inequalities are effectively illustrated with the cart-pendulum example of Section 5.7. Let $P(s)$ be the $u$-to-$x$ transfer function for the up position of the pendulum, that is,

$$P(s) = \frac{ls^2 - g}{s^2[Mls^2 - (M+m)g]}.$$

Define the ratio $r := m/M$ of pendulum mass to cart mass. The zero and pole of $P$ in $\text{Re}s > 0$ are

$$z = \sqrt{\frac{g}{l}}, \quad p = z\sqrt{1+r}.$$

Note that for $r$ fixed, a larger value of $l$ means a smaller value of $p$, and this in turn means that the system is easier to stabilize (the time constant is slower). The foregoing two inequalities on $\|W_1S\|_\infty$ and $\|W_2T\|_\infty$ apply. Since the cart-pendulum is a stabilization task, let us focus on

$$\|W_2T\|_\infty \geq \left| W_2(p)\frac{p+z}{p-z} \right|. \tag{6.5}$$

The robust stabilization problem becomes harder the larger the value of the right-hand side of (6.5). The scaling factor in this inequality is

$$\frac{p+z}{p-z} = \frac{\sqrt{1+r}+1}{\sqrt{1+r}-1}. \tag{6.6}$$

This quantity is always greater than 1, and it approaches 1 only when $r$ approaches $\infty$, that is, only when the pendulum mass is much larger than the cart mass. There is a tradeoff, however, in that a large value of $r$ means a large value of $p$, the unstable pole; for a typical $W_2$ (high-pass) this in turn means a relatively large value of $|W_2(p)|$ in (6.5). So at least for small uncertainty, the worst-case scenario is a short pendulum with a small mass $m$ relative to the cart mass $M$.

In contrast, the $u$-to-$y$ transfer function has no zeros, so the constraint there is simply

$$\|W_2T\|_\infty \geq |W_2(p)|.$$

If robust stabilization were the only objective, we could achieve equality by careful selection of the controller. Note that for this case there is no apparent tradeoff in making $m/M$ large. The difference between the two cases, measuring $x$ and measuring $y$, again highlights the important fact that sensor location can have a significant effect on the difficulty of controlling a system or on the ultimate achievable performance.

Some simple experiments can be done to illustrate the points made in this example. Obtain several sticks of various lengths and try to balance them in the palm of your hand. You will notice that it is easier to balance longer sticks, because the dynamics are slower and $p$ above is smaller. It is also easier to balance the sticks if you look at the top of the stick (measuring $y$) rather than at the bottom (measuring $x$). In fact, even for a stick that is easily balanced when looking at the top, it will be impossible to balance it while looking only at the bottom. There is also feedback from the forces that your hand feels, but this is similar to measuring $x$.

The interested reader may repeat the analysis for the down position of the pendulum. At this point it is useful to include the following lemma which will be used subsequently.

**Lemma 3** *For every point $s_0 = \sigma_0 + j\omega_0$ with $\sigma_0 > 0$,*

$$\log|S_{mp}(s_0)| = \frac{1}{\pi}\int_{-\infty}^{\infty} \log|S(j\omega)| \frac{\sigma_0}{\sigma_0^2 + (\omega-\omega_0)^2} d\omega.$$

**Proof** Set $F(s) := \ln S_{mp}(s)$. Then $F$ is analytic and of bounded magnitude in $\mathrm{Re}s \geq 0$. (This follows from the properties $S_{mp}, S_{mp}^{-1} \in \mathcal{S}$; the idea is that since $S_{mp}$ has no poles or zeros in the right half-plane, $\ln S_{mp}$ is well-behaved there.) Apply Lemma 1 to get

$$F(s_0) = \frac{1}{\pi}\int_{-\infty}^{\infty} F(j\omega)\frac{\sigma_0}{\sigma_0^2 + (\omega - \omega_0)^2}d\omega.$$

Now take real parts of both sides:

$$\mathrm{Re}F(s_0) = \frac{1}{\pi}\int_{-\infty}^{\infty} \mathrm{Re}F(j\omega)\frac{\sigma_0}{\sigma_0^2 + (\omega - \omega_0)^2}d\omega. \qquad (6.7)$$

But

$$S_{mp} = \mathrm{e}^F = \mathrm{e}^{\mathrm{Re}F}\mathrm{e}^{j\mathrm{Im}F},$$

so

$$|S_{mp}| = \mathrm{e}^{\mathrm{Re}F},$$

that is,

$$\ln|S_{mp}| = \mathrm{Re}F.$$

Thus from (6.7)

$$\ln|S_{mp}(s_0)| = \frac{1}{\pi}\int_{-\infty}^{\infty} \ln|S_{mp}(j\omega)|\frac{\sigma_0}{\sigma_0^2 + (\omega - \omega_0)^2}d\omega,$$

or since $|S| = |S_{mp}|$ on the imaginary axis,

$$\ln|S_{mp}(s_0)| = \frac{1}{\pi}\int_{-\infty}^{\infty} \ln|S(j\omega)|\frac{\sigma_0}{\sigma_0^2 + (\omega - \omega_0)^2}d\omega.$$

Finally, since $\log x = \log \mathrm{e} \ln x$, the result follows upon multiplying the last equation by $\log \mathrm{e}$. ■

## The Waterbed Effect

Consider a tracking problem where the reference signals have their energy spectra concentrated in a known frequency range, say $[\omega_1, \omega_2]$. This is the idealized situation where $W_1$ is a bandpass filter. Let $M_1$ denote the maximum magnitude of $S$ on this frequency band,

$$M_1 := \max_{\omega_1 \leq \omega \leq \omega_2} |S(j\omega)|,$$

and let $M_2$ denote the maximum magnitude over all frequencies, that is, $\|S\|_\infty$. Then good tracking capability is characterized by the inequality $M_1 \ll 1$. On the other hand, we cannot permit $M_2$ to be too large: Remember (Section 4.2) that $1/M_2$ equals the distance from the critical point to the Nyquist plot of $L$, so large $M_2$ means small stability margin (a typical upper bound for $M_2$ is 2). Notice that $M_2$ must be at least 1 because this is the value of $S$ at infinite frequency. So the question arises: Can we have $M_1$ very small and $M_2$ not too large? Or does it happen that very small $M_1$ necessarily means very large $M_2$? The latter situation might be compared to a waterbed: As $|S|$ is pushed down on one frequency range, it pops up somewhere else. It turns out that non-minimum-phase plants exhibit the waterbed effect.

**Theorem 1** *Suppose that $P$ has a zero at $z$ with $Rez > 0$. Then there exist positive constants $c_1$ and $c_2$, depending only on $\omega_1$, $\omega_2$, and $z$, such that*

$$c_1 \log M_1 + c_2 \log M_2 \geq \log|S_{ap}(z)^{-1}| \geq 0.$$

**Proof** Since $z$ is a zero of $P$, it follows from the preceding section that $S(z) = 1$, and hence $S_{mp}(z) = S_{ap}(z)^{-1}$. Apply Lemma 3 with

$$s_0 = z = \sigma_0 + j\omega_0$$

to get

$$\log |S_{ap}(z)^{-1}| = \frac{1}{\pi} \int_{-\infty}^{\infty} \log |S(j\omega)| \frac{\sigma_0}{\sigma_0^2 + (\omega - \omega_0)^2} d\omega.$$

Thus

$$\log |S_{ap}(z)^{-1}| \leq c_1 \log M_1 + c_2 \log M_2,$$

where $c_1$ is defined to be the integral of

$$\frac{1}{\pi} \frac{\sigma_0}{\sigma_0^2 + (\omega - \omega_0)^2}$$

over the set

$$[-\omega_2, -\omega_1] \cup [\omega_1, \omega_2]$$

and $c_2$ equals the same integral but over the complementary set.

It remains to observe that $|S_{ap}(z)| \leq 1$ by the maximum modulus theorem, so

$$\log |S_{ap}(z)^{-1}| \geq 0. \blacksquare$$

**Example** As an illustration of the theorem consider the plant transfer function

$$P(s) = \frac{s-1}{(s+1)(s-p)},$$

where $p > 0$, $p \neq 1$. As observed in the preceding section, $S$ must interpolate zero at the unstable poles of $P$, so $S(p) = 0$. Thus the all-pass factor of $S$ must contain the factor

$$\frac{s-p}{s+p},$$

that is,

$$S_{ap}(s) = \frac{s-p}{s+p} G(s)$$

for some all-pass function $G$. Since $|G(1)| \leq 1$ (maximum modulus theorem), there follows

$$|S_{ap}(1)| \leq \left| \frac{1-p}{1+p} \right|.$$

So the theorem gives

$$c_1 \log M_1 + c_2 \log M_2 \geq \log \left| \frac{1+p}{1-p} \right|.$$

Note that the right-hand side is very large if $p$ is close to 1. This example illustrates again a general fact: The waterbed effect is amplified if the plant has a pole and a zero close together in the right half-plane. We would expect such a plant to be very difficult to control.

It is emphasized that the waterbed effect applies to non-minimum-phase plants only. In fact, the following can be proved (Section 10.1): If $P$ has no zeros in $\text{Re}s > 0$ nor on the imaginary axis

in the frequency range $[\omega_1, \omega_2]$, then for every $\epsilon > 0$ and $\delta > 1$ there exists a controller $C$ so that the feedback system is internally stable, $M_1 < \epsilon$, and $M_2 < \delta$. As a very easy example, take

$$P(s) = \frac{1}{s+1}.$$

The controller $C(s) = k$ is internally stabilizing for all $k > 0$, and then

$$S(s) = \frac{s+1}{s+1+k}.$$

So $\|S\|_\infty = 1$ and, for every $\epsilon > 0$ and $\omega_2$, if $k$ is large enough, then

$$|S(j\omega)| < \epsilon, \quad \forall \omega \le \omega_2.$$

## The Area Formula

Herein is derived a formula for the area bounded by the graph of $|S(j\omega)|$ (log scale) plotted as a function of $\omega$ (linear scale). The formula is valid when the relative degree of $L$ is large enough. *Relative degree* equals degree of denominator minus degree of numerator.

Let $\{p_i\}$ denote the set of poles of $L$ in $\mathrm{Re}\, s > 0$.

**Theorem 2** *Assume that the relative degree of $L$ is at least 2. Then*

$$\int_0^\infty \log |S(j\omega)| d\omega = \pi (\log \mathrm{e}) (\sum \mathrm{Re} p_i).$$

**Proof** In Lemma 3 take $\omega_0 = 0$ to get

$$\log |S_{mp}(\sigma_0)| = \frac{1}{\pi} \int_{-\infty}^\infty \log |S(j\omega)| \frac{\sigma_0}{\sigma_0^2 + \omega^2} d\omega,$$

or equivalently,

$$\int_0^\infty \log |S(j\omega)| \frac{\sigma_0}{\sigma_0^2 + \omega^2} d\omega = \frac{\pi}{2} \log |S_{mp}(\sigma_0)|.$$

Multiply by $\sigma_0$:

$$\int_0^\infty \log |S(j\omega)| \frac{\sigma_0^2}{\sigma_0^2 + \omega^2} d\omega = \frac{\pi}{2} \sigma_0 \log |S_{mp}(\sigma_0)|.$$

It can be shown that the left-hand side converges to

$$\int_0^\infty \log |S(j\omega)| d\omega$$

as $\sigma_0 \to \infty$. [The idea is that for very large $\sigma_0$ the function

$$\frac{\sigma_0^2}{\sigma_0^2 + \omega^2}$$

equals nearly 1 up to large values of $\omega$. On the other hand, $\log |S(j\omega)|$ tends to zero as $\omega$ tends to $\infty$.] So it remains to show that

$$\lim_{\sigma \to \infty} \frac{\sigma}{2} \log |S_{mp}(\sigma)| = (\log \mathrm{e}) (\sum \mathrm{Re} p_i). \tag{6.8}$$

We can write

$$S = S_{ap} S_{mp},$$

where

$$S_{ap}(s) = \prod_i \frac{s - p_i}{s + \overline{p}_i}.$$

It is claimed that

$$\lim_{\sigma \to \infty} \sigma \ln S(\sigma) = 0.$$

To see this, note that since $L$ has relative degree at least 2 we can write

$$L(\sigma) \approx \frac{c}{\sigma^k} \text{ as } \sigma \to \infty$$

for some constant $c$ and some integer $k \geq 2$. Thus as $\sigma \to \infty$

$$\sigma \ln S(\sigma) = -\sigma \ln[1 + L(\sigma)] \approx -\sigma \ln\left(1 + \frac{c}{\sigma^k}\right).$$

Now use the Maclaurin's series

$$\ln(1 + x) = x - \frac{x^2}{2} + \cdots \tag{6.9}$$

to get

$$\sigma \ln S(\sigma) \approx -\sigma\left(\frac{c}{\sigma^k} - \cdots\right).$$

The right-hand side converges to zero as $\sigma$ tends to $\infty$. This proves the claim.

In view of the claim, to prove (6.8) it remains to show that

$$\lim_{\sigma \to \infty} \frac{\sigma}{2} \ln \left|[S_{ap}(\sigma)^{-1}]\right| = \sum \text{Re} p_i. \tag{6.10}$$

Now

$$\ln(S_{ap}(\sigma)^{-1}) = \ln \prod_i \frac{\sigma + \overline{p}_i}{\sigma - p_i} = \sum_i \ln \frac{\sigma + \overline{p}_i}{\sigma - p_i},$$

so to prove (6.10) it suffices to prove

$$\lim_{\sigma \to \infty} \frac{\sigma}{2} \ln \left|\frac{\sigma + \overline{p}_i}{\sigma - p_i}\right| = \text{Re} p_i. \tag{6.11}$$

Let $p_i = x + jy$ and use (6.9) again as follows:

$$\begin{aligned}
\frac{\sigma}{2} \ln \left|\frac{\sigma + \overline{p}_i}{\sigma - p_i}\right| &= \frac{\sigma}{2} \ln \left|\frac{1 + \overline{p}_i \sigma^{-1}}{1 - p_i \sigma^{-1}}\right| \\
&= \frac{\sigma}{4} \ln \frac{(1 + x\sigma^{-1})^2 + (y\sigma^{-1})^2}{(1 - x\sigma^{-1})^2 + (y\sigma^{-1})^2} \\
&= \frac{\sigma}{4} \left\{\ln[(1 + x\sigma^{-1})^2 + (y\sigma^{-1})^2] - \ln[(1 - x\sigma^{-1})^2 + (y\sigma^{-1})^2]\right\} \\
&= \frac{\sigma}{4} \left\{2\frac{x}{\sigma} + 2\frac{x}{\sigma} + \cdots\right\} \\
&= x + \cdots \\
&= \text{Re} p_i + \cdots .
\end{aligned}$$

Letting $\sigma \to \infty$ gives (6.11). ■

**Example** Take the plant and controller

$$P(s) = \frac{1}{(s-1)(s+2)}, \quad C(s) = 10.$$

The feedback system is internally stable and $L$ has relative degree 2. The plot of $|S(j\omega)|$, log scale, versus $\omega$, linear scale, is shown in Figure 6.1. The area below the line $|S| = 1$ is negative, the area above, positive. The theorem says that the net area is positive, equaling

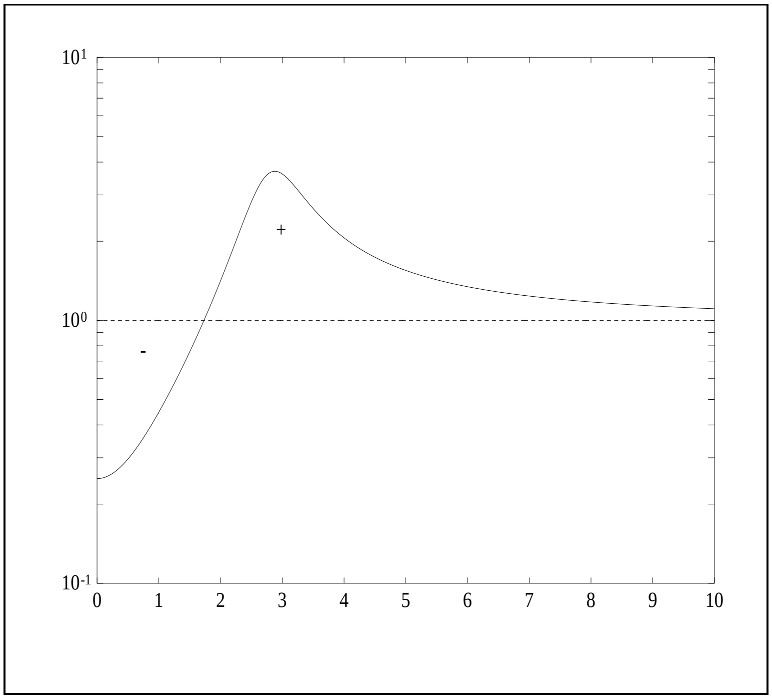


Figure 6.1: $|S(j\omega)|$, log scale, versus $\omega$, linear scale.

$$\pi(\log \mathrm{e})(\sum \mathrm{Re} p_i) = \pi(\log \mathrm{e}).$$

So the negative area, required for good tracking over some frequency range, must unavoidably be accompanied by some positive area.

The waterbed effect applies to non-minimum-phase systems, whereas the area formula applies in general (except for the relative degree assumption). In particular, the area formula does not itself imply a peaking phenomenon, only an area conservation. However, one can infer a type of peaking phenomenon from the area formula when another constraint is imposed, namely, controller bandwidth, or more precisely, the bandwidth of the loop transfer function $PC$. For example, suppose that the constraint is

$$|PC| < \frac{1}{\omega^2}, \quad \forall \omega \geq \omega_1,$$

where $\omega_1 > 1$. This is one way of saying that the loop bandwidth is constrained to be $\leq \omega_1$. Then for $\omega \geq \omega_1$

$$|S| \leq \frac{1}{1-|PC|} < \frac{1}{1-\omega^{-2}} = \frac{\omega^2}{\omega^2-1}.$$

Hence

$$\int_{\omega_1}^{\infty} \log|S(j\omega)|d\omega \le \int_{\omega_1}^{\infty} \log\frac{\omega^2}{\omega^2-1}d\omega.$$

The latter integral—denote it by $I$—is finite. This is proved by the following computation:

$$\begin{aligned} I &= \frac{1}{\ln 10}\int_{\omega_1}^{\infty} \ln\frac{1}{1-\omega^{-2}}d\omega \\ &= -\frac{1}{\ln 10}\int_{\omega_1}^{\infty} \ln(1-\omega^{-2})d\omega \\ &= \frac{1}{\ln 10}\int_{\omega_1}^{\infty} \left(\omega^{-2}+\frac{1}{2}\omega^{-4}+\frac{1}{3}\omega^{-6}+\cdots\right)d\omega \\ &= \frac{1}{\ln 10}\left(\omega_1^{-1}+\frac{1}{2\times 3}\omega_1^{-3}+\frac{1}{3\times 5}\omega_1^{-5}+\cdots\right) \\ &< \infty. \end{aligned}$$

Hence the possible positive area over the interval $[\omega_1, \infty)$ is limited. Thus if $|S|$ is made smaller and smaller over some subinterval of $[0, \omega_1]$, incurring a larger and larger debt of negative area, then $|S|$ must necessarily become larger and larger somewhere else in $[0, \omega_1]$. Roughly speaking, with a loop bandwidth constraint the waterbed effect applies even to minimum-phase plants.

## Exercises

1. Prove the statement about uniqueness in Lemma 2.

2. True or false: For every $\delta > 1$ there exists an internally stabilizing controller such that $\|T\|_\infty < \delta$.

3. Regarding inequality (6.3), the implication is that good tracking is impossible if $P$ has a right half-plane zero where $|W_1|$ is not small. This problem is an attempt to see this phenomenon more precisely by studying $|W_1(z)|$ as a function of $z$ for a typical weighting function. Take $W_1$ to be a third-order Butterworth filter with cutoff frequency 1 rad/s. Plot

$$|W_1(0.1+j\omega)| \quad \text{versus} \quad \omega$$

for $\omega$ going from 0 up to where $|W_1| < 0.01$. Repeat for abscissae of 1 and 10.

4. Let

$$P(s) = 4\frac{s-2}{(s+1)^2}.$$

Suppose that $C$ is an internally stabilizing controller such that

$$\|S\|_\infty = 1.5.$$

Give a positive lower bound for

$$\max_{0\le\omega\le 0.1} |S(j\omega)|.$$

5. Define $\epsilon := \|W_1 S\|_\infty$ and $\delta := \|CS\|_\infty$. So $\epsilon$ is a measure of tracking performance, while $\delta$ measures control effort; note that $CS$ equals the transfer function from reference input $r$ to plant input. In a design we would like $\epsilon < 1$ and $\delta$ not too large. Derive the following inequality, showing that $\epsilon$ and $\delta$ cannot both be very small in general: For every point $s_0$ with $\mathrm{Re} s_0 \geq 0$,
$$|W_1(s_0)| \leq \epsilon + |W_1(s_0)P(s_0)|\delta.$$

6. Let $\omega$ be a frequency such that $j\omega$ is not a pole of $P$. Suppose that
$$\epsilon := |S(j\omega)| < 1.$$
Derive a lower bound for $|C(j\omega)|$ that blows up as $\epsilon \to 0$. Conclusion: Good tracking at a particular frequency requires large controller gain at this frequency.

7. Suppose that the plant transfer function is
$$P(s) = \frac{1}{s^2 - s + 4}.$$
We want the controller $C$ to achieve the following:

    internal stability,

    $|S(j\omega)| \leq \epsilon$ for $0 \leq \omega < 0.1$,

    $|S(j\omega)| \leq 2$ for $0.1 \leq \omega < 5$,

    $|S(j\omega)| \leq 1$ for $5 \leq \omega < \infty$.

    Find a (positive) lower bound on the achievable $\epsilon$.

## Notes and References

This chapter is in the spirit of Bode's book (Bode, 1945) on feedback amplifiers. Bode showed that electronic amplifiers must have certain inherent properties simply by virtue of the fact that stable network functions are analytic, and hence have certain strong properties. Bode's work was generalized to control systems by Bower and Schultheiss (1961) and Horowitz (1963).

The interpolation conditions (6.1) and (6.2) were obtained by Raggazini and Franklin (1958). These constraints on $S$ and $T$ are essentially equivalent to the controller parametrization in Theorem 5.2. Inequality (6.3) was noted, for example, by Zames and Francis (1983). The waterbed effect, Theorem 1, was proved by Francis and Zames (1984), but the derivation here is due to Freudenberg and Looze (1985). The area formula, Theorem 2, was proved by Bode (1945) in case $L$ is stable, and by Freudenberg and Looze (1985) in the general case. An excellent discussion of performance limitations may be found in Freudenberg and Looze (1988).
